# Risk for diagnosis or treatment of mood or anxiety disorders in adults after SARS-CoV-2 infection, 2020–2022

**DOI:** 10.1038/s41380-024-02414-x

**Published:** 2024-01-18

**Authors:** Christina X. Wang, Rhea Kohli, Veronica R. Olaker, Pauline Terebuh, Rong Xu, David C. Kaelber, Pamela B. Davis

**Affiliations:** 1 Center for Artificial Intelligence in Drug Discovery, Case Western Reserve University School of Medicine, Cleveland, OH USA; 2 Center for Community Health Integration, Case Western Reserve University School of Medicine, Cleveland, OH USA; 3 The Center for Clinical Informatics Research and Education and the Departments of Internal Medicine, Pediatrics, and Population and Quantitative Health Sciences, Case Western Reserve University, The MetroHealth System, Cleveland, OH USA

**Keywords:** Depression, Neuroscience

## Abstract

COVID-19 is associated with increased risks for mood or anxiety disorders, but it remains uncertain how the association evolves over time or which patient groups are most affected. We conducted a retrospective cohort study using a nationwide database of electronic health records to determine the risk of depressive or anxiety disorder diagnoses after SARS-CoV-2 infection by 3-month blocks from January 2020 to April 2022. The study population comprised 822,756 patients (51.8% female; mean age 42.8 years) with COVID-19 and 2,034,353 patients with other respiratory tract infections (RTIs) (53.5% female, mean age 30.6 years). First time diagnoses of depressive or anxiety disorders 14 days to 3 months after infection, as well as new or new plus recurrent prescriptions of antidepressants or anxiolytics, were compared between propensity score matched cohorts using Kaplan-Meier survival analysis, including hazard ratio (HR) and 95% confidence interval (CI). Risk of a new diagnosis or prescription was also stratified by age, sex, and race to better characterize which groups were most affected. In the first three months of the pandemic, patients infected with SARS-CoV-2 had significantly increased risk of depression or anxiety disorder diagnosis (HR 1.65 [95% CI, 1.30-2.08]). October 2021 to January 2022 (HR, 1.12 [95% CI, 1.06–1.18]) and January to April 2022 (HR, 1.08 [95% CI, 1.01–1.14]). Similar temporal patterns were observed for antidepressant and anxiolytic prescriptions, when the control group was patients with bone fracture, when anxiety and depressive disorders were considered separately, when recurrent depressive disorder was tested, and when the test period was extended to 6 months. COVID-19 patients ≥65 years old demonstrated greatest absolute risk at the start of the pandemic (6.8%), which remained consistently higher throughout the study period (HR, 1.20 [95% CI, 1.13–1.27]), and overall, women with COVID-19 had greater risk than men (HR 1.35 [95% CI 1.30–1.40]).

## Introduction

Reports of increased mood and anxiety disorders during the COVID-19 pandemic raised interest in the mental health impact of COVID-19. The World Health Organization reported a 27.6% increase in depression and a 25.6% increase in anxiety in 2020 compared to 2019 [1], and the Centers for Disease Control and Prevention reported significant increases in anxiety and depression from August 2020 to February 2021 [2]. Some studies have suggested that this increase may be due to a direct association between COVID-19 infection and psychiatric sequelae [3–6]; however, others suggest that this psychiatric risk may vary with time. In a meta-analysis of cohort studies comparing mental health in the general population before and after the pandemic, Robinson et al. found that increases in symptoms were only significant at the start of the pandemic and became non-significant by May–July of 2020 [7]. Throughout the pandemic, public perception and pandemic response have been dynamic. Whether the risk of mood and anxiety disorders in patients infected with COVID-19 is also dynamic remains an important question. In addition, some studies have suggested that certain patient groups, such as young adults and women, are at greater risk for psychiatric sequelae after COVID-19 [2, 8, 9], although the impact of COVID-19 on excess death and hospitalization was by far the greatest among the elderly [10], among men compared to women [11], and among Black patients compared to White patients [12]. To examine these questions in a large diverse sample of patients during different time periods during the pandemic, we evaluated the risk for ICD-10 diagnoses of anxiety and depressive disorders as well as prescriptions for anxiolytic medications and antidepressants following COVID-19 in successive time blocks during the pandemic. We further stratified the sample by age, sex, and race to address the questions raised about these patient groups. We compared patients following COVID-19 to patients during the same time periods who had either other respiratory infections or bone fractures in order to control for the stress of serious infectious or non-infectious illness.

## Methods

### Database description

We used the TriNetX Analytics Platform to conduct this study, accessed from February 6 to March 17, 2023. We used the Research USA No Date Shift Network, which contains deidentified electronic health record (EHR) data from over 60 million patients from 34 United States health care organizations. Data are deidentified per the Health Insurance Portability and Accountability Act (HIPAA) criteria—Section §164.514(a) of the HIPAA Privacy Rule. The MetroHealth System institutional review board has deemed studies using TriNetX data not to be human participant research and exempt from review. Patient consent was waived by the MetroHealth System institutional review board based on the deidentification of the data. TriNetX also has an exemption from the Western institutional review board based on deidentification of the data in a HIPAA–compliant manner. This study followed the Strengthening the Reporting of Observational Studies in Epidemiology (STROBE) reporting guideline.

### Study population and cohort definitions

The total study population consisted of 2,857,109 patients: 822,756 with COVID-19 and 2,034,353 with other respiratory tract infections (RTIs) diagnosed between January 2020 and April 2022 and no prior documentation of COVID-19 (based on an encounter diagnosis code for COVID-19, positive result for SARS-CoV-2 detection test, or positive antibody test result for SARS-CoV-2 before December 11, 2020 to capture COVID-19 infection before vaccines were introduced). A COVID-19 diagnosis was defined by the *International Statistical Classification of Diseases and Related Health Problems, Tenth Revision (ICD-10)* code for COVID-19 (U07.1). Additional *ICD-10* codes were included for the first-time block (January 1 to April 1, 2020) to capture early cases prior to the institution of the *ICD-10* code for COVID-19 (U07.1) (eMethods in Supplement 1). Additional analyses were run where a COVID-19 diagnosis was defined by either the *ICD-10* code for COVID-19 (U07.1) or a positive RNA test result for SARS-CoV-2 (eMethods in Supplement 1). Diagnoses for other RTIs were defined by the *ICD-10* codes for acute upper RTIs (J00–J06), influenza and pneumonia, (J09–J18), or other acute lower RTIs (J20–J22), as adapted from Taquet et al. [3]. To test the robustness of our findings, we also used bone fracture as a control index event (eMethods and eFig. 3 in Supplement 1).

**Fig. 1:**
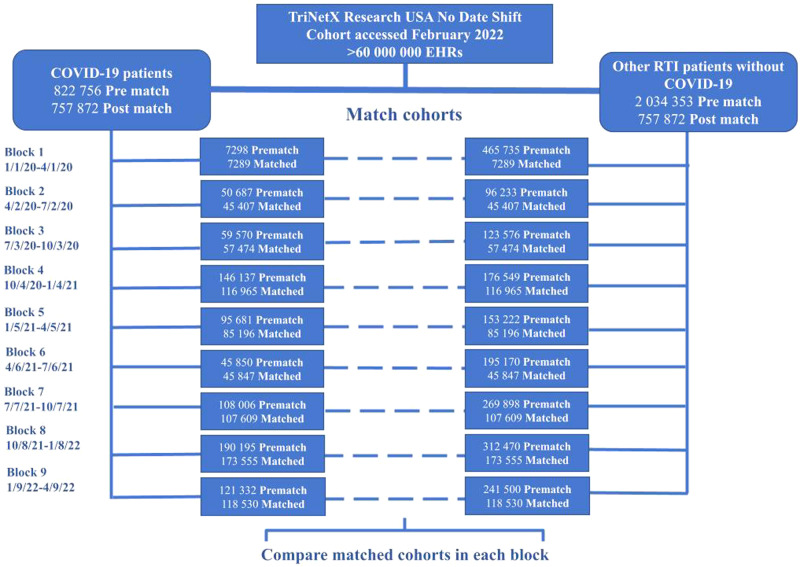
Flow diagram for building cohorts. The number of patients in the COVID-19 and other respiratory infection cohorts before and after propensity score matching is shown for each of the 9 time blocks that were analyzed. The dates that comprise each 3-month time block are shown in the first column.

COVID-19 cohorts were defined by 3-month time intervals such that Block 1 contained patients diagnosed with COVID-19 from January 1 to April 1, 2020, Block 2 contained patients from April 2 to July 2, 2020, and so on; this yielded nine cohorts spanning January 1, 2020, to April 9, 2022. Other RTI cohorts were constructed in the same way to generate nine corresponding control cohorts (Fig. 1). To minimize overlap between adjacent COVID-19 cohorts, we excluded patients who had a COVID-19 *ICD-10* code or positive RNA test result within the previous 3-month time interval. To capture new mood and anxiety disorders, we excluded patients who had a depressive or anxiety disorder diagnosis up to the beginning of the follow-up period or 14 days after the index event. To capture mood and anxiety disorder exacerbations in addition to new disorders, we allowed patients with prior antidepressant or anxiolytic prescriptions up until 1 year before the index event to be included.

### Covariates and outcomes

COVID-19 and other RTI patients were matched for age, sex, race, ethnicity, physiologic [3] and psychiatric risk factors [12] for severe COVID-19 illness, family or personal history of mental illness, recorded COVID-19 vaccination, hospitalization, and socioeconomic status (Table 1). The TriNetX built-in propensity score matching function was used (1:1 matching using a nearest neighbor greedy matching algorithm with a caliper of 0.25 times the SD). To measure the association between new depressive and anxiety disorders and COVID-19, the primary outcome was a first-time diagnosis of a depressive disorder (F30-F39) or anxiety disorder (F40-F48). We also used first-time prescriptions of antidepressants or anxiolytics to measure the association between new depressive and anxiety disorders and COVID-19. This analysis was stratified by age (18–35, 36–49, 50–64, and ≥65 years old), sex (female and male), and race (Black and White; there were insufficient numbers of Asian, American Indian or Alaska Native, and Native Hawaiian or Other Pacific Islander patients for statistical analysis). Time blocks were then combined in order to draw comparisons over the entire study period between COVID-19 patients by age (≥65 vs. <65 years old), sex (female vs. male), and race (Black vs. White). Additional analyses were run with depressive disorder and anxiety disorder as individual outcomes. To measure the association between new or recurrent depressive and anxiety disorders and COVID-19, the primary outcome was a new or recurrent antidepressant or anxiolytic prescription. Additional analyses were done using the ICD-10 code for recurrent major depressive disorder (F33).

**Table 1:** Baseline patient characteristics before and after matching for block 1.

	Before matching			After matching		
	Cohort, No. (%)			Cohort, No. (%)		
Characteristics	COVID-19	Other RTI	SMD	COVID-19	Other RTI	SMD
Total number	7298	465,735		7289	7289	
Age at index, mean (SD), y	46.0 (21.5)	28.8 (24.7)	0.74	46.0 (21.5)	47.4 (23.1)	0.06
Current age, mean (SD), y	48.9 (21.4)	31.7 (24.7)	0.74	48.8 (21.4)	50.3 (22.9)	0.07
Gender						
Female	3495 (47.9)	248,485 (53.4)	0.11	3492 (47.9)	3533 (48.5)	0.01
Male	3800 (52.1)	217,188 (46.6)	0.11	3794 (52.1)	3754 (51.5)	0.01
Unknown	10 (0.1)	62 (0.0)	0.05	10 (0.1)	10 (0.1)	0.00
Race						
White	3225 (44.2)	299,112 (64.2)	0.41	3223 (44.2)	3284 (45.1)	0.02
Black or African American	2275 (31.2)	81,420 (17.5)	0.32	2274 (31.2)	2191 (30.1)	0.02
Asian	245 (3.4)	14,137 (3.0)	0.02	245 (3.4)	251 (3.4)	0.00
American Indian or Alaska Native	20 (0.3)	1424 (0.3)	0.01	20 (0.3)	24 (0.3)	0.01
Native Hawaiian or Other Pacific Islander	14 (0.2)	816 (0.2)	0.00	14 (0.2)	10 (0.1)	0.01
Unknown race	1519 (20.8)	68,826 (14.8)	0.16	1513 (20.8)	1532 (21.0)	0.01
Ethnicity						
Hispanic or Latino	1155 (15.8)	61,498 (13.2)	0.07	1150 (15.8)	1132 (15.5)	0.01
Not Hispanic or Latino	4677 (64.1)	315,721 (67.8)	0.08	4673 (64.1)	4681 (64.2)	0.00
Unknown ethnicity	1466 (20.1)	88,516 (19.0)	0.03	1466 (20.1)	1476 (20.3)	0.00
Overweight and obesity	1010 (13.8)	43,335 (9.3)	0.14	1005 (13.8)	917 (12.6)	0.04
Hypertensive diseases	2050 (28.1)	71,135 (15.3)	0.31	2043 (28.0)	1894 (26.0)	0.05
Type 1 diabetes mellitus	130 (1.8)	3561 (0.8)	0.09	130 (1.8)	107 (1.5)	0.02
Type 2 diabetes mellitus	1024 (14.0)	29,396 (6.3)	0.26	1020 (14.0)	901 (12.4)	0.05
Bronchitis, not specified as acute or chronic	242 (3.3)	20,753 (4.5)	0.06	242 (3.3)	227 (3.1)	0.01
Simple and mucopurulent chronic bronchitis	15 (0.2)	1340 (0.3)	0.02	15 (0.2)	12 (0.2)	0.01
Unspecified chronic bronchitis	29 (0.4)	1334 (0.3)	0.02	29 (0.4)	22 (0.3)	0.02
Emphysema	136 (1.9)	4525 (1.0)	0.08	136 (1.9)	121 (1.7)	0.02
Other chronic obstructive pulmonary disease	298 (4.1)	11,444 (2.5)	0.09	297 (4.1)	279 (3.8)	0.01
Asthma	661 (9.1)	53,772 (11.5)	0.08	661 (9.1)	645 (8.8)	0.01
Bronchiectasis	40 (0.5)	2007 (0.4)	0.02	40 (0.5)	37 (0.5)	0.01
Other forms of heart disease	1244 (17.0)	39,676 (8.5)	0.26	1238 (17.0)	1062 (14.6)	0.07
Hypertensive chronic kidney disease	268 (3.7)	5076 (1.1)	0.17	267 (3.7)	220 (3.0)	0.04
Hepatic failure, not elsewhere classified	25 (0.3)	685 (0.1)	0.04	25 (0.3)	18 (0.2)	0.02
Chronic hepatitis, not elsewhere classified	10 (0.1)	256 (0.1)	0.03	10 (0.1)	11 (0.2)	0.00
Fibrosis and cirrhosis of liver	55 (0.8)	1848 (0.4)	0.05	55 (0.8)	55 (0.8)	0.00
Fatty (change of) liver, not elsewhere classified	178 (2.4)	6145 (1.3)	0.08	177 (2.4)	164 (2.3)	0.01
Chronic passive congestion of liver	34 (0.5)	1031 (0.2)	0.04	34 (0.5)	36 (0.5)	0.00
Portal hypertension	19 (0.3)	650 (0.1)	0.03	19 (0.3)	18 (0.2)	0.00
Other specified diseases of liver	135 (1.9)	4386 (0.9)	0.08	135 (1.9)	128 (1.8)	0.01
Cerebral infarction	249 (3.4)	5833 (1.3)	0.14	247 (3.4)	196 (2.7)	0.04
Vascular dementia	24 (0.3)	275 (0.1)	0.06	22 (0.3)	21 (0.3)	0.00
Dementia in other diseases classified elsewhere	23 (0.3)	533 (0.1)	0.04	23 (0.3)	17 (0.2)	0.02
Unspecified dementia	64 (0.9)	1211 (0.3)	0.08	62 (0.9)	52 (0.7)	0.02
Alzheimer’s disease	21 (0.3)	545 (0.1)	0.04	21 (0.3)	18 (0.2)	0.01
Frontotemporal dementia	10 (0.1)	34 (0.0)	0.05	10 (0.1)	10 (0.1)	0.00
Neurocognitive disorder with Lewy bodies	10 (0.1)	40 (0.0)	0.05	10 (0.1)	0 (0.0)	0.05
Neoplasms	1365 (18.7)	58,395 (12.5)	0.17	1359 (18.6)	1294 (17.8)	0.02
Malignant neoplasms of lymphoid, hematopoietic and related tissue	128 (1.8)	3557 (0.8)	0.09	128 (1.8)	123 (1.7)	0.01
Rheumatoid arthritis with rheumatoid factor	26 (0.4)	1189 (0.3)	0.02	26 (0.4)	31 (0.4)	0.01
Other rheumatoid arthritis	87 (1.2)	3741 (0.8)	0.04	87 (1.2)	81 (1.1)	0.01
Systemic lupus erythematosus (SLE)	40 (0.5)	1317 (0.3)	0.04	40 (0.5)	30 (0.4)	0.02
Psoriasis	53 (0.7)	3412 (0.7)	0.00	53 (0.7)	51 (0.7)	0.00
Certain disorders involving the immune mechanism	222 (3.0)	5696 (1.2)	0.13	222 (3.0)	189 (2.6)	0.03
Family history of mental and behavioral disorders	75 (1.0)	3629 (0.8)	0.03	73 (1.0)	63 (0.9)	0.01
Persons with potential health hazards related to socioeconomic and psychosocial circumstances	211 (2.9)	10,778 (2.3)	0.04	209 (2.9)	143 (2.0)	0.06
Personal history of other mental and behavioral disorders	22 (0.3)	908 (0.2)	0.02	22 (0.3)	21 (0.3)	0.00
Nicotine dependence	451 (6.2)	25,469 (5.5)	0.03	451 (6.2)	460 (6.3)	0.01
Alcohol related disorders	252 (3.5)	4720 (1.0)	0.17	247 (3.4)	205 (2.8)	0.03
Other psychoactive substance related disorders	209 (2.9)	3111 (0.7)	0.17	201 (2.8)	133 (1.8)	0.06
Cannabis related disorders	55 (0.8)	2180 (0.5)	0.04	55 (0.8)	45 (0.6)	0.02
Other stimulant related disorders	26 (0.4)	1263 (0.3)	0.02	26 (0.4)	22 (0.3)	0.01
Opioid related disorders	38 (0.5)	1508 (0.3)	0.03	38 (0.5)	30 (0.4)	0.02
Cocaine related disorders	34 (0.5)	823 (0.2)	0.05	34 (0.5)	22 (0.3)	0.03
Hallucinogen related disorders	12 (0.2)	189 (0.0)	0.04	11 (0.2)	10 (0.1)	0.00
Inhalant related disorders	11 (0.2)	458 (0.1)	0.01	11 (0.2)	11 (0.2)	0.00
Sedative, hypnotic, or anxiolytic related disorders	10 (0.1)	242 (0.1)	0.03	10 (0.1)	10 (0.1)	0.00
Unspecified psychosis not due to a substance or known physiological condition	19 (0.3)	426 (0.1)	0.04	19 (0.3)	14 (0.2)	0.01
Schizophrenia	16 (0.2)	430 (0.1)	0.03	16 (0.2)	10 (0.1)	0.02
Schizoaffective disorders	10 (0.1)	195 (0.0)	0.03	10 (0.1)	10 (0.1)	0.00
Delusional disorders	10 (0.1)	189 (0.0)	0.03	10 (0.1)	10 (0.1)	0.00
Brief psychotic disorder	10 (0.1)	45 (0.0)	0.05	10 (0.1)	0 (0.0)	0.05
Other psychotic disorder not due to a substance or known physiological condition	10 (0.1)	11 (0.0)	0.05	10 (0.1)	10 (0.1)	0.00
Schizotypal disorder	0 (0.0)	10 (0.0)	0.01	0 (0.0)	0 (0.0)	
Shared psychotic disorder	0 (0.0)	10 (0.0)	0.01	0 (0.0)	0 (0.0)	

*RTI* respiratory tract infections, *SMD* standard mean difference.

### Statistical analysis

We compared the risk of mental health outcomes between COVID-19 patients and other RTI patients for each of 9 time blocks using HRs and 95% CIs. Our outcome follow-up period was 14 days to 3 months after the index event (COVID-19 or non-COVID-19 RTI diagnosis) or 14 days to 6 months after the initial index event. Statistical analyses were conducted in the TriNetX Analytics Platform. Kaplan-Meier analysis was used to estimate the probability of clinical outcomes. Cox proportional hazards regression analysis was used to compare the matched cohorts. The proportional hazard assumption was tested using the generalized Schoenfeld approach. The TriNetX Platform calculates HRs and associated 95% CIs, using R’s Survival package, version 3.2-3 (R Group for Statistical Computing). Hypothesis tests were 2-sided, and results were deemed statistically significant at two-sided *P* < 0.05.

## Results

This study population was comprised of 822,756 patients with COVID-19 (51.8% female patients; 60.0% White; mean [SD] age at index, 42.8 [21.4] years) and 2,034,353 patients with other RTIs who were never diagnosed with COVID-19 (53.5% female patients; 65.3% White; mean [SD] age at index, 30.6 [24.9] years). After matching, the total population was 1,515,744, with 757,872 patients in each cohort (Fig. 1). Figure 1 also shows the numbers of patients in each time block. The Table shows the baseline patient characteristics before and after matching for both cohorts in Block 1; patient characteristics for the remaining time blocks are available in eTables 1–9 in Supplement 1.

**Fig. 2:**
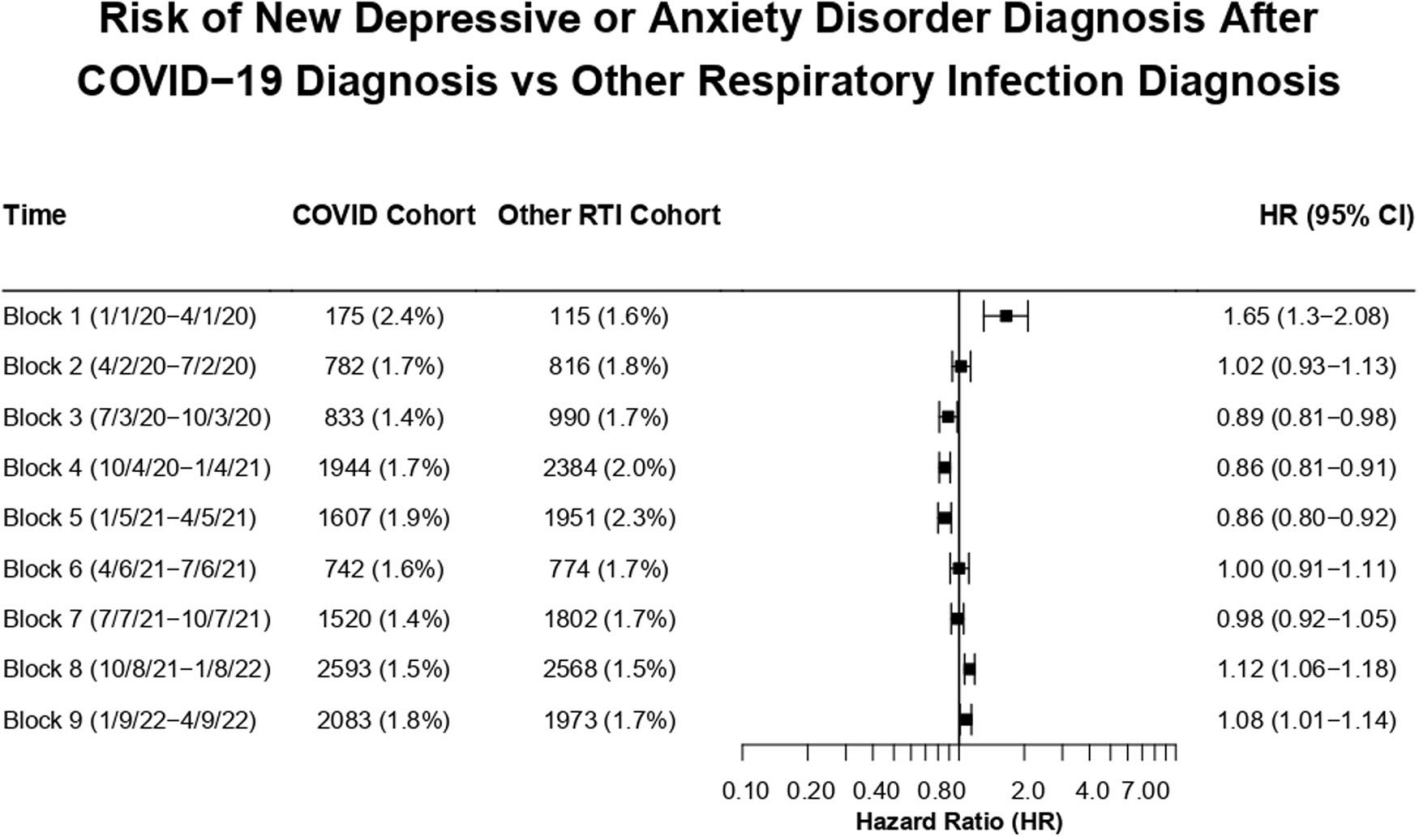
Risk of new depressive or anxiety disorder diagnosis after COVID-19 diagnosis vs. other respiratory infection diagnosis. Hazard ratios (HRs) for COVID-19 versus other respiratory tract infection cohorts across the 9 time blocks for the outcome of a first-time *ICD-10* encounter diagnosis of a depressive disorder (F30-F39) or anxiety disorder (F40–F48).

The HR for a new depressive or anxiety disorder ICD-10 diagnosis code was significantly increased for patients after COVID-19 compared to matched patients after RTI from January to April 2020 (Block 1: HR, 1.65 [95% CI, 1.30–2.08]) (Fig. 2). No significantly increased HR was observed from April 2020 to October 2021(Blocks 2–7). A small, but statistically significant elevated HR was observed late in the study period from October 2021 to April 2022 (Block 8: HR, 1.12 [95% CI, 1.06–1.18]; Block 9: HR, 1.08 [95% CI, 1.01–1.14]). A similar pattern was observed for the diagnosis code for recurrent major depressive disorder (eFig. 1 in Supplement 1).

**Fig. 3:**
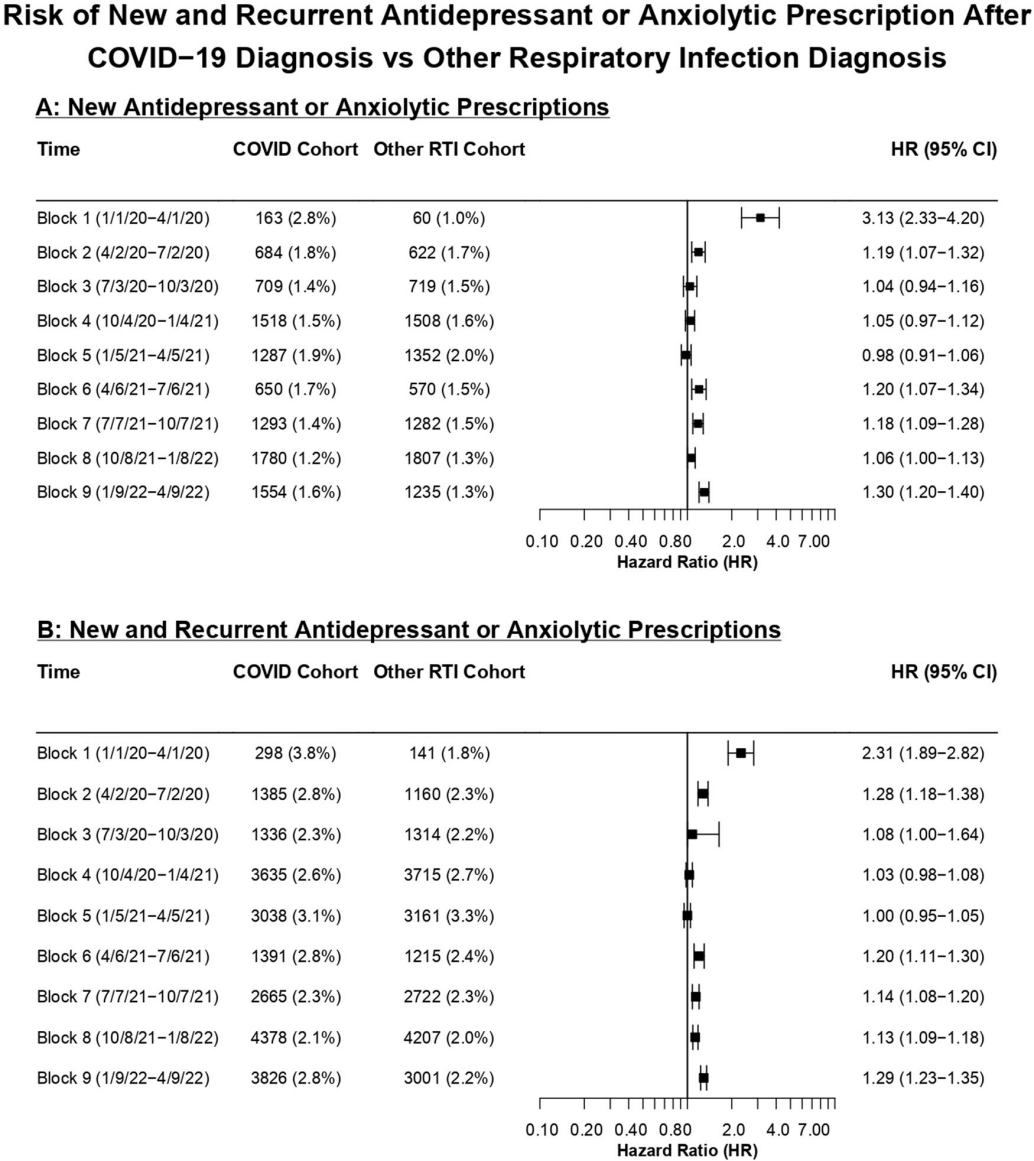
Risk of new and recurrent antidepressant or anxiolytic prescription after COVID-19 diagnosis vs other respiratory infection diagnosis. Hazard ratios (HRs) for COVID-19 versus other respiratory tract infection cohorts across the 9 time blocks for the outcome of an antidepressant or anxiolytic prescription. **A** shows HRs for a first-time prescription. **B** shows HRs for new and recurrent prescriptions.

The HR for a new antidepressant or anxiolytic prescription was significantly increased for patients after COVID-19 from January to April 2020 (Block 1: HR, 3.13 [95% CI, 2.33–4.20]) as well as from April to July 2020, albeit substantially smaller (Block 2: HR, 1.19 [95% CI, 1.07–1.32]) (Fig. 3A). There was no significantly increased HR from July 2020 to April 2021 or from October 2021 to January 2022. A small, but statistically significant increased HR was observed from April to October 2021 (Block 6: HR 1.20, [95% CI, 1.07–1.34]; Block 7: HR 1.18, [95% CI, 1.09–1.28]) and from January to April 2022 (Block 9: HR 1.30, [95% CI, 1.20–1.40]). A similar temporal pattern was observed when including patients who had a prior prescription up until a year prior to COVID-19 diagnosis (Fig. 3B).

Similar temporal patterns in HRs were observed when the COVID-19 cohort included patients who had a positive RNA test result, when the control index event was a bone fracture diagnosis, and when measuring the HR individually for a new mood disorder and anxiety disorder diagnosis (eFigs. 2–5 in Supplement 1). In addition, when the assessment period after the index event was extended to 6 months, the pattern was similar (eFig. 8 in Supplement 1). We also compared patients hospitalized with COVID-19 with those hospitalized with other respiratory infections and found a similar temporal pattern of elevated HR in Block 1 which fell to non-significant levels thereafter except for Block 8 for diagnoses and Block 9 for pharmacologic treatments (eFig. 9, Supplement 1).

**Fig. 4:**
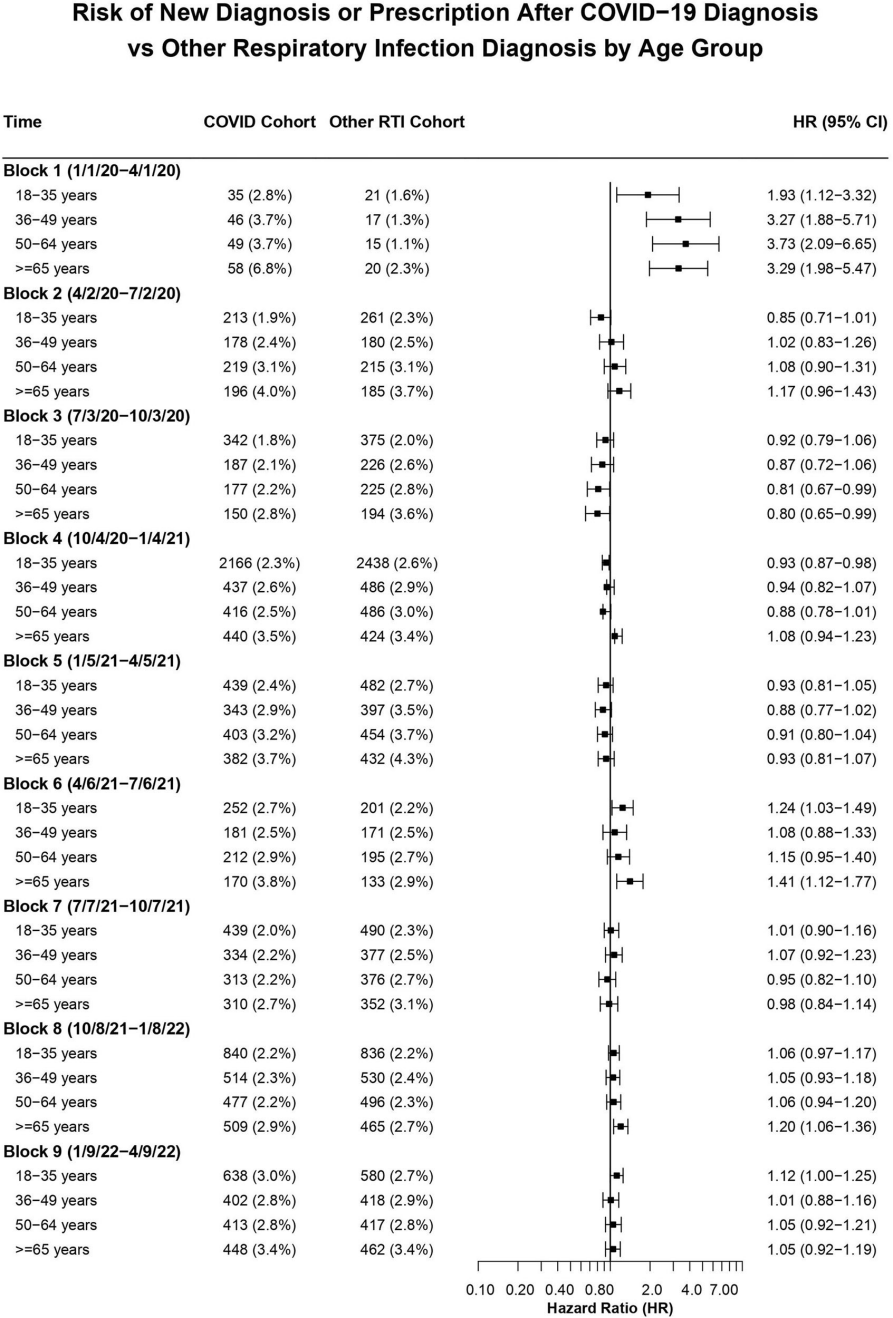
HR and 95% CI for new diagnosis if anxiety or depressive disorder or a new antidepressant or anxiolytic prescription stratified by age for each time block. Hazard ratios (HRs) for COVID-19 versus other respiratory tract infection cohorts stratified by age groups across the 9 time blocks for the outcome of either a first-time *ICD-10* encounter diagnosis of a depressive disorder (F30-F39) or anxiety disorder (F40-F48) or a first-time antidepressant or anxiolytic prescription.

When results of the matched comparison between COVID-19 and non-COVID-19 RTIs were stratified by age, the greatest absolute risk of a new mood or anxiety disorder diagnosis or a new antidepressant or anxiolytic prescription was for adults ≥65 years old who had been diagnosed with COVID-19 during the first 3 months of the pandemic (6.8%, compared to 2.8-3.7% for younger adults). An excess absolute risk for older adults persisted throughout the entire study period, including blocks for which there was no overall increased hazard of mood or anxiety disorders for COVID-19 patients compared to other RTI patients (Fig. 4). Even among patients with non-COVID-19 RTIs, there was a higher absolute risk of mood or anxiety disorders in adults ≥65 years old compared to younger age groups. Among patients with COVID-19 at any time during the study period, there was a significantly increased HR for such a mental health diagnosis for adults ≥65 years old compared to adults <65 years old after matching for all other covariates (HR, 1.20 [95% CI, 1.13–1.27]).

**Fig. 5:**
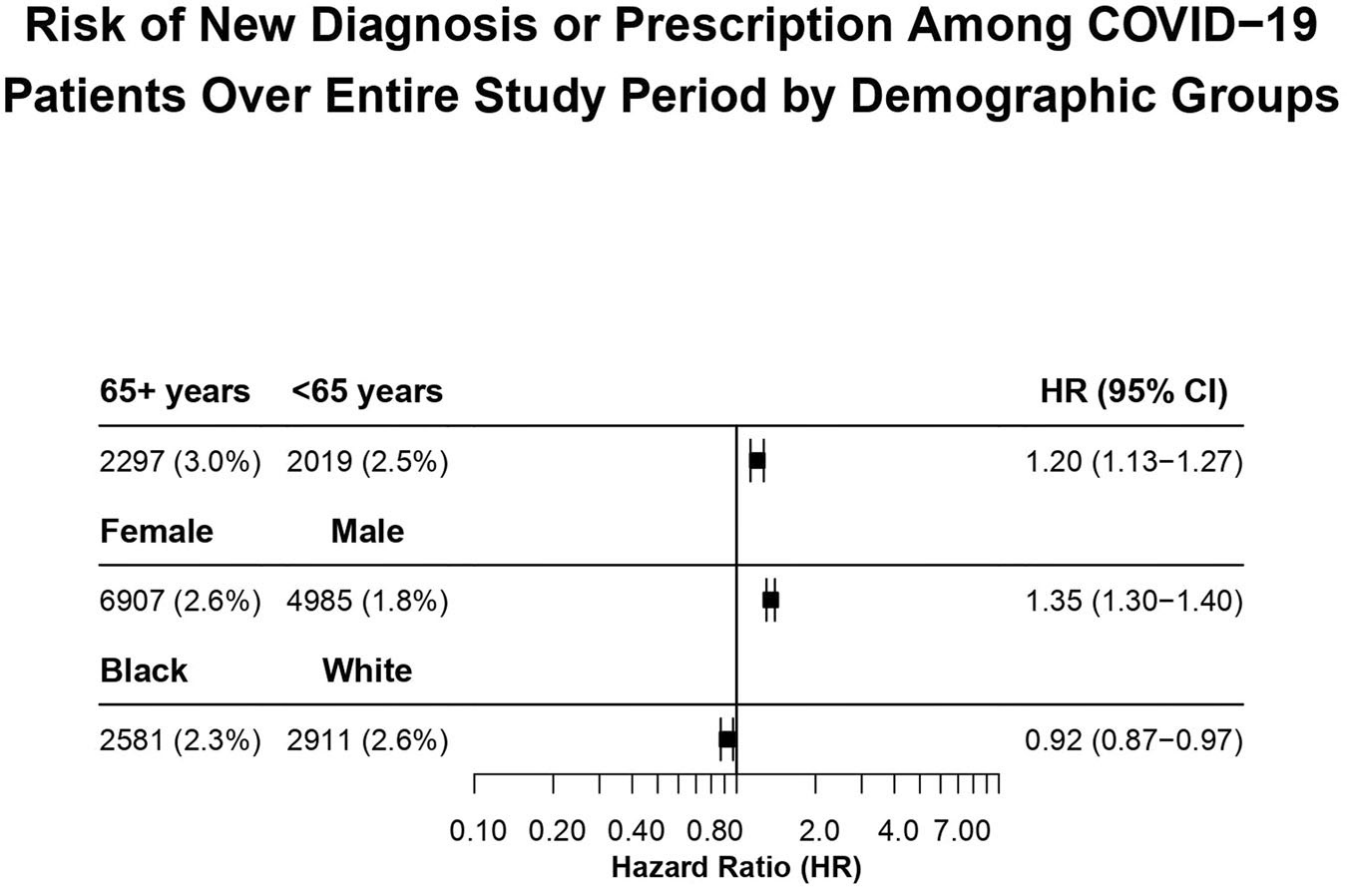
Risk of new diagnosis or prescription among COVID-19 patients over entire study period by demographic groups. Hazard ratios (HRs) for adults ≥65 years old versus <65 years old, females versus males, and Black vs. White patients after COVID-19 across the entire study period for the outcome of either a first-time *ICD-10* encounter diagnosis of a depressive disorder (F30–F39) or anxiety disorder (F40–F48) or a first-time antidepressant or anxiolytic prescription.

Results comparing cohorts by sex and race after COVID-19 were less straightforward. After matching, the absolute risk of a new diagnosis or prescription was roughly equivalent for female patients (3.9%) and male patients (3.8%) after COVID-19 during the first three months of the pandemic; for the remainder of the study period, the absolute risk for female patients was greater. (eFig. 6 in Supplement 1). For patients with COVID-19 at any time during the study period, females compared to males had a significantly increased HR (HR, 1.35 [95% CI, 1.30–1.40]) (Fig. 5). There was no consistent pattern in results stratified by race (eFig. 7 in Supplement 1). A small but significantly decreased HR was observed when comparing Black patients to White patients (HR, 0.92 [95% CI, 0.87–0.97]) (Fig. 5). All HRs are available in eFigs. 1–9 in Supplement 1.

## Discussion

In this retrospective cohort study comparing patients with COVID-19 to control patients without COVID-19, the risk of new or recurrent depressive and anxiety disorders was greatest for patients who contracted COVID-19 in the beginning of the pandemic. Afterward, there was no difference in risk between COVID-19 and control patients for the next 1.5 years and only a mildly elevated risk in late 2021 and early 2022 that was smaller than that observed in the first three months of the pandemic. Our findings align with several studies suggesting that mental health disorders declined in the general population after the beginning of the pandemic [7, 13–15]. Additionally, the temporal patterns found in this study and in our group’s work on alcohol use disorders [15] provide an alternate perspective to study findings on the association between COVID-19 infection and psychiatric sequelae. We provide new support for the suggestion that while a COVID-19 diagnosis may have been associated with increased depressive and anxiety disorders when the pandemic started, this risk declined or was only mildly elevated during its later stages when compared to well-matched controls who had either a non-COVID respiratory infection, or a bone fracture. Although it is possible that the virus itself changed during the pandemic to become less likely to impact anxiety and depression directly in the brain, our temporal findings align well with concerns expressed in the population during the pandemic. While our findings do not exclude the possibility of a direct viral effect of COVID-19 on brain function to increase depressive and anxiety disorders, this temporal pattern suggests that other non-biological factors may predominate.

There are several possible explanations for this dynamic risk. One possibility is that the risk of depressive and anxiety disorders after COVID-19 mirrors changes in the pandemic’s social context. A COVID-19 diagnosis at the beginning of the pandemic was likely very distressing due to the implementation of lockdown and isolation orders and the high rates of hospitalizations and deaths, especially for older Americans who had the greatest risk for severe disease [10]. Over time, changes in pandemic response likely abated this stress through decreases in recommended isolation time [16], reopening of schools and businesses, improved health system preparedness, and employment recovery. Additionally, vaccines and antiviral drugs offered protection and associated relief, which aligns with studies demonstrating that individuals who received the COVID-19 vaccine reported lower symptoms of mood and anxiety disorders [17, 18]. The social context of the pandemic could also help explain the mild elevation in risk seen towards late 2021 and early 2022 when COVID variants were on the rise. Poll data from The Associated Press suggested resurgences in anxiety regarding COVID-19 in August 2021, a time when the Delta variant was causing record hospitalizations after several months of low case numbers [19]. Another potential explanation is that these temporal trends reflect recognized psychological trajectories after mass traumatic events. Prior trauma research has suggested that mood and anxiety disorders typically spike immediately after a crisis and are followed by a return to baseline due to the development of resilience, or the ability to adapt to serious stressors [20–22]. For example, a similar return to baseline in psychiatric disorders was observed following the 2003 SARS outbreak [23, 24]. Finally, patients experiencing post-acute sequelae of COVID-19 (colloquially termed “long COVID”) may have been mistakenly diagnosed with a depressive or anxiety disorder in the beginning of the pandemic since it can present with similar symptoms such as fatigue, decreased concentration, sleep disturbances, and loss of appetite [25, 26]. As health providers and the general public became more familiar with the long-term effects of COVID-19 infection [27], the diagnosis assigned to them might have increasingly become “long COVID” rather than a depressive or anxiety disorder, thus leading to fewer diagnoses as the pandemic went on. The formal diagnostic code for this syndrome, however, was not approved for use until October 2021, and thus would have affected largely Blocks 8 and 9. However, if this were a major factor impacting diagnoses of depressive disorders or anxiety, we would have expected the numbers of such diagnoses following COVID-19 to drop when an alternative diagnostic code was available, but in fact, the number of new diagnoses and new and recurrent prescriptions increased in Blocks 8 and 9 compared to Blocks 6 and 7, among patients following both COVID-19 and other respiratory infections, and the HR for COVID-19 infections actually increased in Blocks 8 and 9.

Our results appear to be robust because the pattern of risk we observed was similar whether the comparison group was ORI or bone fractures, whether the outcome measure was ICD-10 diagnostic codes or prescriptions for appropriate medications, when depressive disorders, anxiety, and recurrent depressive disorders were analyzed separately, when the observation period following the infection was either three or 6 months, and when COVID-19 was defined strictly by an RNA test.

Alternate explanations we considered for the observed decrease in risk for depressive disorders and anxiety later in the pandemic included the fact that COVID-19 infection led to less severe clinical presentations later in the pandemic due to different SARS-CoV-2 variants [27], more effective treatments [28], and the impact of vaccination on morbidity and mortality [29, 30]. We accounted for this by comparing patients hospitalized with COVID-19 with patients hospitalized with other respiratory infections and found a similar temporal pattern of risk and observed the same pattern. In addition, undiagnosed or undocumented SARS-CoV-2 infections in the control cohorts undoubtedly increased during the pandemic, which could produce a decrease in HRs if COVID-19 itself is indeed a risk for subsequent anxiety and depression. Our study partially accounted for this by excluding positive antibody test results for SARS-CoV-2 before December 11, 2020, within the control cohorts to capture natural infection before vaccines were introduced. Additionally, if increasing undetected COVID-19 infections occurred in the control cohort and such infection was driving the outcome, we would expect to see an associated rise in the proportion of control patients who developed a depressive or anxiety disorder, which was not the case. Another explanation we considered was bias of ascertainment, or the possibility that COVID-19 patients received more medical follow-up and thus more opportunities for a mood or anxiety disorder to be detected, leading to elevated HRs. Indeed, COVID-19 patients had a greater number of follow-up visits on average throughout the study period, even when the HR was not significantly elevated. If increased follow-up among COVID-19 patients was responsible for elevated HRs, we would expect excess hazard to be present throughout the entire study period. Additionally, analyses comparing COVID-19 patients to bone fracture patients, a group that also requires follow-up, revealed similar temporal patterns.

Our results on risk stratified by age show a consistent elevation in older patients. While higher rates of anxiety and depressive symptoms have been reported among younger adults during the pandemic [8, 9, 31, 32] and were emphasized in the lay press, our study found older adults to be at increased absolute risk, contributing to a growing body of literature pointing to inconsistencies in the reported prevalence of mood and anxiety disorders in the elderly [33, 34]. Many factors could account for this excess risk of anxiety and depression in the elderly during the pandemic. The risk of death or hospitalization following COVID-19 was greater in the elderly even after vaccines and antiviral drugs became available, since breakthrough infections and incomplete treatment responses were more common. Thus, fear may have persisted in older patients. Older adults also may have been disproportionately affected by social distancing guidelines, leading to increased social isolation, inadequate care at long-term care facilities, and loss of home care [35–37] Additionally, there were undoubtedly greater disruptions in social networks of older individuals as peers and loved ones passed away. All these considerations might contribute to the increased risk for anxiety and depression among older adults. The elderly may also have had more severe COVID-19 infections and/or had more underlying medical conditions that produced contact with the health care system, presenting greater opportunity for mental health issues to be diagnosed and treated. While this has been largely accounted for by matching for medical risk factors for severe COVID-19 disease and critical care services as well as conducting sensitivity analyses on hospitalized patients, it is still possible that this bias accounts for a portion of our results.

Our results stratified by sex support reports of higher depressive and anxiety disorders in females [38, 39]. Women traditionally assume an excess of the caregiving burden, which increased during the pandemic as schools closed and home help was unavailable. Especially early in the pandemic, job and financial security were reduced, and domestic violence increased during lockdown. Moreover, men may be more reluctant to seek help [40]. Our study found no consistent difference in risk after COVID-19 or other RTI when stratified by race; a slight but significant reduction in hazard was evident when comparing Black patients to White patients, although previous studies indicated that racial minorities experienced greater declines in mental health during the pandemic due to factors such as decreased access to mental health services, higher rates of job loss, and collective trauma from instances of violence in communities of color [41–43]. It is possible that mental distress among communities of color did not lead to physician contact to the same extent that as in white communities, resulting in fewer diagnoses in the electronic health record [43].

Limitations of our study include our inability to determine causality due to its retrospective, observational design. In addition, despite the very large number of electronic records included in the study, sample sizes were too small to properly evaluate racial and intersectional subgroups that might be predicted to have special vulnerability, such as women of childbearing age or women of color. Our study does not cover patients who did not have contact with the health care system, nor is the population necessarily representative of the entire US population. Moreover, EHR data may not have captured all COVID-19 infections, especially later in the pandemic as home testing became more prevalent, and vaccination status is likely underreported in the TriNetX Analytics Platform as vaccine availability expanded outside of the health systems. These potential omissions may have impacted propensity score matching and precluded testing the impact of vaccination on mental health outcomes. It is important to recognize that recurrence of depressive or anxiety disorders, especially if prior episodes were remote, is difficult to assess in the electronic health record but could represent an important risk factor for new episodes. In fact, when both new and recurrent prescriptions for antidepressants and anxiolytics were analyzed, the number of patients doubled compared to first-time prescriptions. Other than recurrent major depressive disorder (F33), we did not measure the association between COVID-19 and individual *ICD-10* codes within the depressive and anxiety disorder subgroups. However, we did attempt to capture exacerbations of depressive and anxiety disorders by assessing prescriptions among patients who had not had prescriptions for these medications during the previous year. Anxiolytic drugs may have been prescribed for problems other than anxiety, such as sleep aids, and antidepressants may have been prescribed for indications other than depression, such as adjunctive pain therapy. However, the consistency of findings using the ICD-10 diagnostic categories and the pharmacologic interventions used most to treat them suggest that they are largely concordant measures for similar medical issues. Finally, although other RTIs and bone fractures were chosen as control health events to compare with COVID-19 infection, these diagnoses are not necessarily free of psychiatric risk. Both could also be linked to psychiatric sequelae through physiologic mechanisms, such as causing systemic inflammation that impacts the brain, or psychological mechanisms, such as limiting mobility and quality of life.

Overall, our findings, derived from a very large population, suggest that while a COVID-19 diagnosis was associated with elevated risk of depressive and anxiety disorders compared to control health events at the start of the pandemic, this risk decreased or was only mildly elevated after the first three months of the pandemic. It is likely that these temporal trends can be largely explained by the social context of the pandemic rather than, or in addition to, pathophysiologic mechanisms. Moreover, in our population, elderly patients were at particular risk throughout the duration of the pandemic. Recent USPSTF guidelines recommend screening for adults over 65 years old, but with only a “B” level of evidence for depression and insufficient evidence for anxiety [44, 45]. Our results indicate that more work is needed to improve the utility of screening instruments for older patients, especially since major public health challenges, ongoing, renewed, or new, are likely to continue. Additional research that captures patients without regular contact with the health system and patients from other racial and ethnic populations and in special communities such as the LGBTQ+ community is necessary to better understand the association between COVID-19 infection and mental health disorders and to identify those that may require continued screening.

### Supplementary information

Supplemental Material

## Data Availability

The data analyzed in this paper cannot be made available because we used a cloud-based database and cannot download the data set. This database is constantly being upgraded with new information, so the actual data from which the analysis was done will not be available at a subsequent time. That is why we indicate when the database was accessed, which specific data set was used, and the specifications for each cohort and analysis (either in the supplemental materials or in the main paper). Using these parameters, the study can then be repeated in the updated data set. The EMR data is deidentified and so individual data cannot be made available to us or anyone else.

## References

[1] World Health Organization. Mental Health and COVID-19: Early evidence of the pandemic’s impact: Scientific brief, 2 March 2022. https://www.who.int/publications/i/item/WHO-2019-nCoV-Sci_Brief-Mental_health-2022.1. Accessed 11 Mar 2023.

[2] Vahratian A, Blumberg SJ, Terlizzi EP, Schiller JS (2021). Symptoms of anxiety or depressive disorder and use of mental health care among adults during the COVID-19 pandemic—United States, August 2020–February 2021. MMWR Morb Mortal Wkly Rep..

[3] Santomauro DF, Mantilla Herrera AM, Shadid J, Zheng P, Ashbaugh C, Pigottt DM (2021). Global prevalence and burden of depressive and anxiety disorders in 204 countries and territories in 2020 due to the COVID-19 pandemic. Lancet..

[4] Taquet M, Luciano S, Geddes JR, Harrison PJ (2021). Bidirectional associations between COVID-19 and psychiatric disorder: retrospective cohort studies of 62 354 COVID-19 cases in the USA. Lancet Psychiatry..

[5] Taquet M, Geddes JR, Husain M, Luciano S, Harrison PJ (2021). 6-month neurological and psychiatric outcomes in 236 379 survivors of COVID-19: a retrospective cohort study using electronic health records. Lancet Psychiatry..

[6] Xie Y, Xu E, Al-Aly Z. Risks of mental health outcomes in people with COVID-19: cohort study. BMJ. 2022;376. 10.1136/bmj-2021-068993. 10.1136/bmj-2021-068993 PMC8847881 35172971

[7] Robinson E, Sutin AR, Daly M, Jones A (2022). A systematic review and meta-analysis of longitudinal cohort studies comparing mental health before versus during the COVID-19 pandemic in 2020. J Affect Disord..

[8] Shuster A, O’Brien M, Luo Y, Berner LA, Perl O, Heflin M (2021). Emotional adaptation during a crisis: decline in anxiety and depression after the initial weeks of COVID-19 in the United States. Transl Psychiatry..

[9] Daly M, Robinson E (2021). Psychological distress and adaptation to the COVID-19 crisis in the United States. J Psychiatr Res..

[10] https://www.cdc.gov/coronavirus/2019-ncov/covid-data/investigations-discovery/hospitalization-death-by-age.html. Accessed 26 Jun 2023.

[11] Gomez JMD, Du-Fay-de-Lavallaz JM, Fugar S, Sarau J, Simmons JA, Clark B (2021). Sex Differences in COVID-19 Hospitalization and Mortality. J Women’s Health (Larchmt)..

[12] https://www.cdc.gov/coronavirus/2019-ncov/covid-data/investigations-discovery/hospitalization-death-by-race-ethnicity.html. Accessed 26 Jun 2023.

[13] Centers for Disease Control and Prevention. Underlying medical conditions associated with higher risk for severe COVID-19: Information for healthcare professionals. https://www.cdc.gov/coronavirus/2019-ncov/hcp/clinical-care/underlyingconditions.html#print. Accessed 4 Mar 2023. 34009770

[14] Fancourt D, Steptoe A, Bu F (2021). Trajectories of anxiety and depressive symptoms during enforced isolation due to COVID-19 in England: a longitudinal observational study. Lancet Psychiatry..

[15] Olaker VR, Kendall EK, Wang CX, Parran TV, Terebuh P, Kaleber DC (2023). Association of Recent SARS-CoV-2 Infection With New-Onset Alcohol Use Disorder, January 2020 Through January 2022. JAMA Netw Open..

[16] Centers for Disease Control and Prevention. CDC updates and shortens recommended isolation and quarantine period for general population. https://www.cdc.gov/media/releases/2021/s1227-isolation-quarantine-guidance.html. Accessed 4 Mar 2023.

[17] Perez-Arce F, Angrisani M, Bennett D, Darling J, Kapteyn A, Thomas K (2021). COVID-19 vaccines and mental distress. PLoS One..

[18] Chen S, Aruldass AR, Cardinal RN (2022). Mental health outcomes after SARS-CoV-2 vaccination in the United States: a national cross-sectional study. J Affect Disord..

[19] Anderson J, Fingerhut H. COVID anxiety rising amid delta surge, AP-NORC poll finds. AP News. Published 2021. https://apnews.com/article/lifestyle-business-health-travel-coronavirus-pandemic-27bf20514cd3da917c54bf71a41f2e8e. Accessed 4 Mar 2023.

[20] Galatzer-Levy IR, Huang SH, Bonanno GA (2018). Trajectories of resilience and dysfunction following potential trauma: a review and statistical evaluation. Clin Psychol Rev..

[21] Daly M, Robinson E (2022). Depression and anxiety during COVID-19. Lancet..

[22] Chen S, Bonanno GA (2020). Psychological adjustment during the global outbreak of COVID-19: a resilience perspective. Psychol Trauma..

[23] Lancee WJ, Maunder RG, Goldbloom DS (2008). Prevalence of psychiatric disorders among Toronto hospital workers one to two years after the SARS outbreak. Psychiatr Serv..

[24] Bonanno GA, Ho SM, Chan JC, Kwong RSY, Cheung CKY, Wong CPY (2008). Psychological resilience and dysfunction among hospitalized survivors of the SARS epidemic in Hong Kong: a latent class approach. Health Psychol..

[25] Thaweethai T, Jolley SE, Karlson EW, Levitan EB, Levy B, McComsey GA (2023). Development of a definition of postacute sequelae of SARS-CoV-2 infection. JAMA..

[26] Callard F, Perego E (2021). How and why patients made long Covid. Soc Sci Med..

[27] Markov PV, Ghafari M, Beer M, Lythgoe K, Simmonds P, Stilianakis NI (2023). The evolution of SARS-CoV-2. Nat Rev Microbiol.

[28] Chokkalingam AP, Hayden J, Goldman JD, Li H, Asubonteng J, Mozaffri E (2022). Association of remdesivir treatment with mortality among hospitalized adults with COVID-19 in the United States. JAMA Netw Open.

[29] Notarte KI, Catahay JA, Velasco JV, Pastrana A, Ver AT, Pangilian FC (2022). Impact of COVID-19 vaccination on the risk of developing long-COVID and on existing long-COVID symptoms: a systematic review. EClinicalMedicine.

[30] Moghadas SM, Vilches TN, Zhang K, Wells CR, Shoukat A, Singer BH (2021). The impact of vaccination on coronavirus disease 2019 (COVID-19) outbreaks in the United States. Clin Infect Dis.

[31] Varma P, Junge M, Meaklim H, Jackson ML (2021). Younger people are more vulnerable to stress, anxiety and depression during COVID-19 pandemic: a global cross-sectional survey. Prog Neuro-Psychopharmacol Biol Psychiatry.

[32] Zhu C, Zhang T, Li Q, Chen X, Wang K (2023). Depression and anxiety during the COVID-19 pandemic: epidemiology, mechanism, and treatment. Neurosci Bull.

[33] Andreas S, Schulz H, Volkert J, Dehoust M, Sehner S, Suling A (2017). Prevalence of mental disorders in elderly people: the European MentDis_ICF65 study. Br J Psychiatry..

[34] Fiske A, Wetherell JL, Gatz M (2009). Depression in older adults. Annu Rev Clin Psychol..

[35] Andreescu C, Lenze E, Lavretsky H (2023). Is anxiety in late life an uncharted territory?—questioning the USPSTF draft recommendation statement for anxiety screening in older adults. JAMA Psychiatry..

[36] Hadjistavropoulos T, Asmundson GJG (2022). COVID stress in older adults: considerations during the Omicron wave and beyond. J Anxiety Disord..

[37] United Nations. Policy Brief: The Impact of COVID-19 on older persons. https://unsdg.un.org/sites/default/files/2020-05/Policy-Brief-The-Impact-of-COVID-19-on-Older-Persons.pdf. Accessed 4 Mar 2023.

[38] Metin A, Erbiçer ES, Şen S, Çetinkaya A (2022). Gender and COVID-19 related fear and anxiety: a meta-analysis. J Affect Disord..

[39] Özdin S, Bayrak Özdin Ş (2020). Levels and predictors of anxiety, depression and health anxiety during COVID-19 pandemic in Turkish society: The importance of gender. Int J Soc Psychiatry..

[40] Addis M, Mahalik J (2003). Men, masculinity, and the contexts of help seeking. Am Psychol..

[41] Thomeer MB, Moody MD, Yahirun J (2023). Racial and ethnic disparities in mental health and mental health care during the COVID-19 pandemic. J Racial Ethn Health Disparities..

[42] Nguyen LH, Anyane-Yeboa A, Klaser K, Merina A, Drew DA, Ma W (2022). The mental health burden of racial and ethnic minorities during the COVID-19 pandemic. PLOS One..

[43] Panchal N, Saunders H, Ndugga N. Five Key Findings on Mental Health and Substance Use Disorders by Race/Ethnicity. KFF. Published 2022. https://www.kff.org/racial-equity-and-health-policy/issue-brief/five-key-findings-on-mental-health-and-substance-use-disorders-by-race-ethnicity/. Accessed 4 Mar 2023.

[44] https://www.uspreventiveservicestaskforce.org/uspstf/recommendation/screening-depression-suicide-risk-adults. Accessed 22 Jun 2023.

[45] https://www.uspreventiveservicestaskforce.org/uspstf/recommendation/anxiety-adults-screening. Accessed 22 Jun 2023.

