# Supplementary material for: Risk for diagnosis or treatment of mood or anxiety disorders in adults after SARS-CoV-2 infection, 2020–2022
Source: Mol Psychiatry. 2024 Jan 18;29(5):1350–60. doi: 10.1038/s41380-024-02414-x (PMC11189805; doi:10.1038/s41380-024-02414-x)
Supplement: Supplementary file 1 — Supplemental Material [file 41380_2024_2414_MOESM1_ESM.docx]

**Supplementary Online Content**

**eMethods.**

**eFigure 1.** Risk of Recurrent Major Depressive Disorder Diagnosis After COVID-19 Diagnosis vs. Other Respiratory Infection Diagnosis

**eFigure 2.** Risk of New Depressive or Anxiety Disorder Diagnosis After COVID-19 Diagnosis Including Positive Lab Test in Cohort Definition vs. Other Respiratory Infection Diagnosis

**eFigure 3.** Risk of New Depressive or Anxiety Disorder Diagnosis After COVID-19 Diagnosis vs. Bone Fracture Diagnosis

**eFigure 4.** Risk of New Depressive Disorder Diagnosis After COVID-19 Diagnosis vs. Other Respiratory Infection Diagnosis

**eFigure 5.** Risk of New Anxiety Disorder Diagnosis After COVID-19 Diagnosis vs. Other Respiratory Infection Diagnosis

**eFigure 6.** Risk of New Diagnosis or Prescription After COVID-19 Diagnosis vs. Other Respiratory Infection Diagnosis by Sex

**eFigure 7.** Risk of New Diagnosis or Prescription After COVID-19 Diagnosis vs. Other Respiratory Infection Diagnosis by Race

**eFigure 8.** Risk of New Depressive or Anxiety Disorder Diagnosis After COVID-19 Diagnosis vs Other Respiratory Infection Diagnosis, 6 Month Follow-up

**eFigure 9.** Risk of New Depressive or Anxiety Disorder Diagnosis After COVID-19 Diagnosis vs Other Respiratory Infection Diagnosis Among Hospitalized Patients

**eTable 1. Baseline Characteristics for Block 1 Before and After Matching**

**eTable 2. Baseline Characteristics for Block 2 Before and After Matching**

**eTable 3. Baseline Characteristics for Block 3 Before and After Matching**

**eTable 4. Baseline Characteristics for Block 4 Before and After Matching**

**eTable 5. Baseline Characteristics for Block 5 Before and After Matching**

**eTable 6. Baseline Characteristics for Block 6 Before and After Matching**

**eTable 7. Baseline Characteristics for Block 7 Before and After Matching**

**eTable 8. Baseline Characteristics for Block 8 Before and After Matching**

**eTable 9. Baseline Characteristics for Block 9 Before and After Matching**

**eMethods**

**TriNetX Analytics Platform description**

The data used in this study was accessed between February 6 and March 17, 2023 from the Research USA No Date Shift Network. This resource provides access to electronic medical records (diagnoses, procedures, medications, laboratory values, genomic information) from over 92 million patients from 34 healthcare organizations, which is de-identified per criteria from the Health Insurance Portability and Accountability Act (HIPAA), Section §164.514(a) of the HIPAA Privacy Rule. MetroHealth System, Cleveland, Ohio, IRB has determined any research using TriNetX, is not Human Subject Research and therefore exempt from IRB review.

The Research USA No Date Shift Network platform de-identifies and aggregates electronic health record (EHR) data from 34 contributing healthcare systems, most of which are large academic medical institutions with both inpatient and outpatient facilities at multiple locations across the US. Patient EHR data includes information from hospitals, primary care, and specialty treatment providers, covering diverse geographic locations, age groups, racial and ethnic groups, income levels and insurance types including various commercial insurances, governmental insurance (Medicare and Medicaid), self-pay/uninsured, worker compensation insurance, military/VA insurance among others. Race and ethnicity data in TriNetX is derived from self-reports in the clinical EHR systems, which is then mapped to the following categories: (1) Race: Asian, American Indian or Alaskan Native, Black or African American, Native Hawaiian or Other Pacific Islander, White, Unknown race; and (2) Ethnicity: Hispanic or Latino, Not Hispanic or Latino, Unknown Ethnicity.

**Cohort definitions**

The primary COVID-19 cohort was defined by the presence of an encounter diagnostic code for COVID-19 (U07.1). Since this code did not exist until April 1, 2020, additional diagnostic codes were included for Block 1 (January 1 to April 1, 2020):

U07.1: COVID-19

U07.2: COVID-19, virus not identified (WHO)

B34.2: Coronavirus infection, unspecified

B97.29: Other coronavirus as the cause of diseases classified elsewhere

J12.81: Pneumonia due to SARS-associated coronavirus

One diagnostic code for other specified viral infection (079.89) was also excluded for Block 1 as recommended by TriNetX. Patients with an ICD-10 code for COVID-19 or a positive RNA test result for SARS-CoV-2 3 months prior to the index event were excluded.

The other respiratory tract infection cohort was defined by the presence of an encounter diagnostic code for any of the following diagnoses, as adapted by Taquet et al.:

J00: Acute nasopharyngitis [common cold]

J01: Acute sinusitis

J02: Acute pharyngitis

J03: Acute tonsillitis

J04: Acute laryngitis and tracheitis

J05: Acute obstructive laryngitis [croup] and epiglottitis

J06: Acute upper respiratory infections of multiple and unspecified sites

J09: Influenza due to certain identified influenza virus

J10: Influenza due to other identified influenza virus

J11: Influenza due to unidentified influenza virus

J12: Viral pneumonia, not elsewhere classified

J13: Pneumonia due to Streptococcus pneumoniae

J14: Pneumonia due to Hemophilus influenzae

J15: Bacterial pneumonia, not elsewhere classified

J16: Pneumonia due to other infectious organisms, not elsewhere classified

J17: Pneumonia in diseases classified elsewhere

J18: Pneumonia, unspecified organism

J20: Acute bronchitis

J21: Acute bronchiolitis

J22: Unspecified acute lower respiratory infection

The following diagnostic codes were excluded from January 1 to March 31, 2020 to exclude patients who contracted COVID-19 in the beginning of the pandemic before the U07.1 diagnostic code was created on April 1, 2020.

B34.2: Coronavirus infection, unspecified

B97.29: Other coronavirus as the cause of diseases classified elsewhere

J12.81: Pneumonia due to SARS-associated coronavirus

The following diagnostic codes and positive RNA test results for SARS-CoV-2 were excluded at any point in time:

U07.1: COVID-19

U07.2: COVID-19, virus not identified (WHO)

J12.82: Pneumonia due to COVID-19

9088: SARS coronavirus 2 and related RNA [Presence] (labResult: Positive)

94558-4: SARS coronavirus 2 Ag [Presence] in Respiratory specimen by Rapid immunoassay (labResult: Positive)

94311-8: SARS coronavirus 2 N gene [Cycle Threshold #] in Unspecified specimen by Nucleic acid amplification using CDC primer-probe set N1 (at most 40.00 units)

95522-9: SARS-CoV-2 (COVID-19) N gene [Log #/volume] (viral load) in Respiratory specimen by NAA with probe detection (at least 0.80 {Log_copies}/mL)

97097-0: SARS-CoV-2 (COVID-19) Ag [Presence] in Upper respiratory specimen by Rapid immunoassay (labResult: Positive)

95209-3: SARS coronavirus+SARS coronavirus 2 Ag [Presence] in Respiratory specimen by Rapid immunoassay (labResult: Positive)

96603-6: SARS-CoV-2 (COVID-19) S protein RBD neutralizing antibody [Presence] in Serum or Plasma by Immunoassay (labResult: Positive)

96119-3: SARS-CoV-2 (COVID-19) Ag [Presence] in Upper respiratory specimen by Immunoassay (labResult: Positive)

The following positive antibody test results for SARS-CoV-2 were excluded anytime before December 11, 2020 to exclude patients with natural COVID-19 infection before vaccines were introduced, after which the presence of antibodies due to natural infection or receiving the vaccine would be indistinguishable:

9089: SARS coronavirus 2 IgG IgM Ab [Presence] in Serum or Plasma (labResult: Positive)

94505-5: SARS-CoV-2 (COVID-19) IgG Ab [Units/volume] in Serum or Plasma by Immunoassay (at least 0.10 [arb'U]/mL)

94506-3: SARS-CoV-2 (COVID-19) IgM Ab [Units/volume] in Serum or Plasma by Immunoassay (at least 0.10 [arb'U]/mL)

94562-6: SARS-CoV-2 (COVID-19) IgA Ab [Presence] in Serum or Plasma by Immunoassay (labResult: Positive)

94762-2: SARS-CoV-2 (COVID-19) Ab [Presence] in Serum or Plasma by Immunoassay (labResult: Positive)

94769-7: SARS-CoV-2 (COVID-19) Ab [Units/volume] in Serum or Plasma by Immunoassay (at least 0.10 [IU]/mL)

**Full covariate list**

98 covariates were matched for analyses. Cohorts were matched for demographics including: current age, age at index, female, male, unknown gender, American Indian or Alaska Native, Asian, Black or African American, Native Hawaiian or other Pacific Islander, unknown race, White, Hispanic or Latinx, not Hispanic or Latinx, and unknown ethnicity.

Cohorts were matched for physiologic risk factors for severe COVID-19 illness, and this list was the same used by Taquet et al:

E66: Overweight and obesity

I10-I16: Hypertensive diseases

E10: Type 1 diabetes mellitus

E11: Type 2 diabetes mellitus

J40: Bronchitis, not specified as acute or chronic

J41: Simple and mucopurulent chronic bronchitis

J42: Unspecified chronic bronchitis

J43: Emphysema

J44: Other chronic obstructive pulmonary disease

J45: Asthma

J47: Bronchiectasis

I30-I15A: Other forms of heart disease

I12: Hypertensive chronic kidney disease

K72: Hepatic failure, not elsewhere classified

K73: Chronic hepatitis, not elsewhere classified

K74: Fibrosis and cirrhosis of liver

K76.0: Fatty (change of) liver, not elsewhere classified

K76.1: Chronic passive congestion of liver

K76.6: Portal hypertension

K76.8: Other specified diseases of liver

I63: Cerebral infarction

F01: Vascular dementia

F02: Dementia in other diseases classified elsewhere

F03: Unspecified dementia

G30: Alzheimer's disease

G31.0: Frontotemporal dementia

G31.83: Dementia with Lewy bodies

C00-D49: Neoplasms

C81-C96: Malignant neoplasms of lymphoid, hematopoietic and related tissue

M05: Rheumatoid arthritis with rheumatoid factor

M06: Other rheumatoid arthritis

M32: Systemic lupus erythematosus (SLE)

L40: Psoriasis

D80-D89: Certain disorders involving the immune mechanism

1008098: Renal Transplantation Procedures

1007811: Liver Transplantation Procedures

BMI, categorized into 3 groups: <25kg/m2, 25-30kg/m2, >30kg/m2

Blood pressure, systolic, categorized into 3 groups: <140 mm[Hg], 140-160 mm[Hg], >160 mm[Hg]

Blood pressure, diastolic, categorized into 3 groups: <90 mm[Hg], 90-100 mm[Hg], >100 mmHg

Cohorts were matched for psychiatric risk factors for severe COVID-19 illness, including substance use disorders and psychotic disorders:

F10: Alcohol related disorders

F11: Opioid related disorders

F12: Cannabis related disorders

F13: Sedative, hypnotic, or anxiolytic related disorders

F14: Cocaine related disorders

F15: Other stimulant related disorders

F16: Hallucinogen related disorders

F17: Nicotine dependence

F18: Inhalant related disorders

F19: Other psychoactive substance related disorders

F20: Schizophrenia

F21: Schizotypal disorder

F22: Delusional disorders

F23: Brief psychotic disorder

F24: Shared psychotic disorder

F25: Schizoaffective disorders

F28: Other psychotic disorder not due to a substance or known physiological condition

F29: Unspecified psychosis not due to a substance or known physiological condition

Cohorts were matched for mental health history and socioeconomic determinants of health:

Z55-Z65: Persons with potential health hazards related to socioeconomic and psychosocial circumstances

Z81: Family history of mental and behavioral disorders

Z86.59: Personal history of other mental and behavioral disorders

Cohorts were matched for prior hospitalization:

1013659: Hospital Inpatient Services

1013661: New or Established Patient Initial Hospital Inpatient Care Services

1013729: Critical Care Services

IMP: Visit: Inpatient Encounter

NONAC: Visit: Inpatient Non-acute

SS: Visit: Short Stay

Cohorts were matched for COVID-19 vaccination status:

91300: Severe acute respiratory syndrome coronavirus 2 (SARS-CoV-2) (Coronavirus disease [COVID-19]) vaccine, mRNA-LNP, spike protein, preservative free, 30 mcg/0.3mL dosage, diluent reconstituted, for intramuscular use

0001A: Immunization administration by intramuscular injection of severe acute respiratory syndrome coronavirus 2 (SARS-CoV-2) (Coronavirus disease [COVID-19]) vaccine, mRNA-LNP, spike protein, preservative free, 30 mcg/0.3mL dosage, diluent reconstituted; first dose

0002A: Immunization administration by intramuscular injection of severe acute respiratory syndrome coronavirus 2 (SARS-CoV-2) (Coronavirus disease [COVID-19]) vaccine, mRNA-LNP, spike protein, preservative free, 30 mcg/0.3mL dosage, diluent reconstituted; second dose

91301: Severe acute respiratory syndrome coronavirus 2 (SARS-CoV-2) (Coronavirus disease [COVID-19]) vaccine, mRNA-LNP, spike protein, preservative free, 100 mcg/0.5mL dosage, for intramuscular use

0011A: Immunization administration by intramuscular injection of severe acute respiratory syndrome coronavirus 2 (SARS-CoV-2) (Coronavirus disease [COVID-19]) vaccine, mRNA-LNP, spike protein, preservative free, 100 mcg/0.5mL dosage; first dose

0012A: Immunization administration by intramuscular injection of severe acute respiratory syndrome coronavirus 2 (SARS-CoV-2) (Coronavirus disease [COVID-19]) vaccine, mRNA-LNP, spike protein, preservative free, 100 mcg/0.5mL dosage; second dose

91302: Severe acute respiratory syndrome coronavirus 2 (SARS-CoV-2) (coronavirus disease [COVID-19]) vaccine, DNA, spike protein, chimpanzee adenovirus Oxford 1 (ChAdOx1) vector, preservative free, 5x10^10 viral particles/0.5mL dosage, for intramuscular use

0021A: Immunization administration by intramuscular injection of severe acute respiratory syndrome coronavirus 2 (SARS-CoV-2) (coronavirus disease [COVID-19]) vaccine, DNA, spike protein, chimpanzee adenovirus Oxford 1 (ChAdOx1) vector, preservative free, 5x10^10 viral particles/0.5mL dosage; first dose

0022A: Immunization administration by intramuscular injection of severe acute respiratory syndrome coronavirus 2 (SARS-CoV-2) (coronavirus disease [COVID-19]) vaccine, DNA, spike protein, chimpanzee adenovirus Oxford 1 (ChAdOx1) vector, preservative free, 5x10^10 viral particles/0.5mL dosage; second dose

91303: Severe acute respiratory syndrome coronavirus 2 (SARS-CoV-2) (coronavirus disease [COVID-19]) vaccine, DNA, spike protein, adenovirus type 26 (Ad26) vector, preservative free, 5x10^10 viral particles/0.5mL dosage, for intramuscular use

0031A: Immunization administration by intramuscular injection of severe acute respiratory syndrome coronavirus 2 (SARS-CoV-2) (coronavirus disease [COVID-19]) vaccine, DNA, spike protein, adenovirus type 26 (Ad26) vector, preservative free, 5x10^10 viral particles/0.5mL dosage, single dose

XW013S6: Introduction of COVID-19 Vaccine Dose 1 into Subcutaneous Tissue, Percutaneous

Approach, New Technology Group 6

XW013T6: Introduction of COVID-19 Vaccine Dose 2 into Subcutaneous Tissue, Percutaneous

Approach, New Technology Group 6

XW013U6: Introduction of COVID-19 Vaccine into Subcutaneous Tissue, Percutaneous Approach, New Technology Group 6

XW023U6: Introduction of COVID-19 Vaccine into Muscle, Percutaneous Approach, New Technology Group 6

XW023T6: Introduction of COVID-19 Vaccine Dose 2 into Muscle, Percutaneous Approach, New Technology Group 6

XW023S6: Introduction of COVID-19 Vaccine Dose 1 into Muscle, Percutaneous Approach, New Technology Group 6

213: SARS-CoV-2 (COVID-19) Vaccine

**Details on statistical analysis**

The status of SARS-CoV-2 infection was based on the International Classification of Diseases (ICD-10) diagnosis of U07.1. The outcome measure of new depressive or anxiety disorder was determined by the presence of a new ICD-10 diagnosis of a depressive disorder (F30-F39) or anxiety disorder (F40-F48) as well as the presence of a first-time prescription for an antidepressant medication (VA:CN600 or ATC:N06A) or an anxiolytic medication (ATC:N05B).

The antidepressant outcome was defined by the following medication codes:

N06AA: Non-selective monoamine reuptake inhibitors

N06AB: Selective serotonin reuptake inhibitors

N06AF: Monoamine oxidase inhibitors, non-selective

N06AG: Monoamine oxidase A inhibitors

N06AX: Other antidepressants

CN601: Tricyclic antidepressants

CN602: Monoamine oxidase inhibitor antidepressants

CN609: Antidepressants, other

The anxiolytic outcome was defined by the following medication codes:

N05BA: Bendoziazepine derivatives

N05BB: Dephenylmethane derivatives

N05BC: Carbamates

N05BE: Azaspirodecanedione derivatives

The outcome measure of new or recurrent depressive or anxiety disorder was determined by the presence of any prescription for an antidepressant or anxiolytic medication up until 1 year before the index event. For stratified results, the composite outcome of either a new ICD-10 diagnosis of a depressive or anxiety disorder or a first-time antidepressant or anxiolytic prescription was used, and this risk was stratified by age (18-35, 36-49, 50-64, and ≥65 years old), sex (female and male), and race (Black and White). These results are shown in figure 6, 7, and 8. To compare the risk of depressive or anxiety disorders after COVID-19 infection between demographic groups, the composite outcome was used and COVID-19 patients of different demographic groups were compared to each other over the entire study period (January 1, 2020 to April 9, 2022). Adults ≥65 years old were compared to adults <65 years old (age groups 18-35 years, 36-49 years, and 50-64 years were condensed), females were compared to males, and Black patients were compared to White patients.

Each COVID-19 cohort was matched with a cohort of patients without SARS-CoV2 infection by the TriNetX built‐in propensity score matching function (1:1 matching using a nearest neighbor greedy matching algorithm with a caliper of 0.25 times the standard deviation). The risk of the outcome after the index event was then compared between the SARS-CoV-2 cohort and the other respiratory tract infection cohort using hazard ratios and 95% confidence intervals. Kaplan-Meier analysis was used to estimate the probability of clinical outcomes. Cox’s proportional hazards model was used to compare the two matched cohorts. The proportional hazard assumption was tested using the generalized Schoenfeld approach. In our main analyses, there was a violation of proportionality for the risk of a new diagnosis in Block 8 with p=0.0119, for the risk of a new prescription in Block 6 with p<0.0001, and for the risk of new and recurrent prescriptions in Block 2 with p=0.0135, Block 6 with p=0.0002, Block 7 with p=0.0104, and Block 8 with p=0.0002. The TriNetX Platform calculates the hazard ratios and associated confidence intervals, using R's Survival package v3.2-3. For generating hazard ratios, TriNetX sets robust=FALSE using the R survival package, but it does not take into account potential clustering of COVID-19 cases within the healthcare organizations or specific geolocations, a potential weakness or confounding factor in the analysis.

Additional analyses were done to test the robustness of the findings presented. Analyses were run with the outcome of an ICD-10 diagnosis of recurrent major depressive disorder (F33) to capture recurrent depression. The results yielded similar temporal patterns to the main analysis and are shown in figure 1.

Additional analyses were done where positive COVID-19 tests (using rollup lab code 9088 for any positive RNA test ever) were added to the COVID cohorts in addition to an ICD-10 diagnosis of COVID-19 (U07.1). These results yielded similar temporal patterns to the main analysis and are shown in figure 2.

Additional analyses were run that changed the index event of the control cohort to large bone fractures (adapted from Taquet et al.) rather than other respiratory tract infections. These results yielded similar temporal patterns to the main analysis and are shown in figure 3. The codes used for inclusion were any of the following:

S32: Fracture of lumbar spine and pelvis

S42: Fracture of shoulder and upper arm

S52: Fracture of forearm

F72: Fracture of femur

S82: Fracture of lower leg, including ankle

Additional analyses were run with the outcome of a new ICD-10 diagnosis of a depressive disorder (F30-F39) alone as well as with the outcome of a new ICD-10 diagnosis of an anxiety disorder (F40-F48) alone. These results yielded similar temporal patterns to the main analysis and are shown in figure 4 and 5.

Additional analyses were run where the follow-up period after an index event was extended from a 2 week to 3 month window to a 2 week to 6 month window to compare the risk of a depressive or anxiety disorder after a COVID-19 versus other RTI diagnosis. These results yielded similar temporal patterns to the main analysis and are shown in figure 8.

Additional analyses were run where the risk of a depressive or anxiety disorder after a COVID-19 versus other RTI diagnosis was compared in patients who had an inpatient encounter one month prior to one month after the index event. This time constraint was chosen in order to capture patients who were hospitalized as a result of their COVID-19 or other RTI diagnosis. These results yielded similar temporal patterns to the main analysis and are shown in figure 9.

**Baseline characteristics for all cohorts**

Table 1 in the article presented baseline characteristics for the Block 1 cohorts. This table was reduced to only show demographics and physiologic and psychiatric risk factors for severe COVID-19 illness. The full baseline characteristics tables for Blocks 1-9 are available shown in Tables 1-9.

**eFigure 1.**

**
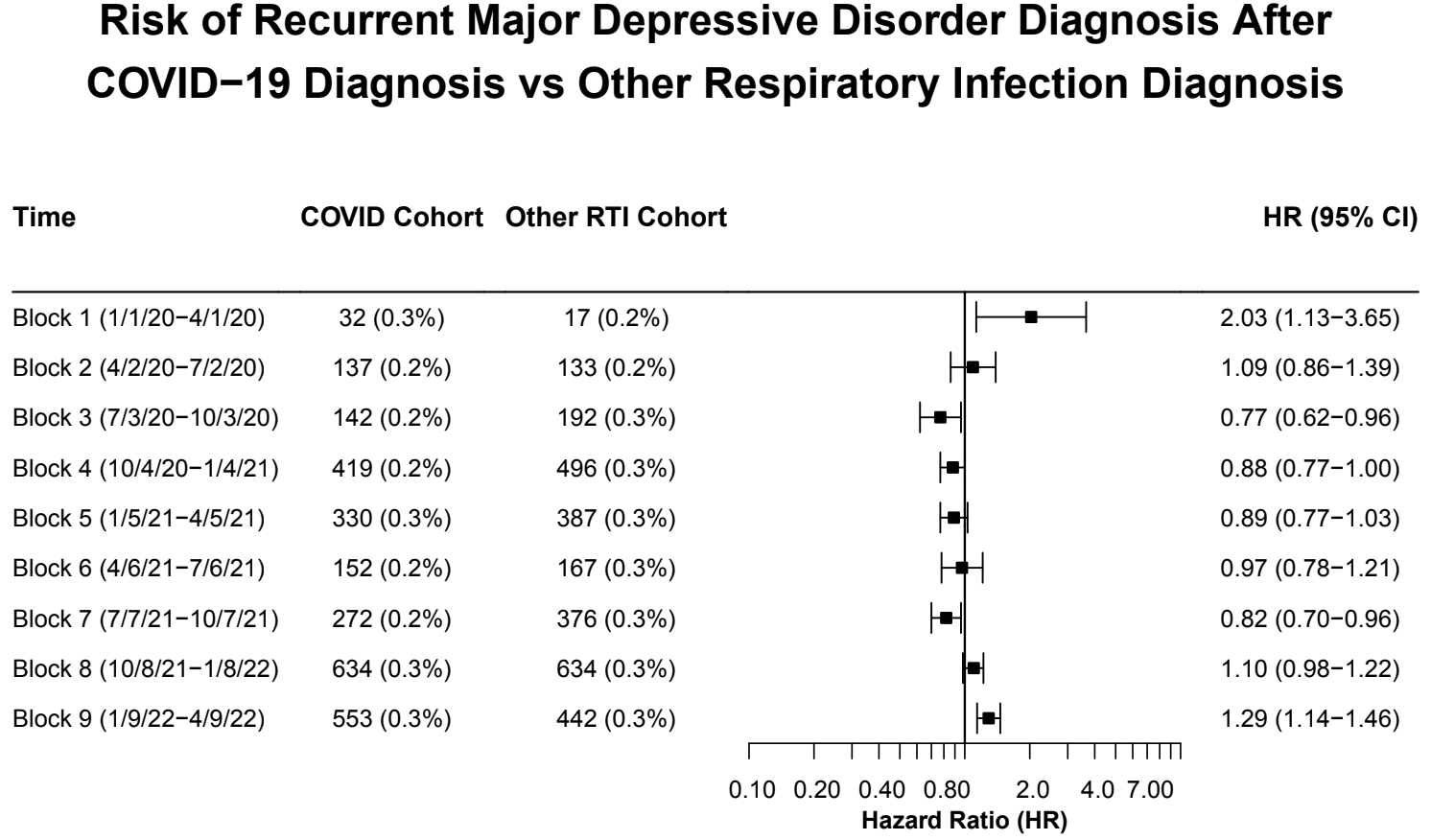
**

This plot shows the HRs and 95% CIs when COVID-19 cohorts were compared to other RTI cohorts for the outcome of an encounter diagnosis code for recurrent major depressive disorder (F33).

**eFigure 2.**

**
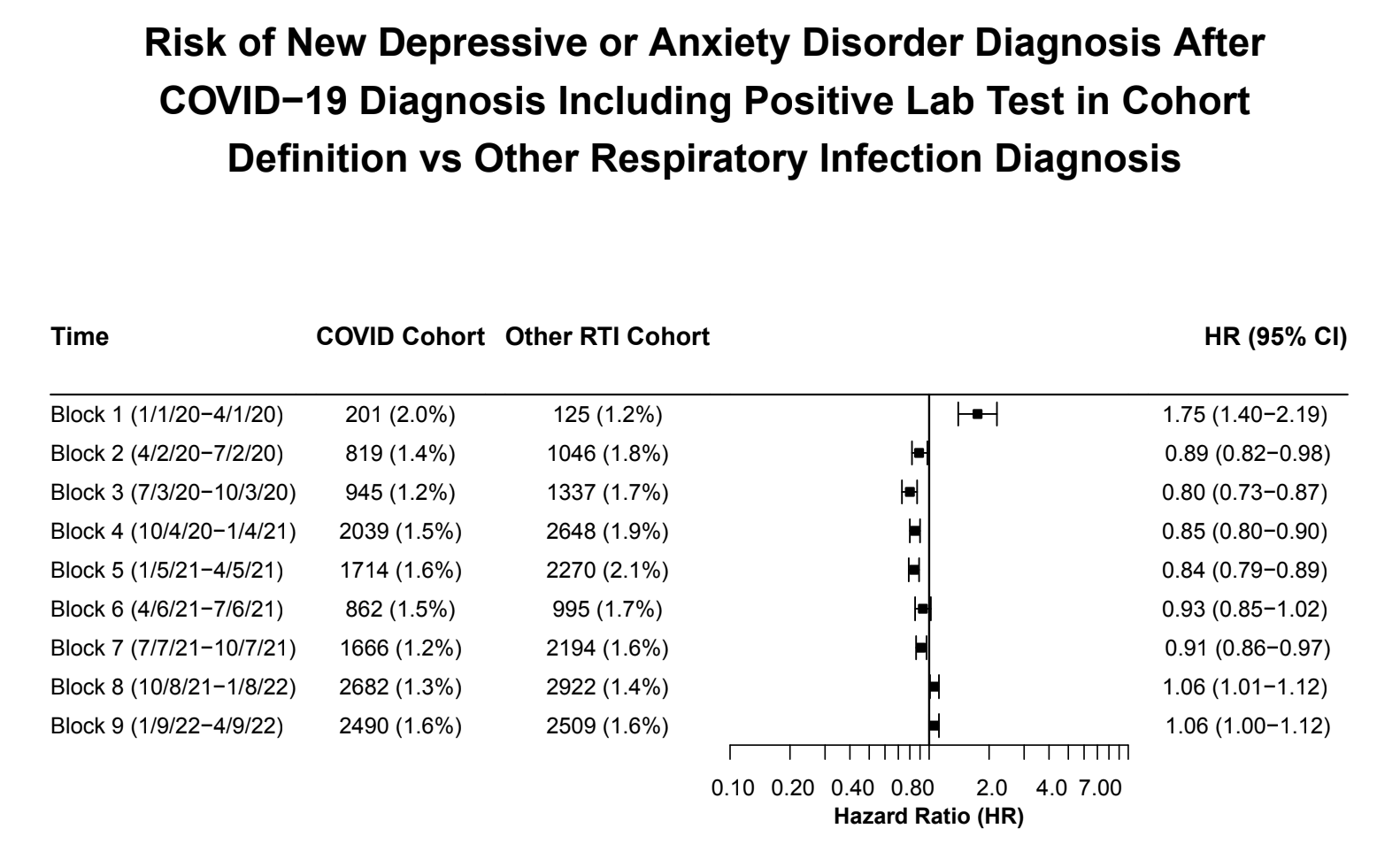
**

This plot shows the HRs and 95% CIs when COVID-19 cohorts defined by either the *ICD-10* code for COVID-19 (U07.1) or a positive RNA test result for SARS-CoV-2 were compared to other RTI cohorts for the outcome of a new depressive or anxiety disorder diagnosis.

**eFigure 3.**


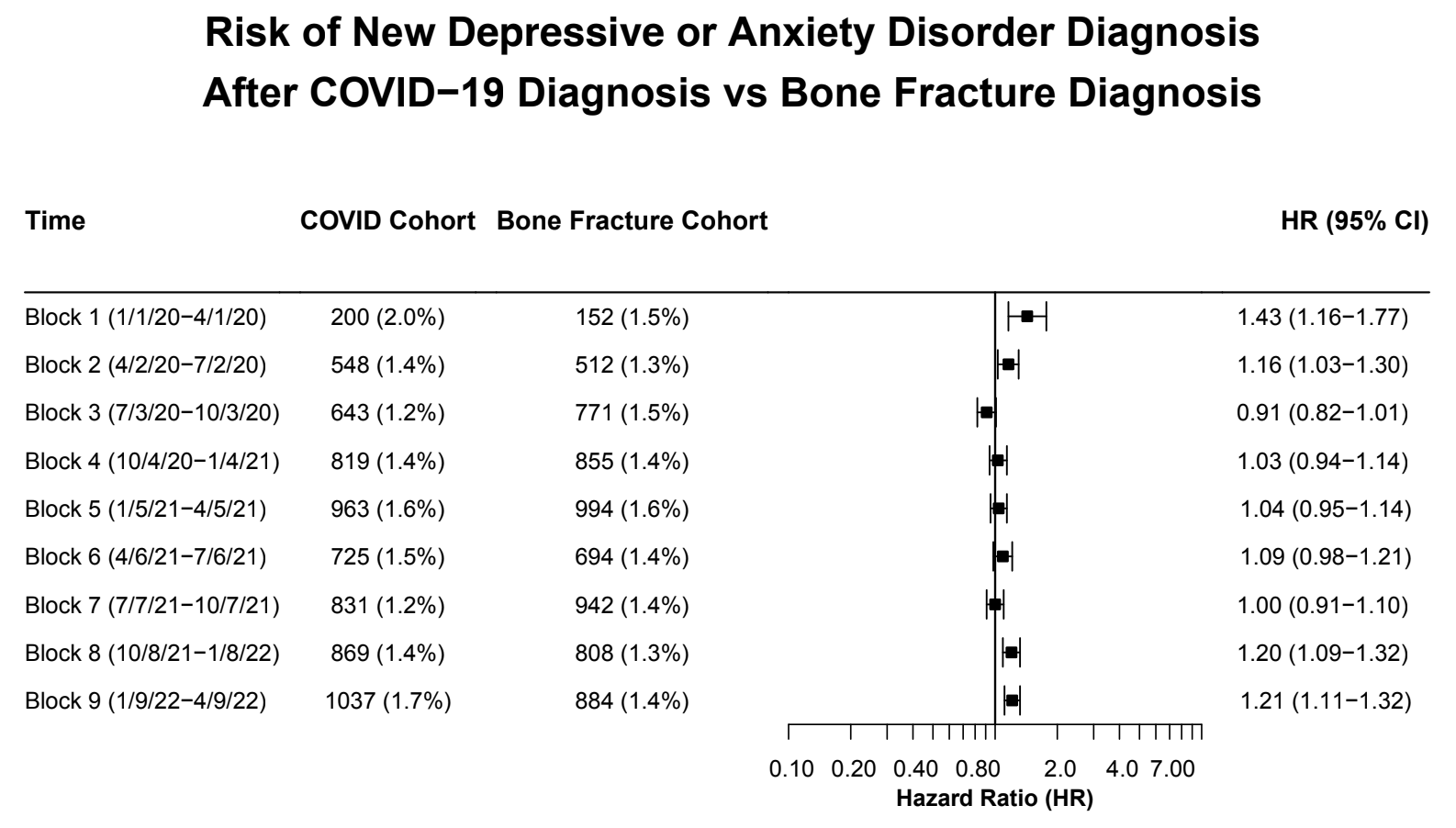


This plot shows the HRs and 95% CIs when COVID-19 cohorts were compared to control cohorts defined by encounter diagnosis codes for bone fracture, as adapted by Taquet et al, for the outcome of a new depressive or anxiety disorder diagnosis.

**eFigure 4.**


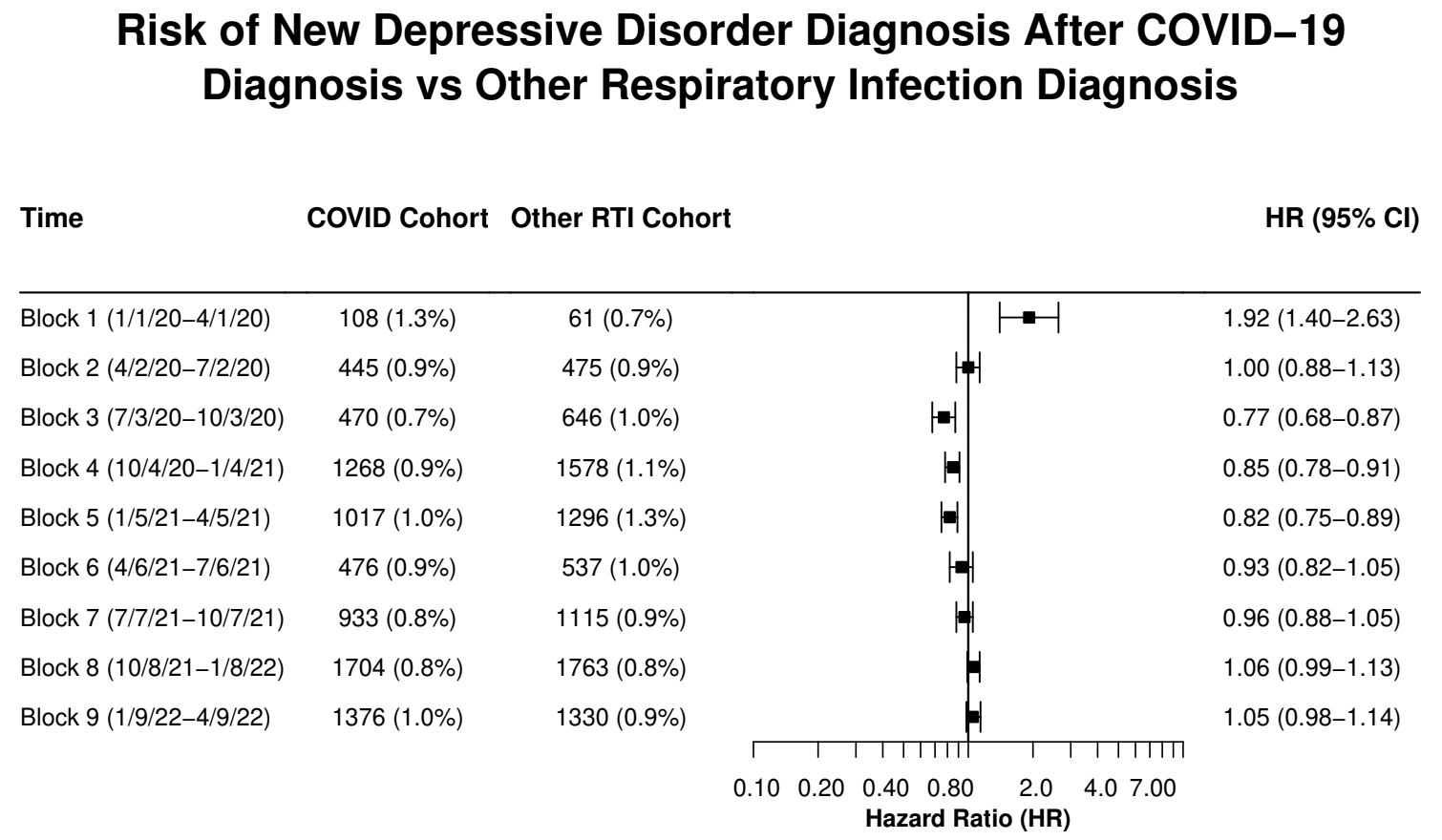


This plot shows the HRs and 95% CIs when COVID-19 cohorts were compared to other RTI cohorts for the outcome of a new depressive disorder diagnosis.

**eFigure 5.**


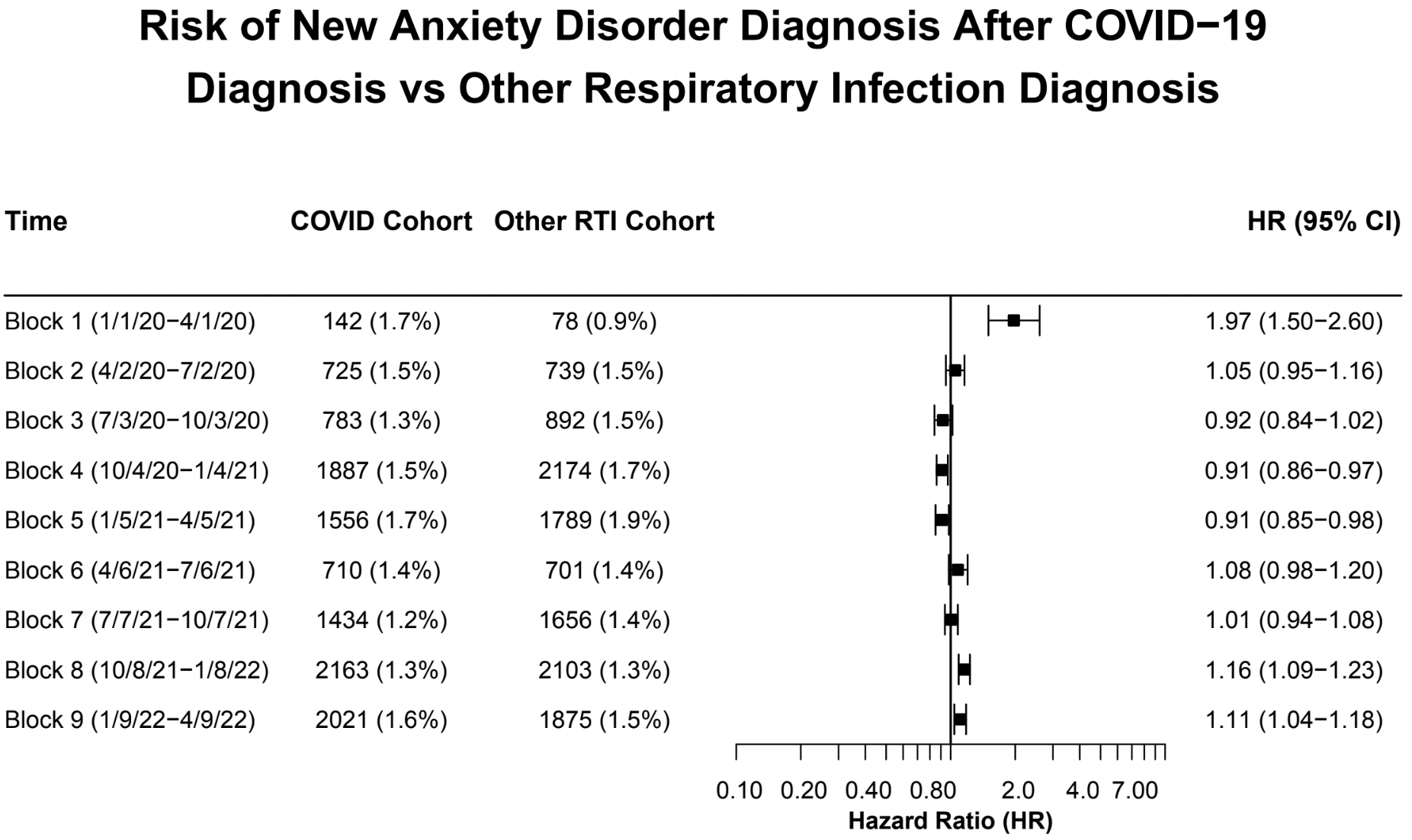


This plot shows the HRs and 95% CIs when COVID-19 cohorts were compared to other RTI cohorts for the outcome of a new anxiety disorder diagnosis.

.

**eFigure 6.**


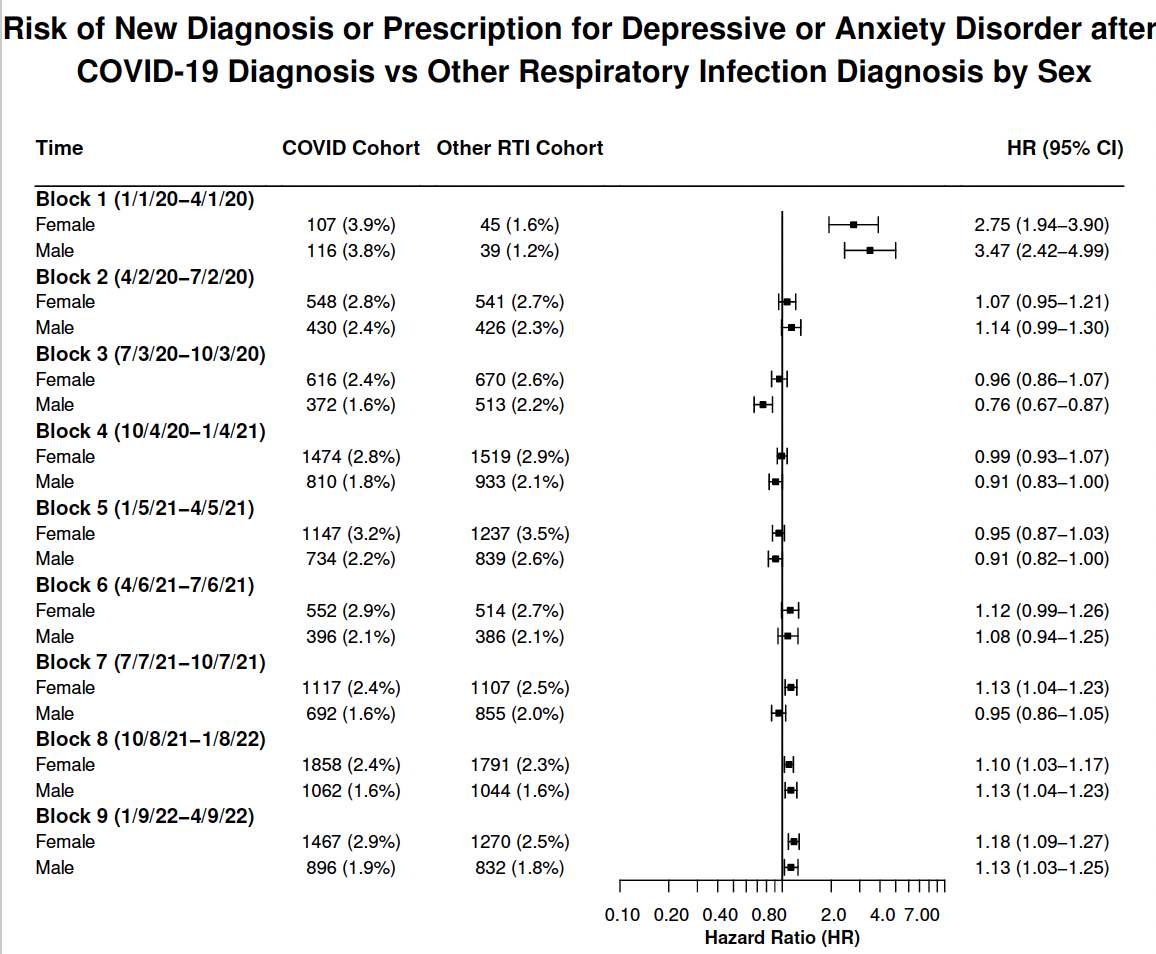


This plot shows HRs and 95% CIs stratified by sex when COVID-19 cohorts were compared to other RTI cohorts for the outcome of either a new depressive or anxiety disorder diagnosis or a new antidepressant or anxiolytic prescription.

**eFigure 7.**


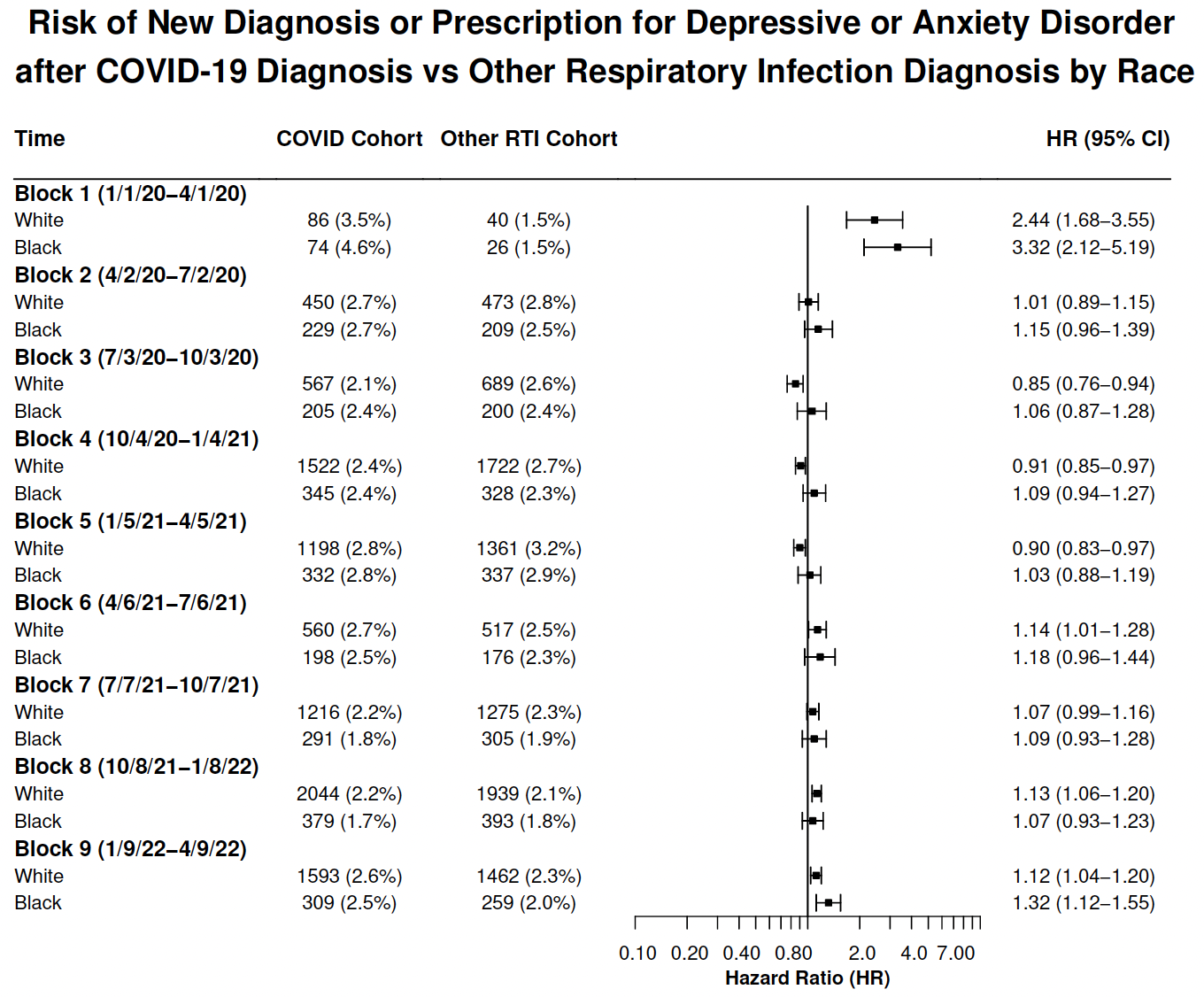


This plot shows HRs and 95% CIs stratified by sex when COVID-19 cohorts were compared to other RTI cohorts for the outcome of either a new depressive or anxiety disorder diagnosis or a new antidepressant or anxiolytic prescription.

**eFigure 8.**

**
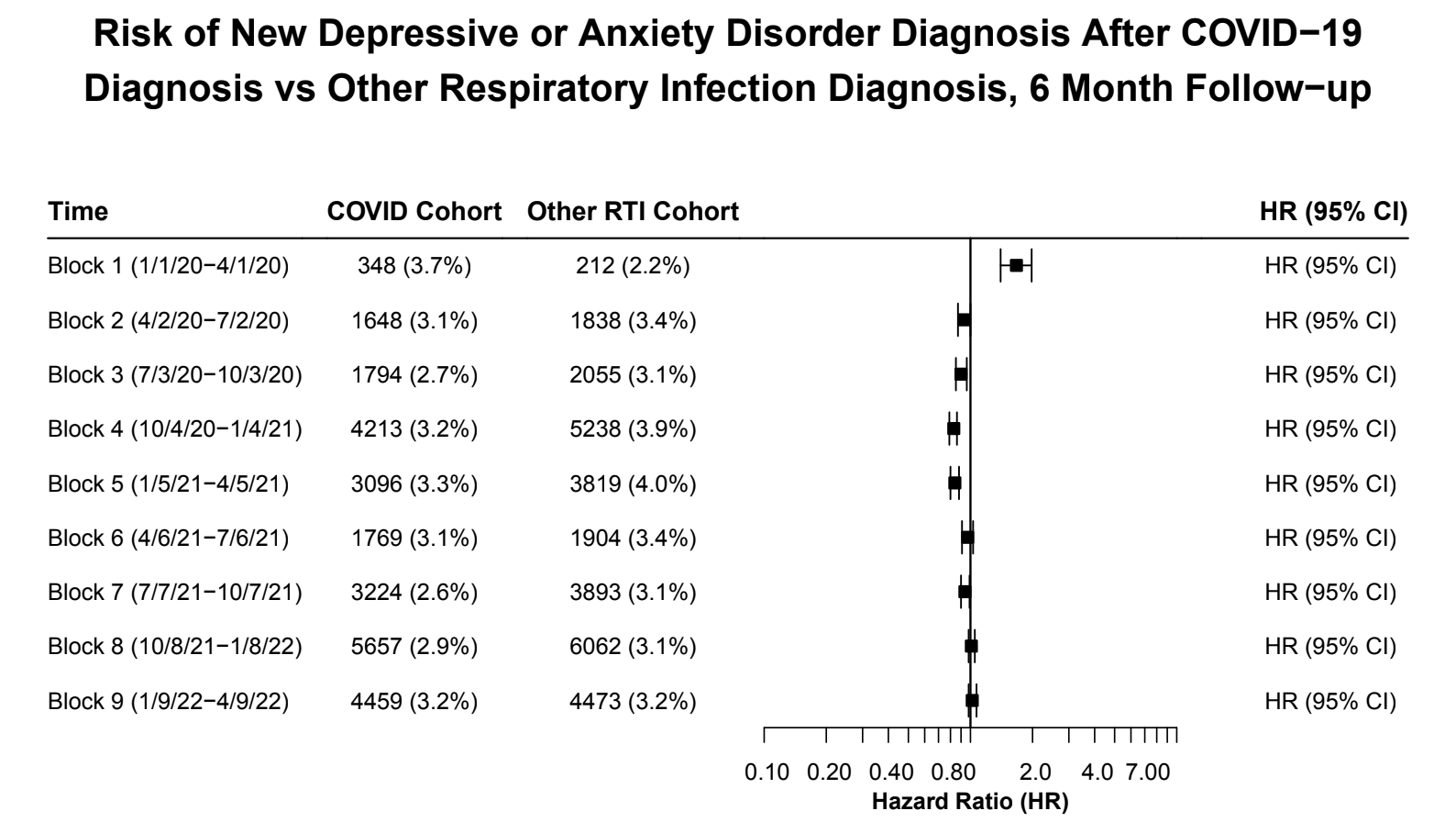
**

This plot shows the HRs and 95% CIs when COVID-19 cohorts were compared to other RTI cohorts for the outcome of a new depressive or anxiety disorder diagnosis 2 weeks to 6 months after the index event. **eFigure 9.**

**
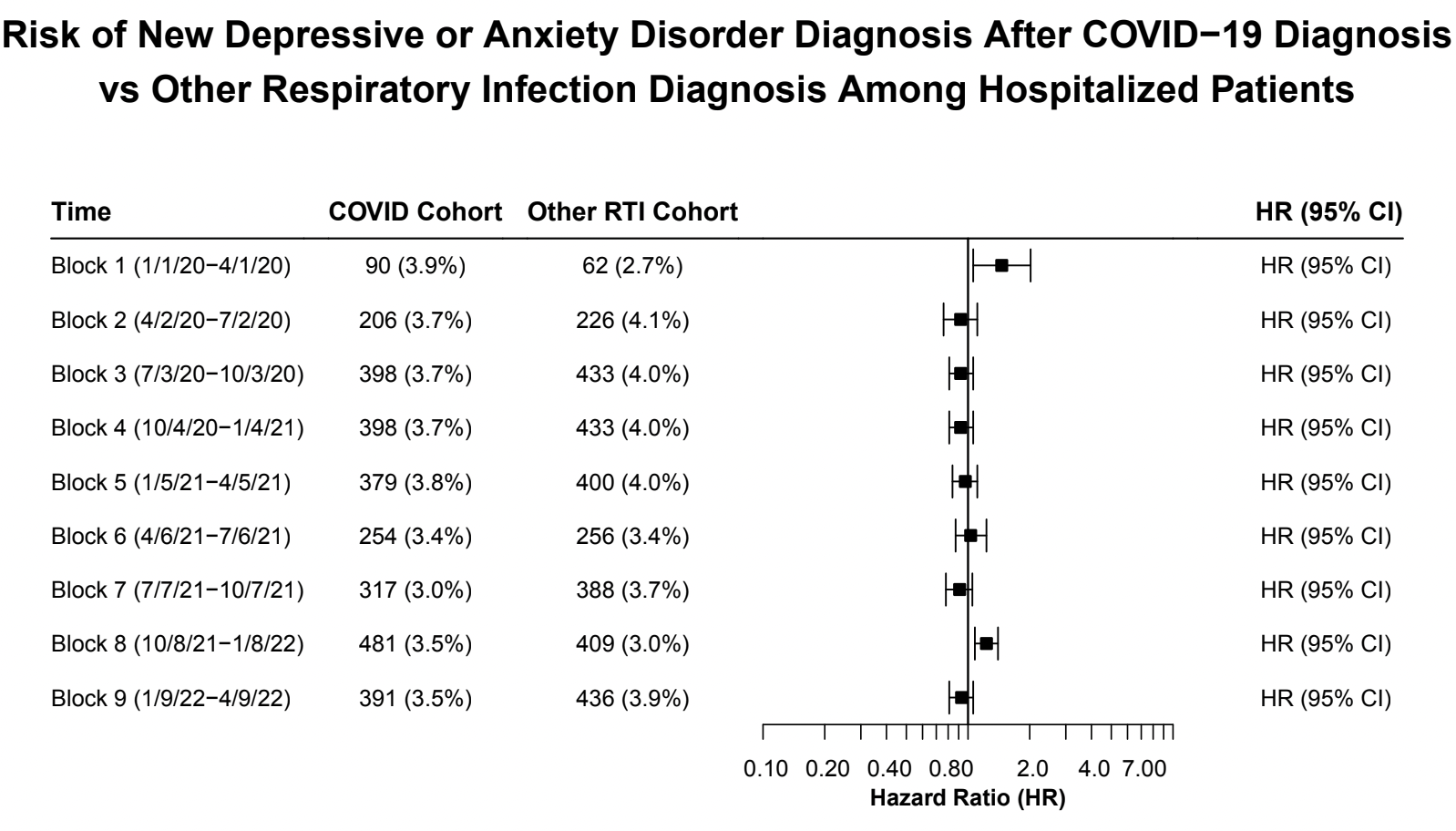
**

This plot shows the HRs and 95% CIs when COVID-19 cohorts were compared to other RTI cohorts among patients who had an inpatient encounter one month prior to one month after the index event for the outcome of a new depressive or anxiety disorder diagnosis.

**Table 1. Baseline Characteristics for Block 1 Before and After Matching**

|  | Before matching | | | After matching | | |
| --- | --- | --- | --- | --- | --- | --- |
|  | Cohort, No. (%) | | | Cohort, No. (%) | | |
| Characteristics | COVID-19 | Other RTI | SMD | COVID-19 | Other RTI | SMD |
| Total number | 7298 | 465735 |  | 7289 | 7289 |  |
| Age at index, mean (SD), y | 46.0 (21.5) | 28.8 (24.7) | 0.74 | 46.0 (21.5) | 47.4 (23.1) | 0.06 |
| Current age, mean (SD), y | 48.9 (21.4) | 31.7 (24.7) | 0.74 | 48.8 (21.4) | 50.3 (22.9) | 0.07 |
| Gender | | | | | | |
| Female | 3495 (47.9%) | 248485 (53.4%) | 0.11 | 3492 (47.9%) | 3533 (48.5%) | 0.01 |
| Male | 3800 (52.1%) | 217188 (46.6%) | 0.11 | 3794 (52.1%) | 3754 (51.5%) | 0.01 |
| Unknown | 10 (0.1%) | 62 (0.0%) | 0.05 | 10 (0.1%) | 10 (0.1%) | 0.00 |
| Race | | | | | | |
| White | 3225 (44.2%) | 299112 (64.2%) | 0.41 | 3223 (44.2%) | 3284 (45.1%) | 0.02 |
| Black or African American | 2275 (31.2%) | 81420 (17.5%) | 0.32 | 2274 (31.2%) | 2191 (30.1%) | 0.02 |
| Asian | 245 (3.4%) | 14137 (3.0%) | 0.02 | 245 (3.4%) | 251 (3.4%) | 0.00 |
| American Indian or Alaska  Native | 20 (0.3%) | 1424 (0.3%) | 0.01 | 20 (0.3%) | 24 (0.3%) | 0.01 |
| Native Hawaiian or Other  Pacific Islander | 14 (0.2%) | 816 (0.2%) | 0.00 | 14 (0.2%) | 10 (0.1%) | 0.01 |
| Unknown race | 1519 (20.8%) | 68826 (14.8%) | 0.16 | 1513 (20.8%) | 1532 (21.0%) | 0.01 |
| Ethnicity | | | | | | |
| Hispanic or Latino | 1155 (15.8%) | 61498 (13.2%) | 0.07 | 1150 (15.8%) | 1132 (15.5%) | 0.01 |
| Not Hispanic or Latino | 4677 (64.1%) | 315721 (67.8%) | 0.08 | 4673 (64.1%) | 4681 (64.2%) | 0.00 |
| Unknown ethnicity | 1466 (20.1%) | 88516 (19.0%) | 0.03 | 1466 (20.1%) | 1476 (20.3%) | 0.00 |
| Overweight and obesity | 1010 (13.8%) | 43335 (9.3%) | 0.14 | 1005 (13.8%) | 917 (12.6%) | 0.04 |
| Hypertensive diseases | 2050 (28.1%) | 71135 (15.3%) | 0.31 | 2043 (28.0%) | 1894 (26.0%) | 0.05 |
| Type 1 diabetes mellitus | 130 (1.8%) | 3561 (0.8%) | 0.09 | 130 (1.8%) | 107 (1.5%) | 0.02 |
| Type 2 diabetes mellitus | 1024 (14.0%) | 29396 (6.3%) | 0.26 | 1020 (14.0%) | 901 (12.4%) | 0.05 |
| Bronchitis, not specified as acute or chronic | 242 (3.3%) | 20753 (4.5%) | 0.06 | 242 (3.3%) | 227 (3.1%) | 0.01 |
| Simple and mucopurulent chronic bronchitis | 15 (0.2%) | 1340 (0.3%) | 0.02 | 15 (0.2%) | 12 (0.2%) | 0.01 |
| Unspecified chronic bronchitis | 29 (0.4%) | 1334 (0.3%) | 0.02 | 29 (0.4%) | 22 (0.3%) | 0.02 |
| Emphysema | 136 (1.9%) | 4525 (1.0%) | 0.08 | 136 (1.9%) | 121 (1.7%) | 0.02 |
| Other chronic obstructive pulmonary disease | 298 (4.1%) | 11444 (2.5%) | 0.09 | 297 (4.1%) | 279 (3.8%) | 0.01 |
| Asthma | 661 (9.1%) | 53772 (11.5%) | 0.08 | 661 (9.1%) | 645 (8.8%) | 0.01 |
| Bronchiectasis | 40 (0.5%) | 2007 (0.4%) | 0.02 | 40 (0.5%) | 37 (0.5%) | 0.01 |
| Other forms of heart disease | 1244 (17.0%) | 39676 (8.5%) | 0.26 | 1238 (17.0%) | 1062 (14.6%) | 0.07 |
| Hypertensive chronic kidney disease | 268 (3.7%) | 5076 (1.1%) | 0.17 | 267 (3.7%) | 220 (3.0%) | 0.04 |
| Hepatic failure, not elsewhere classified | 25 (0.3%) | 685 (0.1%) | 0.04 | 25 (0.3%) | 18 (0.2%) | 0.02 |
| Chronic hepatitis, not elsewhere classified | 10 (0.1%) | 256 (0.1%) | 0.03 | 10 (0.1%) | 11 (0.2%) | 0.00 |
| Fibrosis and cirrhosis of liver | 55 (0.8%) | 1848 (0.4%) | 0.05 | 55 (0.8%) | 55 (0.8%) | 0.00 |
| Fatty (change of) liver, not elsewhere classified | 178 (2.4%) | 6145 (1.3%) | 0.08 | 177 (2.4%) | 164 (2.3%) | 0.01 |
| Chronic passive congestion of liver | 34 (0.5%) | 1031 (0.2%) | 0.04 | 34 (0.5%) | 36 (0.5%) | 0.00 |
| Portal hypertension | 19 (0.3%) | 650 (0.1%) | 0.03 | 19 (0.3%) | 18 (0.2%) | 0.00 |
| Other specified diseases of liver | 135 (1.9%) | 4386 (0.9%) | 0.08 | 135 (1.9%) | 128 (1.8%) | 0.01 |
| Cerebral infarction | 249 (3.4%) | 5833 (1.3%) | 0.14 | 247 (3.4%) | 196 (2.7%) | 0.04 |
| Vascular dementia | 24 (0.3%) | 275 (0.1%) | 0.06 | 22 (0.3%) | 21 (0.3%) | 0.00 |
| Dementia in other diseases classified elsewhere | 23 (0.3%) | 533 (0.1%) | 0.04 | 23 (0.3%) | 17 (0.2%) | 0.02 |
| Unspecified dementia | 64 (0.9%) | 1211 (0.3%) | 0.08 | 62 (0.9%) | 52 (0.7%) | 0.02 |
| Alzheimer's disease | 21 (0.3%) | 545 (0.1%) | 0.04 | 21 (0.3%) | 18 (0.2%) | 0.01 |
| Frontotemporal dementia | 10 (0.1%) | 34 (0.0%) | 0.05 | 10 (0.1%) | 10 (0.1%) | 0.00 |
| Neurocognitive disorder with Lewy bodies | 10 (0.1%) | 40 (0.0%) | 0.05 | 10 (0.1%) | 0 (0.0%) | 0.05 |
| Neoplasms | 1365 (18.7%) | 58395 (12.5%) | 0.17 | 1359 (18.6%) | 1294 (17.8%) | 0.02 |
| Malignant neoplasms of lymphoid, hematopoietic and related tissue | 128 (1.8%) | 3557 (0.8%) | 0.09 | 128 (1.8%) | 123 (1.7%) | 0.01 |
| Rheumatoid arthritis with rheumatoid factor | 26 (0.4%) | 1189 (0.3%) | 0.02 | 26 (0.4%) | 31 (0.4%) | 0.01 |
| Other rheumatoid arthritis | 87 (1.2%) | 3741 (0.8%) | 0.04 | 87 (1.2%) | 81 (1.1%) | 0.01 |
| Systemic lupus erythematosus (SLE) | 40 (0.5%) | 1317 (0.3%) | 0.04 | 40 (0.5%) | 30 (0.4%) | 0.02 |
| Psoriasis | 53 (0.7%) | 3412 (0.7%) | 0.00 | 53 (0.7%) | 51 (0.7%) | 0.00 |
| Certain disorders involving the immune mechanism | 222 (3.0%) | 5696 (1.2%) | 0.13 | 222 (3.0%) | 189 (2.6%) | 0.03 |
| Family history of mental and behavioral disorders | 75 (1.0%) | 3629 (0.8%) | 0.03 | 73 (1.0%) | 63 (0.9%) | 0.01 |
| Persons with potential health hazards related to socioeconomic and psychosocial circumstances | 211 (2.9%) | 10778 (2.3%) | 0.04 | 209 (2.9%) | 143 (2.0%) | 0.06 |
| Personal history of other mental and behavioral disorders | 22 (0.3%) | 908 (0.2%) | 0.02 | 22 (0.3%) | 21 (0.3%) | 0.00 |
| Nicotine dependence | 451 (6.2%) | 25469 (5.5%) | 0.03 | 451 (6.2%) | 460 (6.3%) | 0.01 |
| Alcohol related disorders | 252 (3.5%) | 4720 (1.0%) | 0.17 | 247 (3.4%) | 205 (2.8%) | 0.03 |
| Other psychoactive substance related disorders | 209 (2.9%) | 3111 (0.7%) | 0.17 | 201 (2.8%) | 133 (1.8%) | 0.06 |
| Cannabis related disorders | 55 (0.8%) | 2180 (0.5%) | 0.04 | 55 (0.8%) | 45 (0.6%) | 0.02 |
| Other stimulant related disorders | 26 (0.4%) | 1263 (0.3%) | 0.02 | 26 (0.4%) | 22 (0.3%) | 0.01 |
| Opioid related disorders | 38 (0.5%) | 1508 (0.3%) | 0.03 | 38 (0.5%) | 30 (0.4%) | 0.02 |
| Cocaine related disorders | 34 (0.5%) | 823 (0.2%) | 0.05 | 34 (0.5%) | 22 (0.3%) | 0.03 |
| Hallucinogen related disorders | 12 (0.2%) | 189 (0.0%) | 0.04 | 11 (0.2%) | 10 (0.1%) | 0.00 |
| Inhalant related disorders | 11 (0.2%) | 458 (0.1%) | 0.01 | 11 (0.2%) | 11 (0.2%) | 0.00 |
| Sedative, hypnotic, or anxiolytic related disorders | 10 (0.1%) | 242 (0.1%) | 0.03 | 10 (0.1%) | 10 (0.1%) | 0.00 |
| Unspecified psychosis not due to a substance or known physiological condition | 19 (0.3%) | 426 (0.1%) | 0.04 | 19 (0.3%) | 14 (0.2%) | 0.01 |
| Schizophrenia | 16 (0.2%) | 430 (0.1%) | 0.03 | 16 (0.2%) | 10 (0.1%) | 0.02 |
| Schizoaffective disorders | 10 (0.1%) | 195 (0.0%) | 0.03 | 10 (0.1%) | 10 (0.1%) | 0.00 |
| Delusional disorders | 10 (0.1%) | 189 (0.0%) | 0.03 | 10 (0.1%) | 10 (0.1%) | 0.00 |
| Brief psychotic disorder | 10 (0.1%) | 45 (0.0%) | 0.05 | 10 (0.1%) | 0 (0.0%) | 0.05 |
| Other psychotic disorder not due to a substance or known physiological condition | 10 (0.1%) | 11 (0.0%) | 0.05 | 10 (0.1%) | 10 (0.1%) | 0.00 |
| Schizotypal disorder | 0 (0.0%) | 10 (0.0%) | 0.01 | 0 (0.0%) | 0 (0.0%) |  |
| Shared psychotic disorder | 0 (0.0%) | 10 (0.0%) | 0.01 | 0 (0.0%) | 0 (0.0%) |  |
| Renal Transplantation Procedures | 20 (0.3%) | 159 (0.0%) | 0.06 | 20 (0.3%) | 10 (0.1%) | 0.03 |
| Liver Transplantation Procedures | 10 (0.1%) | 54 (0.0%) | 0.05 | 10 (0.1%) | 0 (0.0%) | 0.05 |
| Blood Pressure, Systolic | | | | | | |
| <140 mm[Hg] | 3151 (43.2%) | 193455 (41.5%) | 0.03 | 3143 (43.1%) | 2904 (39.8%) | 0.07 |
| 140 - 160 mm[Hg] | 1774 (24.3%) | 62206 (13.4%) | 0.28 | 1767 (24.2%) | 1614 (22.1%) | 0.05 |
| >160[Hg] | 893 (12.2%) | 28100 (6.0%) | 0.22 | 888 (12.2%) | 773 (10.6%) | 0.05 |
| Blood Pressure, Diastolic | | | | | | |
| 0 - 90 mm[Hg] | 3234 (44.3%) | 195938 (42.1%) | 0.05 | 3226 (44.3%) | 2990 (41.0%) | 0.07 |
| 90 - 100 mm[Hg] | 1293 (17.7%) | 46399 (10.0%) | 0.23 | 1286 (17.6%) | 1161 (15.9%) | 0.05 |
| 100 - 0 mm[Hg] | 597 (8.2%) | 18641 (4.0%) | 0.18 | 594 (8.1%) | 534 (7.3%) | 0.03 |
| BMI | | | | | | |
| 0 - 25 kg/m2 | 1166 (16.0%) | 103966 (22.3%) | 0.16 | 1162 (15.9%) | 1001 (13.7%) | 0.06 |
| 25 - 30 kg/m2 | 1285 (17.6%) | 45888 (9.9%) | 0.23 | 1278 (17.5%) | 1183 (16.2%) | 0.03 |
| 30 - 0 kg/m2 | 1361 (18.6%) | 42630 (9.2%) | 0.28 | 1355 (18.6%) | 1265 (17.4%) | 0.03 |
| New or Established Patient | 679 (9.3%) | 19492 (4.2%) | 0.21 | 671 (9.2%) | 571 (7.8%) | 0.05 |
| Hospital Inpatient Services | 862 (11.8%) | 27052 (5.8%) | 0.21 | 854 (11.7%) | 729 (10.0%) | 0.06 |
| Critical Care Services | 323 (4.4%) | 6708 (1.4%) | 0.18 | 320 (4.4%) | 250 (3.4%) | 0.05 |
| Visit: Inpatient Encounter | 1612 (22.1%) | 76068 (16.3%) | 0.15 | 1604 (22.0%) | 1396 (19.2%) | 0.07 |
| Visit: Short Stay | 416 (5.7%) | 5036 (1.1%) | 0.26 | 409 (5.6%) | 319 (4.4%) | 0.06 |
| Visit: Inpatient Non-acute | 10 (0.1%) | 589 (0.1%) | 0.00 | 10 (0.1%) | 10 (0.1%) | 0.00 |
| Severe acute respiratory syndrome coronavirus 2 (SARS-CoV-2) (coronavirus disease [COVID-19]) vaccine, mRNA-LNP, spike protein, preservative free, 30 mcg/0.3 mL dosage, diluent reconstituted, for intramuscular use | 0 (0.0%) | 0 (0.0%) |  | 0 (0.0%) | 0 (0.0%) |  |
| Immunization administration by intramuscular injection of severe acute respiratory syndrome coronavirus 2 (SARS-CoV-2) (coronavirus disease [COVID-19]) vaccine, mRNA-LNP, spike protein, preservative free, 30 mcg/0.3 mL dosage, diluent reconstituted; first dose | 0 (0.0%) | 0 (0.0%) |  | 0 (0.0%) | 0 (0.0%) |  |
| Immunization administration by intramuscular injection of severe acute respiratory syndrome coronavirus 2 (SARS-CoV-2) (coronavirus disease [COVID-19]) vaccine, mRNA-LNP, spike protein, preservative free, 30 mcg/0.3 mL dosage, diluent reconstituted; second dose | 0 (0.0%) | 0 (0.0%) |  | 0 (0.0%) | 0 (0.0%) |  |
| Severe acute respiratory syndrome coronavirus 2 (SARS-CoV-2) (coronavirus disease [COVID-19]) vaccine, mRNA-LNP, spike protein, preservative free, 100 mcg/0.5 mL dosage, for intramuscular use | 0 (0.0%) | 0 (0.0%) |  | 0 (0.0%) | 0 (0.0%) |  |
| Immunization administration by intramuscular injection of severe acute respiratory syndrome coronavirus 2 (SARS-CoV-2) (coronavirus disease [COVID-19]) vaccine, mRNA-LNP, spike protein, preservative free, 100 mcg/0.5 mL dosage; first dose | 0 (0.0%) | 0 (0.0%) |  | 0 (0.0%) | 0 (0.0%) |  |
| Immunization administration by intramuscular injection of severe acute respiratory syndrome coronavirus 2 (SARS-CoV-2) (coronavirus disease [COVID-19]) vaccine, mRNA-LNP, spike protein, preservative free, 100 mcg/0.5 mL dosage; second dose | 0 (0.0%) | 0 (0.0%) |  | 0 (0.0%) | 0 (0.0%) |  |
| Severe acute respiratory syndrome coronavirus 2 (SARS-CoV-2) (coronavirus disease [COVID-19]) vaccine, DNA, spike protein, chimpanzee adenovirus Oxford 1 (ChAdOx1) vector, preservative free, 5x1010 viral particles/0.5 mL dosage, for intramuscular use | 0 (0.0%) | 0 (0.0%) |  | 0 (0.0%) | 0 (0.0%) |  |
| Immunization administration by intramuscular injection of severe acute respiratory syndrome coronavirus 2 (SARS-CoV-2) (coronavirus disease [COVID-19]) vaccine, DNA, spike protein, chimpanzee adenovirus Oxford 1 (ChAdOx1) vector, preservative free, 5x1010 viral particles/0.5 mL dosage; first dose | 0 (0.0%) | 0 (0.0%) |  | 0 (0.0%) | 0 (0.0%) |  |
| Immunization administration by intramuscular injection of severe acute respiratory syndrome coronavirus 2 (SARS-CoV-2) (coronavirus disease [COVID-19]) vaccine, DNA, spike protein, chimpanzee adenovirus Oxford 1 (ChAdOx1) vector, preservative free, 5x1010 viral particles/0.5 mL dosage; second dose | 0 (0.0%) | 0 (0.0%) |  | 0 (0.0%) | 0 (0.0%) |  |
| Severe acute respiratory syndrome coronavirus 2 (SARS-CoV-2) (coronavirus disease [COVID-19]) vaccine, DNA, spike protein, adenovirus type 26 (Ad26) vector, preservative free, 5x1010 viral particles/0.5 mL dosage, for intramuscular use | 0 (0.0%) | 0 (0.0%) |  | 0 (0.0%) | 0 (0.0%) |  |
| Immunization administration by intramuscular injection of severe acute respiratory syndrome coronavirus 2 (SARS-CoV-2) (coronavirus disease [COVID-19]) vaccine, DNA, spike protein, adenovirus type 26 (Ad26) vector, preservative free, 5x1010 viral particles/0.5 mL dosage, single dose | 0 (0.0%) | 0 (0.0%) |  | 0 (0.0%) | 0 (0.0%) |  |
| Introduction of COVID-19 Vaccine into Subcutaneous Tissue, Percutaneous Approach, New Technology Group 6 | 0 (0.0%) | 0 (0.0%) |  | 0 (0.0%) | 0 (0.0%) |  |
| Introduction of COVID-19 Vaccine Dose 1 into Subcutaneous Tissue, Percutaneous Approach, New Technology Group 6 | 0 (0.0%) | 0 (0.0%) |  | 0 (0.0%) | 0 (0.0%) |  |
| Introduction of COVID-19 Vaccine Dose 2 into Subcutaneous Tissue, Percutaneous Approach, New Technology Group 6 | 0 (0.0%) | 0 (0.0%) |  | 0 (0.0%) | 0 (0.0%) |  |
| Introduction of COVID-19 Vaccine into Muscle, Percutaneous Approach, New Technology Group 6 | 0 (0.0%) | 0 (0.0%) |  | 0 (0.0%) | 0 (0.0%) |  |
| Introduction of COVID-19 Vaccine Dose 1 into Muscle, Percutaneous Approach, New Technology Group 6 | 0 (0.0%) | 0 (0.0%) |  | 0 (0.0%) | 0 (0.0%) |  |
| Introduction of COVID-19 Vaccine Dose 2 into Muscle, Percutaneous Approach, New Technology Group 6 | 0 (0.0%) | 0 (0.0%) |  | 0 (0.0%) | 0 (0.0%) |  |
| SARS-CoV-2 (COVID-19) Vaccine | 10 (0.1%) | 10 (0.0%) | 0.05 | 10 (0.1%) | 0 (0.0%) | 0.05 |

**Table 2. Baseline Characteristics for Block 2 Before and After Matching**

|  | Before matching | | | After matching | | |
| --- | --- | --- | --- | --- | --- | --- |
|  | Cohort, No. (%) | | | Cohort, No. (%) | | |
| Characteristics | COVID-19 | Other RTI | SMD | COVID-19 | Other RTI | SMD |
| Total number | 50687 | 96233 |  | 45407 | 45407 |  |
| Age at index, mean (SD), y | 46.1 (19.7) | 39.9 (24.0) | 0.28 | 44.8 (19.5) | 45.8 (21.6) | 0.05 |
| Current age, mean (SD), y | 48.8 (19.6) | 42.6 (23.9) | 0.28 | 47.5 (19.4) | 48.5 (21.5) | 0.05 |
| Gender | | | | | | |
| Female | 26444 (52.2%) | 52163 (54.2%) | 0.04 | 23992 (52.8%) | 24297 (53.5%) | 0.01 |
| Male | 24176 (47.7%) | 44001 (45.7%) | 0.04 | 21357 (47.0%) | 21052 (46.4%) | 0.01 |
| Unknown | 67 (0.1%) | 69 (0.1%) | 0.02 | 58 (0.1%) | 58 (0.1%) | 0.00 |
| Race | | | | | | |
| White | 22375 (44.1%) | 58778 (61.1%) | 0.34 | 21500 (47.4%) | 21740 (47.9%) | 0.01 |
| Black or African American | 13753 (27.1%) | 19256 (20.0%) | 0.17 | 12075 (26.6%) | 11791 (26.0%) | 0.01 |
| Asian | 1548 (3.1%) | 2734 (2.8%) | 0.01 | 1451 (3.2%) | 1449 (3.2%) | 0.00 |
| American Indian or Alaska  Native | 156 (0.3%) | 301 (0.3%) | 0.00 | 139 (0.3%) | 141 (0.3%) | 0.00 |
| Native Hawaiian or Other  Pacific Islander | 123 (0.2%) | 182 (0.2%) | 0.01 | 104 (0.2%) | 109 (0.2%) | 0.00 |
| Unknown race | 12732 (25.1%) | 14982 (15.6%) | 0.24 | 10138 (22.3%) | 10177 (22.4%) | 0.00 |
| Ethnicity | | | | | | |
| Hispanic or Latino | 11640 (23.0%) | 11385 (11.8%) | 0.30 | 8852 (19.5%) | 8202 (18.1%) | 0.04 |
| Not Hispanic or Latino | 25702 (50.7%) | 66430 (69.0%) | 0.38 | 24928 (54.9%) | 25317 (55.8%) | 0.02 |
| Unknown ethnicity | 13345 (26.3%) | 18418 (19.1%) | 0.17 | 11627 (25.6%) | 11888 (26.2%) | 0.01 |
| Overweight and obesity | 6769 (13.4%) | 11166 (11.6%) | 0.05 | 5947 (13.1%) | 5833 (12.8%) | 0.01 |
| Hypertensive diseases | 11647 (23.0%) | 22341 (23.2%) | 0.01 | 10466 (23.0%) | 10296 (22.7%) | 0.01 |
| Type 1 diabetes mellitus | 742 (1.5%) | 1126 (1.2%) | 0.03 | 607 (1.3%) | 625 (1.4%) | 0.00 |
| Type 2 diabetes mellitus | 6159 (12.2%) | 9244 (9.6%) | 0.08 | 5175 (11.4%) | 5073 (11.2%) | 0.01 |
| Bronchitis, not specified as acute or chronic | 1339 (2.6%) | 4844 (5.0%) | 0.12 | 1309 (2.9%) | 1361 (3.0%) | 0.01 |
| Simple and mucopurulent chronic bronchitis | 94 (0.2%) | 524 (0.5%) | 0.06 | 92 (0.2%) | 110 (0.2%) | 0.01 |
| Unspecified chronic bronchitis | 114 (0.2%) | 565 (0.6%) | 0.06 | 112 (0.2%) | 133 (0.3%) | 0.01 |
| Emphysema | 472 (0.9%) | 1975 (2.1%) | 0.09 | 455 (1.0%) | 497 (1.1%) | 0.01 |
| Other chronic obstructive pulmonary disease | 1202 (2.4%) | 4603 (4.8%) | 0.13 | 1168 (2.6%) | 1203 (2.6%) | 0.00 |
| Asthma | 3426 (6.8%) | 11144 (11.6%) | 0.17 | 3322 (7.3%) | 3312 (7.3%) | 0.00 |
| Bronchiectasis | 179 (0.4%) | 950 (1.0%) | 0.08 | 179 (0.4%) | 213 (0.5%) | 0.01 |
| Other forms of heart disease | 6196 (12.2%) | 13279 (13.8%) | 0.05 | 5627 (12.4%) | 5642 (12.4%) | 0.00 |
| Hypertensive chronic kidney disease | 1184 (2.3%) | 2138 (2.2%) | 0.01 | 1017 (2.2%) | 1007 (2.2%) | 0.00 |
| Hepatic failure, not elsewhere classified | 149 (0.3%) | 337 (0.4%) | 0.01 | 134 (0.3%) | 149 (0.3%) | 0.01 |
| Chronic hepatitis, not elsewhere classified | 33 (0.1%) | 82 (0.1%) | 0.01 | 31 (0.1%) | 36 (0.1%) | 0.00 |
| Fibrosis and cirrhosis of liver | 367 (0.7%) | 720 (0.7%) | 0.00 | 325 (0.7%) | 349 (0.8%) | 0.01 |
| Fatty (change of) liver, not elsewhere classified | 1122 (2.2%) | 2039 (2.1%) | 0.01 | 982 (2.2%) | 953 (2.1%) | 0.00 |
| Chronic passive congestion of liver | 169 (0.3%) | 387 (0.4%) | 0.01 | 158 (0.3%) | 172 (0.4%) | 0.01 |
| Portal hypertension | 131 (0.3%) | 317 (0.3%) | 0.01 | 120 (0.3%) | 140 (0.3%) | 0.01 |
| Other specified diseases of liver | 722 (1.4%) | 1525 (1.6%) | 0.01 | 639 (1.4%) | 664 (1.5%) | 0.00 |
| Cerebral infarction | 1139 (2.2%) | 2047 (2.1%) | 0.01 | 973 (2.1%) | 1032 (2.3%) | 0.01 |
| Vascular dementia | 140 (0.3%) | 107 (0.1%) | 0.04 | 84 (0.2%) | 86 (0.2%) | 0.00 |
| Dementia in other diseases classified elsewhere | 241 (0.5%) | 239 (0.2%) | 0.04 | 176 (0.4%) | 170 (0.4%) | 0.00 |
| Unspecified dementia | 521 (1.0%) | 496 (0.5%) | 0.06 | 355 (0.8%) | 359 (0.8%) | 0.00 |
| Alzheimer's disease | 220 (0.4%) | 221 (0.2%) | 0.04 | 164 (0.4%) | 160 (0.4%) | 0.00 |
| Frontotemporal dementia | 10 (0.0%) | 10 (0.0%) | 0.01 | 10 (0.0%) | 10 (0.0%) | 0.00 |
| Neurocognitive disorder with Lewy bodies | 20 (0.0%) | 15 (0.0%) | 0.01 | 15 (0.0%) | 12 (0.0%) | 0.00 |
| Neoplasms | 7550 (14.9%) | 17199 (17.9%) | 0.08 | 7002 (15.4%) | 7064 (15.6%) | 0.00 |
| Malignant neoplasms of lymphoid, hematopoietic and related tissue | 433 (0.9%) | 1247 (1.3%) | 0.04 | 413 (0.9%) | 402 (0.9%) | 0.00 |
| Rheumatoid arthritis with rheumatoid factor | 136 (0.3%) | 404 (0.4%) | 0.03 | 129 (0.3%) | 128 (0.3%) | 0.00 |
| Other rheumatoid arthritis | 411 (0.8%) | 1318 (1.4%) | 0.05 | 397 (0.9%) | 388 (0.9%) | 0.00 |
| Systemic lupus erythematosus (SLE) | 174 (0.3%) | 488 (0.5%) | 0.03 | 167 (0.4%) | 165 (0.4%) | 0.00 |
| Psoriasis | 370 (0.7%) | 949 (1.0%) | 0.03 | 349 (0.8%) | 345 (0.8%) | 0.00 |
| Certain disorders involving the immune mechanism | 723 (1.4%) | 2022 (2.1%) | 0.05 | 673 (1.5%) | 688 (1.5%) | 0.00 |
| Family history of mental and behavioral disorders | 128 (0.3%) | 649 (0.7%) | 0.06 | 123 (0.3%) | 154 (0.3%) | 0.01 |
| Persons with potential health hazards related to socioeconomic and psychosocial circumstances | 1006 (2.0%) | 2162 (2.2%) | 0.02 | 895 (2.0%) | 898 (2.0%) | 0.00 |
| Personal history of other mental and behavioral disorders | 87 (0.2%) | 221 (0.2%) | 0.01 | 81 (0.2%) | 78 (0.2%) | 0.00 |
| Nicotine dependence | 2486 (4.9%) | 7712 (8.0%) | 0.13 | 2403 (5.3%) | 2470 (5.4%) | 0.01 |
| Alcohol related disorders | 899 (1.8%) | 1722 (1.8%) | 0.00 | 772 (1.7%) | 766 (1.7%) | 0.00 |
| Other psychoactive substance related disorders | 494 (1.0%) | 1121 (1.2%) | 0.02 | 437 (1.0%) | 455 (1.0%) | 0.00 |
| Cannabis related disorders | 286 (0.6%) | 785 (0.8%) | 0.03 | 275 (0.6%) | 288 (0.6%) | 0.00 |
| Other stimulant related disorders | 97 (0.2%) | 368 (0.4%) | 0.04 | 95 (0.2%) | 104 (0.2%) | 0.00 |
| Opioid related disorders | 230 (0.5%) | 680 (0.7%) | 0.03 | 216 (0.5%) | 229 (0.5%) | 0.00 |
| Cocaine related disorders | 191 (0.4%) | 355 (0.4%) | 0.00 | 163 (0.4%) | 178 (0.4%) | 0.01 |
| Hallucinogen related disorders | 24 (0.0%) | 112 (0.1%) | 0.02 | 24 (0.1%) | 30 (0.1%) | 0.01 |
| Inhalant related disorders | 74 (0.1%) | 179 (0.2%) | 0.01 | 68 (0.2%) | 78 (0.2%) | 0.01 |
| Sedative, hypnotic, or anxiolytic related disorders | 16 (0.0%) | 85 (0.1%) | 0.02 | 16 (0.0%) | 13 (0.0%) | 0.00 |
| Unspecified psychosis not due to a substance or known physiological condition | 124 (0.2%) | 184 (0.2%) | 0.01 | 108 (0.2%) | 96 (0.2%) | 0.01 |
| Schizophrenia | 164 (0.3%) | 189 (0.2%) | 0.02 | 126 (0.3%) | 126 (0.3%) | 0.00 |
| Schizoaffective disorders | 81 (0.2%) | 73 (0.1%) | 0.02 | 57 (0.1%) | 52 (0.1%) | 0.00 |
| Delusional disorders | 40 (0.1%) | 57 (0.1%) | 0.01 | 34 (0.1%) | 27 (0.1%) | 0.01 |
| Brief psychotic disorder | 15 (0.0%) | 16 (0.0%) | 0.01 | 12 (0.0%) | 10 (0.0%) | 0.00 |
| Other psychotic disorder not due to a substance or known physiological condition | 10 (0.0%) | 10 (0.0%) | 0.01 | 10 (0.0%) | 10 (0.0%) | 0.00 |
| Schizotypal disorder | 10 (0.0%) | 10 (0.0%) | 0.01 | 10 (0.0%) | 10 (0.0%) | 0.00 |
| Shared psychotic disorder | 10 (0.0%) | 10 (0.0%) | 0.01 | 10 (0.0%) | 10 (0.0%) | 0.00 |
| Renal Transplantation Procedures | 77 (0.2%) | 76 (0.1%) | 0.02 | 56 (0.1%) | 55 (0.1%) | 0.00 |
| Liver Transplantation Procedures | 12 (0.0%) | 23 (0.0%) | 0.00 | 11 (0.0%) | 10 (0.0%) | 0.00 |
| Blood Pressure, Systolic | | | | | | |
| <140 mm[Hg] | 19195 (37.9%) | 40500 (42.1%) | 0.09 | 17351 (38.2%) | 16881 (37.2%) | 0.02 |
| 140 - 160 mm[Hg] | 9841 (19.4%) | 18619 (19.3%) | 0.00 | 8639 (19.0%) | 8334 (18.4%) | 0.02 |
| >160[Hg] | 4948 (9.8%) | 9119 (9.5%) | 0.01 | 4261 (9.4%) | 4065 (9.0%) | 0.01 |
| Blood Pressure, Diastolic | | | | | | |
| 0 - 90 mm[Hg] | 19936 (39.3%) | 40735 (42.3%) | 0.06 | 17871 (39.4%) | 17398 (38.3%) | 0.02 |
| 90 - 100 mm[Hg] | 7056 (13.9%) | 13483 (14.0%) | 0.00 | 6193 (13.6%) | 5993 (13.2%) | 0.01 |
| 100 - 0 mm[Hg] | 3166 (6.2%) | 5838 (6.1%) | 0.01 | 2743 (6.0%) | 2639 (5.8%) | 0.01 |
| BMI | | | | | | |
| 0 - 25 kg/m2 | 5784 (11.4%) | 17294 (18.0%) | 0.19 | 5450 (12.0%) | 5425 (11.9%) | 0.00 |
| 25 - 30 kg/m2 | 6850 (13.5%) | 13005 (13.5%) | 0.00 | 6017 (13.3%) | 6045 (13.3%) | 0.00 |
| 30 - 0 kg/m2 | 7456 (14.7%) | 12160 (12.6%) | 0.06 | 6361 (14.0%) | 6201 (13.7%) | 0.01 |
| New or Established Patient | 3112 (6.1%) | 6390 (6.6%) | 0.02 | 2763 (6.1%) | 2676 (5.9%) | 0.01 |
| Hospital Inpatient Services | 3864 (7.6%) | 8165 (8.5%) | 0.03 | 3457 (7.6%) | 3338 (7.4%) | 0.01 |
| Critical Care Services | 1149 (2.3%) | 2484 (2.6%) | 0.02 | 1020 (2.2%) | 991 (2.2%) | 0.00 |
| Visit: Inpatient Encounter | 11125 (21.9%) | 20799 (21.6%) | 0.01 | 9793 (21.6%) | 9421 (20.7%) | 0.02 |
| Visit: Short Stay | 1732 (3.4%) | 1383 (1.4%) | 0.13 | 1109 (2.4%) | 1074 (2.4%) | 0.01 |
| Visit: Inpatient Non-acute | 118 (0.2%) | 118 (0.1%) | 0.03 | 77 (0.2%) | 65 (0.1%) | 0.01 |
| Severe acute respiratory syndrome coronavirus 2 (SARS-CoV-2) (coronavirus disease [COVID-19]) vaccine, mRNA-LNP, spike protein, preservative free, 30 mcg/0.3 mL dosage, diluent reconstituted, for intramuscular use | 0 (0.0%) | 0 (0.0%) |  | 0 (0.0%) | 0 (0.0%) |  |
| Immunization administration by intramuscular injection of severe acute respiratory syndrome coronavirus 2 (SARS-CoV-2) (coronavirus disease [COVID-19]) vaccine, mRNA-LNP, spike protein, preservative free, 30 mcg/0.3 mL dosage, diluent reconstituted; first dose | 0 (0.0%) | 0 (0.0%) |  | 0 (0.0%) | 0 (0.0%) |  |
| Immunization administration by intramuscular injection of severe acute respiratory syndrome coronavirus 2 (SARS-CoV-2) (coronavirus disease [COVID-19]) vaccine, mRNA-LNP, spike protein, preservative free, 30 mcg/0.3 mL dosage, diluent reconstituted; second dose | 0 (0.0%) | 0 (0.0%) |  | 0 (0.0%) | 0 (0.0%) |  |
| Severe acute respiratory syndrome coronavirus 2 (SARS-CoV-2) (coronavirus disease [COVID-19]) vaccine, mRNA-LNP, spike protein, preservative free, 100 mcg/0.5 mL dosage, for intramuscular use | 0 (0.0%) | 0 (0.0%) |  | 0 (0.0%) | 0 (0.0%) |  |
| Immunization administration by intramuscular injection of severe acute respiratory syndrome coronavirus 2 (SARS-CoV-2) (coronavirus disease [COVID-19]) vaccine, mRNA-LNP, spike protein, preservative free, 100 mcg/0.5 mL dosage; first dose | 0 (0.0%) | 0 (0.0%) |  | 0 (0.0%) | 0 (0.0%) |  |
| Immunization administration by intramuscular injection of severe acute respiratory syndrome coronavirus 2 (SARS-CoV-2) (coronavirus disease [COVID-19]) vaccine, mRNA-LNP, spike protein, preservative free, 100 mcg/0.5 mL dosage; second dose | 0 (0.0%) | 0 (0.0%) |  | 0 (0.0%) | 0 (0.0%) |  |
| Severe acute respiratory syndrome coronavirus 2 (SARS-CoV-2) (coronavirus disease [COVID-19]) vaccine, DNA, spike protein, chimpanzee adenovirus Oxford 1 (ChAdOx1) vector, preservative free, 5x1010 viral particles/0.5 mL dosage, for intramuscular use | 0 (0.0%) | 0 (0.0%) |  | 0 (0.0%) | 0 (0.0%) |  |
| Immunization administration by intramuscular injection of severe acute respiratory syndrome coronavirus 2 (SARS-CoV-2) (coronavirus disease [COVID-19]) vaccine, DNA, spike protein, chimpanzee adenovirus Oxford 1 (ChAdOx1) vector, preservative free, 5x1010 viral particles/0.5 mL dosage; first dose | 0 (0.0%) | 0 (0.0%) |  | 0 (0.0%) | 0 (0.0%) |  |
| Immunization administration by intramuscular injection of severe acute respiratory syndrome coronavirus 2 (SARS-CoV-2) (coronavirus disease [COVID-19]) vaccine, DNA, spike protein, chimpanzee adenovirus Oxford 1 (ChAdOx1) vector, preservative free, 5x1010 viral particles/0.5 mL dosage; second dose | 0 (0.0%) | 0 (0.0%) |  | 0 (0.0%) | 0 (0.0%) |  |
| Severe acute respiratory syndrome coronavirus 2 (SARS-CoV-2) (coronavirus disease [COVID-19]) vaccine, DNA, spike protein, adenovirus type 26 (Ad26) vector, preservative free, 5x1010 viral particles/0.5 mL dosage, for intramuscular use | 0 (0.0%) | 0 (0.0%) |  | 0 (0.0%) | 0 (0.0%) |  |
| Immunization administration by intramuscular injection of severe acute respiratory syndrome coronavirus 2 (SARS-CoV-2) (coronavirus disease [COVID-19]) vaccine, DNA, spike protein, adenovirus type 26 (Ad26) vector, preservative free, 5x1010 viral particles/0.5 mL dosage, single dose | 0 (0.0%) | 0 (0.0%) |  | 0 (0.0%) | 0 (0.0%) |  |
| Introduction of COVID-19 Vaccine into Subcutaneous Tissue, Percutaneous Approach, New Technology Group 6 | 0 (0.0%) | 0 (0.0%) |  | 0 (0.0%) | 0 (0.0%) |  |
| Introduction of COVID-19 Vaccine Dose 1 into Subcutaneous Tissue, Percutaneous Approach, New Technology Group 6 | 0 (0.0%) | 0 (0.0%) |  | 0 (0.0%) | 0 (0.0%) |  |
| Introduction of COVID-19 Vaccine Dose 2 into Subcutaneous Tissue, Percutaneous Approach, New Technology Group 6 | 0 (0.0%) | 0 (0.0%) |  | 0 (0.0%) | 0 (0.0%) |  |
| Introduction of COVID-19 Vaccine into Muscle, Percutaneous Approach, New Technology Group 6 | 0 (0.0%) | 0 (0.0%) |  | 0 (0.0%) | 0 (0.0%) |  |
| Introduction of COVID-19 Vaccine Dose 1 into Muscle, Percutaneous Approach, New Technology Group 6 | 0 (0.0%) | 0 (0.0%) |  | 0 (0.0%) | 0 (0.0%) |  |
| Introduction of COVID-19 Vaccine Dose 2 into Muscle, Percutaneous Approach, New Technology Group 6 | 0 (0.0%) | 0 (0.0%) |  | 0 (0.0%) | 0 (0.0%) |  |
| SARS-CoV-2 (COVID-19) Vaccine | 10 (0.0%) | 10 (0.0%) | 0.01 | 10 (0.0%) | 0 (0.0%) | 0.02 |

**Table 3. Baseline Characteristics for Block 3 Before and After Matching**

|  | Before matching | | | After matching | | |
| --- | --- | --- | --- | --- | --- | --- |
|  | Cohort, No. (%) | | | Cohort, No. (%) | | |
| Characteristics | COVID-19 | Other RTI | SMD | COVID-19 | Other RTI | SMD |
| Total number | 59570 | 123576 |  | 57474 | 57474 |  |
| Age at index, mean (SD), y | 40.7 (20.0) | 33.7 (24.4) | 0.31 | 40.0 (19.9) | 41.3 (22.4) | 0.06 |
| Current age, mean (SD), y | 43.2 (20.0) | 36.1 (24.3) | 0.32 | 42.5 (19.8) | 43.7 (22.3) | 0.06 |
| Gender | | | | | | |
| Female | 31307 (52.6%) | 67420 (54.6%) | 0.04 | 30419 (52.9%) | 30799 (53.6%) | 0.01 |
| Male | 28225 (47.4%) | 56113 (45.4%) | 0.04 | 27020 (47.0%) | 26639 (46.4%) | 0.01 |
| Unknown | 38 (0.1%) | 43 (0.0%) | 0.01 | 35 (0.1%) | 36 (0.1%) | 0.00 |
| Race | | | | | | |
| White | 32887 (55.2%) | 81288 (65.8%) | 0.22 | 32346 (56.3%) | 33031 (57.5%) | 0.02 |
| Black or African American | 11544 (19.4%) | 18730 (15.2%) | 0.11 | 10977 (19.1%) | 10311 (17.9%) | 0.03 |
| Asian | 1435 (2.4%) | 2844 (2.3%) | 0.01 | 1400 (2.4%) | 1440 (2.5%) | 0.00 |
| American Indian or Alaska  Native | 201 (0.3%) | 353 (0.3%) | 0.01 | 195 (0.3%) | 183 (0.3%) | 0.00 |
| Native Hawaiian or Other  Pacific Islander | 235 (0.4%) | 257 (0.2%) | 0.03 | 203 (0.4%) | 194 (0.3%) | 0.00 |
| Unknown race | 13268 (22.3%) | 20104 (16.3%) | 0.15 | 12353 (21.5%) | 12315 (21.4%) | 0.00 |
| Ethnicity | | | | | | |
| Hispanic or Latino | 10577 (17.8%) | 13966 (11.3%) | 0.18 | 9502 (16.5%) | 8063 (14.0%) | 0.07 |
| Not Hispanic or Latino | 35107 (58.9%) | 84332 (68.2%) | 0.19 | 34548 (60.1%) | 35527 (61.8%) | 0.03 |
| Unknown ethnicity | 13886 (23.3%) | 25278 (20.5%) | 0.07 | 13424 (23.4%) | 13884 (24.2%) | 0.02 |
| Overweight and obesity | 5838 (9.8%) | 11562 (9.4%) | 0.02 | 5670 (9.9%) | 5517 (9.6%) | 0.01 |
| Hypertensive diseases | 10052 (16.9%) | 21640 (17.5%) | 0.02 | 9804 (17.1%) | 9651 (16.8%) | 0.01 |
| Type 1 diabetes mellitus | 546 (0.9%) | 1066 (0.9%) | 0.01 | 520 (0.9%) | 505 (0.9%) | 0.00 |
| Type 2 diabetes mellitus | 5114 (8.6%) | 9152 (7.4%) | 0.04 | 4834 (8.4%) | 4770 (8.3%) | 0.00 |
| Bronchitis, not specified as acute or chronic | 1385 (2.3%) | 5272 (4.3%) | 0.11 | 1383 (2.4%) | 1457 (2.5%) | 0.01 |
| Simple and mucopurulent chronic bronchitis | 95 (0.2%) | 537 (0.4%) | 0.05 | 95 (0.2%) | 120 (0.2%) | 0.01 |
| Unspecified chronic bronchitis | 102 (0.2%) | 589 (0.5%) | 0.05 | 102 (0.2%) | 141 (0.2%) | 0.01 |
| Emphysema | 422 (0.7%) | 2079 (1.7%) | 0.09 | 421 (0.7%) | 503 (0.9%) | 0.02 |
| Other chronic obstructive pulmonary disease | 924 (1.6%) | 4596 (3.7%) | 0.14 | 924 (1.6%) | 1089 (1.9%) | 0.02 |
| Asthma | 3809 (6.4%) | 13924 (11.3%) | 0.17 | 3793 (6.6%) | 3889 (6.8%) | 0.01 |
| Bronchiectasis | 192 (0.3%) | 1033 (0.8%) | 0.07 | 192 (0.3%) | 257 (0.4%) | 0.02 |
| Other forms of heart disease | 5381 (9.0%) | 13744 (11.1%) | 0.07 | 5302 (9.2%) | 5338 (9.3%) | 0.00 |
| Hypertensive chronic kidney disease | 926 (1.6%) | 2051 (1.7%) | 0.01 | 889 (1.5%) | 911 (1.6%) | 0.00 |
| Hepatic failure, not elsewhere classified | 136 (0.2%) | 342 (0.3%) | 0.01 | 136 (0.2%) | 145 (0.3%) | 0.00 |
| Chronic hepatitis, not elsewhere classified | 30 (0.1%) | 77 (0.1%) | 0.01 | 29 (0.1%) | 29 (0.1%) | 0.00 |
| Fibrosis and cirrhosis of liver | 324 (0.5%) | 811 (0.7%) | 0.01 | 317 (0.6%) | 311 (0.5%) | 0.00 |
| Fatty (change of) liver, not elsewhere classified | 1103 (1.9%) | 2053 (1.7%) | 0.01 | 1056 (1.8%) | 999 (1.7%) | 0.01 |
| Chronic passive congestion of liver | 147 (0.2%) | 407 (0.3%) | 0.02 | 143 (0.2%) | 155 (0.3%) | 0.00 |
| Portal hypertension | 137 (0.2%) | 332 (0.3%) | 0.01 | 136 (0.2%) | 135 (0.2%) | 0.00 |
| Other specified diseases of liver | 608 (1.0%) | 1521 (1.2%) | 0.02 | 596 (1.0%) | 584 (1.0%) | 0.00 |
| Cerebral infarction | 879 (1.5%) | 2181 (1.8%) | 0.02 | 848 (1.5%) | 902 (1.6%) | 0.01 |
| Vascular dementia | 65 (0.1%) | 108 (0.1%) | 0.01 | 62 (0.1%) | 62 (0.1%) | 0.00 |
| Dementia in other diseases classified elsewhere | 111 (0.2%) | 199 (0.2%) | 0.01 | 108 (0.2%) | 110 (0.2%) | 0.00 |
| Unspecified dementia | 242 (0.4%) | 427 (0.3%) | 0.01 | 234 (0.4%) | 240 (0.4%) | 0.00 |
| Alzheimer's disease | 109 (0.2%) | 198 (0.2%) | 0.01 | 106 (0.2%) | 108 (0.2%) | 0.00 |
| Frontotemporal dementia | 10 (0.0%) | 10 (0.0%) | 0.01 | 10 (0.0%) | 10 (0.0%) | 0.00 |
| Neurocognitive disorder with Lewy bodies | 10 (0.0%) | 16 (0.0%) | 0.00 | 10 (0.0%) | 10 (0.0%) | 0.00 |
| Neoplasms | 7628 (12.8%) | 18839 (15.2%) | 0.07 | 7527 (13.1%) | 7623 (13.3%) | 0.00 |
| Malignant neoplasms of lymphoid, hematopoietic and related tissue | 396 (0.7%) | 1313 (1.1%) | 0.04 | 394 (0.7%) | 403 (0.7%) | 0.00 |
| Rheumatoid arthritis with rheumatoid factor | 143 (0.2%) | 443 (0.4%) | 0.02 | 143 (0.2%) | 129 (0.2%) | 0.01 |
| Other rheumatoid arthritis | 422 (0.7%) | 1374 (1.1%) | 0.04 | 421 (0.7%) | 433 (0.8%) | 0.00 |
| Systemic lupus erythematosus (SLE) | 194 (0.3%) | 521 (0.4%) | 0.02 | 188 (0.3%) | 200 (0.3%) | 0.00 |
| Psoriasis | 359 (0.6%) | 1028 (0.8%) | 0.03 | 358 (0.6%) | 353 (0.6%) | 0.00 |
| Certain disorders involving the immune mechanism | 695 (1.2%) | 2129 (1.7%) | 0.05 | 671 (1.2%) | 703 (1.2%) | 0.01 |
| Family history of mental and behavioral disorders | 246 (0.4%) | 931 (0.8%) | 0.04 | 244 (0.4%) | 266 (0.5%) | 0.01 |
| Persons with potential health hazards related to socioeconomic and psychosocial circumstances | 950 (1.6%) | 2765 (2.2%) | 0.05 | 940 (1.6%) | 948 (1.6%) | 0.00 |
| Personal history of other mental and behavioral disorders | 111 (0.2%) | 303 (0.2%) | 0.01 | 111 (0.2%) | 110 (0.2%) | 0.00 |
| Nicotine dependence | 2788 (4.7%) | 8567 (6.9%) | 0.10 | 2768 (4.8%) | 2935 (5.1%) | 0.01 |
| Alcohol related disorders | 772 (1.3%) | 1830 (1.5%) | 0.02 | 751 (1.3%) | 741 (1.3%) | 0.00 |
| Other psychoactive substance related disorders | 398 (0.7%) | 1173 (0.9%) | 0.03 | 393 (0.7%) | 388 (0.7%) | 0.00 |
| Cannabis related disorders | 308 (0.5%) | 830 (0.7%) | 0.02 | 303 (0.5%) | 323 (0.6%) | 0.00 |
| Other stimulant related disorders | 144 (0.2%) | 392 (0.3%) | 0.01 | 141 (0.2%) | 152 (0.3%) | 0.00 |
| Opioid related disorders | 153 (0.3%) | 612 (0.5%) | 0.04 | 152 (0.3%) | 157 (0.3%) | 0.00 |
| Cocaine related disorders | 124 (0.2%) | 315 (0.3%) | 0.01 | 122 (0.2%) | 124 (0.2%) | 0.00 |
| Hallucinogen related disorders | 20 (0.0%) | 107 (0.1%) | 0.02 | 20 (0.0%) | 23 (0.0%) | 0.00 |
| Inhalant related disorders | 55 (0.1%) | 172 (0.1%) | 0.01 | 55 (0.1%) | 59 (0.1%) | 0.00 |
| Sedative, hypnotic, or anxiolytic related disorders | 21 (0.0%) | 94 (0.1%) | 0.02 | 21 (0.0%) | 24 (0.0%) | 0.00 |
| Unspecified psychosis not due to a substance or known physiological condition | 62 (0.1%) | 158 (0.1%) | 0.01 | 61 (0.1%) | 60 (0.1%) | 0.00 |
| Schizophrenia | 62 (0.1%) | 141 (0.1%) | 0.00 | 58 (0.1%) | 63 (0.1%) | 0.00 |
| Schizoaffective disorders | 36 (0.1%) | 68 (0.1%) | 0.00 | 34 (0.1%) | 33 (0.1%) | 0.00 |
| Delusional disorders | 19 (0.0%) | 64 (0.1%) | 0.01 | 19 (0.0%) | 21 (0.0%) | 0.00 |
| Brief psychotic disorder | 10 (0.0%) | 14 (0.0%) | 0.00 | 10 (0.0%) | 10 (0.0%) | 0.00 |
| Other psychotic disorder not due to a substance or known physiological condition | 0 (0.0%) | 10 (0.0%) | 0.01 | 0 (0.0%) | 10 (0.0%) | 0.02 |
| Schizotypal disorder | 0 (0.0%) | 0 (0.0%) |  | 0 (0.0%) | 0 (0.0%) |  |
| Shared psychotic disorder | 10 (0.0%) | 10 (0.0%) | 0.01 | 10 (0.0%) | 10 (0.0%) | 0.00 |
| Renal Transplantation Procedures | 86 (0.1%) | 78 (0.1%) | 0.03 | 65 (0.1%) | 65 (0.1%) | 0.00 |
| Liver Transplantation Procedures | 15 (0.0%) | 23 (0.0%) | 0.00 | 15 (0.0%) | 11 (0.0%) | 0.00 |
| Blood Pressure, Systolic | | | | | | |
| <140 mm[Hg] | 21352 (35.8%) | 53094 (43.0%) | 0.15 | 20962 (36.5%) | 20329 (35.4%) | 0.02 |
| 140 - 160 mm[Hg] | 9516 (16.0%) | 20385 (16.5%) | 0.01 | 9244 (16.1%) | 8975 (15.6%) | 0.01 |
| >160[Hg] | 4358 (7.3%) | 9567 (7.7%) | 0.02 | 4230 (7.4%) | 4078 (7.1%) | 0.01 |
| Blood Pressure, Diastolic | | | | | | |
| 0 - 90 mm[Hg] | 22051 (37.0%) | 53304 (43.1%) | 0.13 | 21545 (37.5%) | 20880 (36.3%) | 0.02 |
| 90 - 100 mm[Hg] | 6739 (11.3%) | 14972 (12.1%) | 0.02 | 6581 (11.5%) | 6387 (11.1%) | 0.01 |
| 100 - 0 mm[Hg] | 2746 (4.6%) | 6217 (5.0%) | 0.02 | 2692 (4.7%) | 2621 (4.6%) | 0.01 |
| BMI | | | | | | |
| 0 - 25 kg/m2 | 7473 (12.5%) | 25932 (21.0%) | 0.23 | 7440 (12.9%) | 7381 (12.8%) | 0.00 |
| 25 - 30 kg/m2 | 7080 (11.9%) | 14666 (11.9%) | 0.00 | 6880 (12.0%) | 6966 (12.1%) | 0.00 |
| 30 - 0 kg/m2 | 7206 (12.1%) | 13200 (10.7%) | 0.04 | 6929 (12.1%) | 6769 (11.8%) | 0.01 |
| New or Established Patient | 2996 (5.0%) | 7013 (5.7%) | 0.03 | 2860 (5.0%) | 2722 (4.7%) | 0.01 |
| Hospital Inpatient Services | 3805 (6.4%) | 9104 (7.4%) | 0.04 | 3652 (6.4%) | 3463 (6.0%) | 0.01 |
| Critical Care Services | 931 (1.6%) | 2588 (2.1%) | 0.04 | 912 (1.6%) | 902 (1.6%) | 0.00 |
| Visit: Inpatient Encounter | 11101 (18.6%) | 24063 (19.5%) | 0.02 | 10666 (18.6%) | 10106 (17.6%) | 0.03 |
| Visit: Short Stay | 800 (1.3%) | 1381 (1.1%) | 0.02 | 747 (1.3%) | 731 (1.3%) | 0.00 |
| Visit: Inpatient Non-acute | 42 (0.1%) | 174 (0.1%) | 0.02 | 42 (0.1%) | 47 (0.1%) | 0.00 |
| Severe acute respiratory syndrome coronavirus 2 (SARS-CoV-2) (coronavirus disease [COVID-19]) vaccine, mRNA-LNP, spike protein, preservative free, 30 mcg/0.3 mL dosage, diluent reconstituted, for intramuscular use | 0 (0.0%) | 0 (0.0%) |  | 0 (0.0%) | 0 (0.0%) |  |
| Immunization administration by intramuscular injection of severe acute respiratory syndrome coronavirus 2 (SARS-CoV-2) (coronavirus disease [COVID-19]) vaccine, mRNA-LNP, spike protein, preservative free, 30 mcg/0.3 mL dosage, diluent reconstituted; first dose | 0 (0.0%) | 0 (0.0%) |  | 0 (0.0%) | 0 (0.0%) |  |
| Immunization administration by intramuscular injection of severe acute respiratory syndrome coronavirus 2 (SARS-CoV-2) (coronavirus disease [COVID-19]) vaccine, mRNA-LNP, spike protein, preservative free, 30 mcg/0.3 mL dosage, diluent reconstituted; second dose | 0 (0.0%) | 0 (0.0%) |  | 0 (0.0%) | 0 (0.0%) |  |
| Severe acute respiratory syndrome coronavirus 2 (SARS-CoV-2) (coronavirus disease [COVID-19]) vaccine, mRNA-LNP, spike protein, preservative free, 100 mcg/0.5 mL dosage, for intramuscular use | 0 (0.0%) | 0 (0.0%) |  | 0 (0.0%) | 0 (0.0%) |  |
| Immunization administration by intramuscular injection of severe acute respiratory syndrome coronavirus 2 (SARS-CoV-2) (coronavirus disease [COVID-19]) vaccine, mRNA-LNP, spike protein, preservative free, 100 mcg/0.5 mL dosage; first dose | 0 (0.0%) | 0 (0.0%) |  | 0 (0.0%) | 0 (0.0%) |  |
| Immunization administration by intramuscular injection of severe acute respiratory syndrome coronavirus 2 (SARS-CoV-2) (coronavirus disease [COVID-19]) vaccine, mRNA-LNP, spike protein, preservative free, 100 mcg/0.5 mL dosage; second dose | 0 (0.0%) | 0 (0.0%) |  | 0 (0.0%) | 0 (0.0%) |  |
| Severe acute respiratory syndrome coronavirus 2 (SARS-CoV-2) (coronavirus disease [COVID-19]) vaccine, DNA, spike protein, chimpanzee adenovirus Oxford 1 (ChAdOx1) vector, preservative free, 5x1010 viral particles/0.5 mL dosage, for intramuscular use | 0 (0.0%) | 0 (0.0%) |  | 0 (0.0%) | 0 (0.0%) |  |
| Immunization administration by intramuscular injection of severe acute respiratory syndrome coronavirus 2 (SARS-CoV-2) (coronavirus disease [COVID-19]) vaccine, DNA, spike protein, chimpanzee adenovirus Oxford 1 (ChAdOx1) vector, preservative free, 5x1010 viral particles/0.5 mL dosage; first dose | 0 (0.0%) | 0 (0.0%) |  | 0 (0.0%) | 0 (0.0%) |  |
| Immunization administration by intramuscular injection of severe acute respiratory syndrome coronavirus 2 (SARS-CoV-2) (coronavirus disease [COVID-19]) vaccine, DNA, spike protein, chimpanzee adenovirus Oxford 1 (ChAdOx1) vector, preservative free, 5x1010 viral particles/0.5 mL dosage; second dose | 0 (0.0%) | 0 (0.0%) |  | 0 (0.0%) | 0 (0.0%) |  |
| Severe acute respiratory syndrome coronavirus 2 (SARS-CoV-2) (coronavirus disease [COVID-19]) vaccine, DNA, spike protein, adenovirus type 26 (Ad26) vector, preservative free, 5x1010 viral particles/0.5 mL dosage, for intramuscular use | 0 (0.0%) | 0 (0.0%) |  | 0 (0.0%) | 0 (0.0%) |  |
| Immunization administration by intramuscular injection of severe acute respiratory syndrome coronavirus 2 (SARS-CoV-2) (coronavirus disease [COVID-19]) vaccine, DNA, spike protein, adenovirus type 26 (Ad26) vector, preservative free, 5x1010 viral particles/0.5 mL dosage, single dose | 0 (0.0%) | 0 (0.0%) |  | 0 (0.0%) | 0 (0.0%) |  |
| Introduction of COVID-19 Vaccine into Subcutaneous Tissue, Percutaneous Approach, New Technology Group 6 | 0 (0.0%) | 0 (0.0%) |  | 0 (0.0%) | 0 (0.0%) |  |
| Introduction of COVID-19 Vaccine Dose 1 into Subcutaneous Tissue, Percutaneous Approach, New Technology Group 6 | 0 (0.0%) | 0 (0.0%) |  | 0 (0.0%) | 0 (0.0%) |  |
| Introduction of COVID-19 Vaccine Dose 2 into Subcutaneous Tissue, Percutaneous Approach, New Technology Group 6 | 0 (0.0%) | 0 (0.0%) |  | 0 (0.0%) | 0 (0.0%) |  |
| Introduction of COVID-19 Vaccine into Muscle, Percutaneous Approach, New Technology Group 6 | 0 (0.0%) | 0 (0.0%) |  | 0 (0.0%) | 0 (0.0%) |  |
| Introduction of COVID-19 Vaccine Dose 1 into Muscle, Percutaneous Approach, New Technology Group 6 | 0 (0.0%) | 0 (0.0%) |  | 0 (0.0%) | 0 (0.0%) |  |
| Introduction of COVID-19 Vaccine Dose 2 into Muscle, Percutaneous Approach, New Technology Group 6 | 0 (0.0%) | 0 (0.0%) |  | 0 (0.0%) | 0 (0.0%) |  |
| SARS-CoV-2 (COVID-19) Vaccine | 10 (0.0%) | 10 (0.0%) | 0.01 | 10 (0.0%) | 10 (0.0%) | 0.00 |

**Table 4. Baseline Characteristics for Block 4 Before and After Matching**

|  | Before matching | | | After matching | | |
| --- | --- | --- | --- | --- | --- | --- |
|  | Cohort, No. (%) | | | Cohort, No. (%) | | |
| Characteristics | COVID-19 | Other RTI | SMD | COVID-19 | Other RTI | SMD |
| Total number | 146137 | 176549 |  | 116965 | 116965 |  |
| Age at index, mean (SD), y | 43.9 (20.8) | 32.3 (24.3) | 0.51 | 40.3 (20.3) | 41.6 (22.2) | 0.06 |
| Current age, mean (SD), y | 46.1 (20.7) | 34.5 (24.3) | 0.51 | 42.5 (20.2) | 43.8 (22.2) | 0.06 |
| Gender | | | | | | |
| Female | 74784 (51.2%) | 96566 (54.7%) | 0.07 | 62049 (53.0%) | 64195 (54.9%) | 0.04 |
| Male | 71322 (48.8%) | 79950 (45.3%) | 0.07 | 54891 (46.9%) | 52742 (45.1%) | 0.04 |
| Unknown | 31 (0.0%) | 33 (0.0%) | 0.00 | 25 (0.0%) | 28 (0.0%) | 0.00 |
| Race | | | | | | |
| White | 90546 (62.0%) | 119553 (67.7%) | 0.12 | 75070 (64.2%) | 77324 (66.1%) | 0.04 |
| Black or African American | 25728 (17.6%) | 28094 (15.9%) | 0.05 | 20009 (17.1%) | 18934 (16.2%) | 0.02 |
| Asian | 3807 (2.6%) | 3687 (2.1%) | 0.03 | 2799 (2.4%) | 2787 (2.4%) | 0.00 |
| American Indian or Alaska  Native | 427 (0.3%) | 495 (0.3%) | 0.00 | 335 (0.3%) | 350 (0.3%) | 0.00 |
| Native Hawaiian or Other  Pacific Islander | 248 (0.2%) | 208 (0.1%) | 0.01 | 178 (0.2%) | 170 (0.1%) | 0.00 |
| Unknown race | 25381 (17.4%) | 24512 (13.9%) | 0.10 | 18574 (15.9%) | 17400 (14.9%) | 0.03 |
| Ethnicity | | | | | | |
| Hispanic or Latino | 18862 (12.9%) | 19360 (11.0%) | 0.06 | 13712 (11.7%) | 11871 (10.1%) | 0.05 |
| Not Hispanic or Latino | 92868 (63.5%) | 120899 (68.5%) | 0.10 | 76592 (65.5%) | 78979 (67.5%) | 0.04 |
| Unknown ethnicity | 34407 (23.5%) | 36290 (20.6%) | 0.07 | 26661 (22.8%) | 26115 (22.3%) | 0.01 |
| Overweight and obesity | 18418 (12.6%) | 18465 (10.5%) | 0.07 | 14272 (12.2%) | 14816 (12.7%) | 0.01 |
| Hypertensive diseases | 31518 (21.6%) | 30288 (17.2%) | 0.11 | 24030 (20.5%) | 25169 (21.5%) | 0.02 |
| Type 1 diabetes mellitus | 1786 (1.2%) | 1616 (0.9%) | 0.03 | 1306 (1.1%) | 1318 (1.1%) | 0.00 |
| Type 2 diabetes mellitus | 15258 (10.4%) | 12871 (7.3%) | 0.11 | 10783 (9.2%) | 10875 (9.3%) | 0.00 |
| Bronchitis, not specified as acute or chronic | 4598 (3.1%) | 8212 (4.7%) | 0.08 | 4197 (3.6%) | 4647 (4.0%) | 0.02 |
| Simple and mucopurulent chronic bronchitis | 332 (0.2%) | 678 (0.4%) | 0.03 | 300 (0.3%) | 356 (0.3%) | 0.01 |
| Unspecified chronic bronchitis | 335 (0.2%) | 743 (0.4%) | 0.03 | 317 (0.3%) | 357 (0.3%) | 0.01 |
| Emphysema | 1418 (1.0%) | 2591 (1.5%) | 0.05 | 1298 (1.1%) | 1451 (1.2%) | 0.01 |
| Other chronic obstructive pulmonary disease | 3471 (2.4%) | 5957 (3.4%) | 0.06 | 3159 (2.7%) | 3589 (3.1%) | 0.02 |
| Asthma | 11135 (7.6%) | 21061 (11.9%) | 0.15 | 10216 (8.7%) | 10969 (9.4%) | 0.02 |
| Bronchiectasis | 501 (0.3%) | 1357 (0.8%) | 0.06 | 482 (0.4%) | 610 (0.5%) | 0.02 |
| Other forms of heart disease | 16939 (11.6%) | 18792 (10.6%) | 0.03 | 13391 (11.4%) | 14074 (12.0%) | 0.02 |
| Hypertensive chronic kidney disease | 2868 (2.0%) | 2528 (1.4%) | 0.04 | 2113 (1.8%) | 2097 (1.8%) | 0.00 |
| Hepatic failure, not elsewhere classified | 341 (0.2%) | 390 (0.2%) | 0.00 | 269 (0.2%) | 275 (0.2%) | 0.00 |
| Chronic hepatitis, not elsewhere classified | 98 (0.1%) | 104 (0.1%) | 0.00 | 78 (0.1%) | 78 (0.1%) | 0.00 |
| Fibrosis and cirrhosis of liver | 901 (0.6%) | 962 (0.5%) | 0.01 | 699 (0.6%) | 706 (0.6%) | 0.00 |
| Fatty (change of) liver, not elsewhere classified | 3379 (2.3%) | 3057 (1.7%) | 0.04 | 2511 (2.1%) | 2618 (2.2%) | 0.01 |
| Chronic passive congestion of liver | 450 (0.3%) | 507 (0.3%) | 0.00 | 352 (0.3%) | 389 (0.3%) | 0.01 |
| Portal hypertension | 339 (0.2%) | 323 (0.2%) | 0.01 | 254 (0.2%) | 249 (0.2%) | 0.00 |
| Other specified diseases of liver | 2062 (1.4%) | 2188 (1.2%) | 0.02 | 1634 (1.4%) | 1760 (1.5%) | 0.01 |
| Cerebral infarction | 2498 (1.7%) | 2977 (1.7%) | 0.00 | 1994 (1.7%) | 2072 (1.8%) | 0.01 |
| Vascular dementia | 185 (0.1%) | 126 (0.1%) | 0.02 | 115 (0.1%) | 114 (0.1%) | 0.00 |
| Dementia in other diseases classified elsewhere | 364 (0.2%) | 260 (0.1%) | 0.02 | 239 (0.2%) | 230 (0.2%) | 0.00 |
| Unspecified dementia | 801 (0.5%) | 570 (0.3%) | 0.03 | 530 (0.5%) | 510 (0.4%) | 0.00 |
| Alzheimer's disease | 377 (0.3%) | 244 (0.1%) | 0.03 | 237 (0.2%) | 227 (0.2%) | 0.00 |
| Frontotemporal dementia | 20 (0.0%) | 11 (0.0%) | 0.01 | 12 (0.0%) | 11 (0.0%) | 0.00 |
| Neurocognitive disorder with Lewy bodies | 14 (0.0%) | 20 (0.0%) | 0.00 | 12 (0.0%) | 11 (0.0%) | 0.00 |
| Neoplasms | 22775 (15.6%) | 26332 (14.9%) | 0.02 | 18505 (15.8%) | 19883 (17.0%) | 0.03 |
| Malignant neoplasms of lymphoid, hematopoietic and related tissue | 1124 (0.8%) | 1684 (1.0%) | 0.02 | 999 (0.9%) | 1100 (0.9%) | 0.01 |
| Rheumatoid arthritis with rheumatoid factor | 452 (0.3%) | 552 (0.3%) | 0.00 | 376 (0.3%) | 419 (0.4%) | 0.01 |
| Other rheumatoid arthritis | 1544 (1.1%) | 1867 (1.1%) | 0.00 | 1270 (1.1%) | 1370 (1.2%) | 0.01 |
| Systemic lupus erythematosus (SLE) | 496 (0.3%) | 682 (0.4%) | 0.01 | 448 (0.4%) | 494 (0.4%) | 0.01 |
| Psoriasis | 1223 (0.8%) | 1584 (0.9%) | 0.01 | 1054 (0.9%) | 1148 (1.0%) | 0.01 |
| Certain disorders involving the immune mechanism | 2028 (1.4%) | 3013 (1.7%) | 0.03 | 1734 (1.5%) | 1870 (1.6%) | 0.01 |
| Family history of mental and behavioral disorders | 532 (0.4%) | 1378 (0.8%) | 0.06 | 504 (0.4%) | 543 (0.5%) | 0.00 |
| Persons with potential health hazards related to socioeconomic and psychosocial circumstances | 2427 (1.7%) | 4184 (2.4%) | 0.05 | 2124 (1.8%) | 2172 (1.9%) | 0.00 |
| Personal history of other mental and behavioral disorders | 279 (0.2%) | 426 (0.2%) | 0.01 | 242 (0.2%) | 252 (0.2%) | 0.00 |
| Nicotine dependence | 7521 (5.1%) | 12285 (7.0%) | 0.08 | 6841 (5.8%) | 7711 (6.6%) | 0.03 |
| Alcohol related disorders | 2002 (1.4%) | 2444 (1.4%) | 0.00 | 1663 (1.4%) | 1798 (1.5%) | 0.01 |
| Other psychoactive substance related disorders | 1123 (0.8%) | 1653 (0.9%) | 0.02 | 987 (0.8%) | 1035 (0.9%) | 0.00 |
| Cannabis related disorders | 812 (0.6%) | 1108 (0.6%) | 0.01 | 717 (0.6%) | 772 (0.7%) | 0.01 |
| Other stimulant related disorders | 339 (0.2%) | 590 (0.3%) | 0.02 | 314 (0.3%) | 346 (0.3%) | 0.01 |
| Opioid related disorders | 476 (0.3%) | 829 (0.5%) | 0.02 | 429 (0.4%) | 469 (0.4%) | 0.01 |
| Cocaine related disorders | 352 (0.2%) | 413 (0.2%) | 0.00 | 293 (0.3%) | 316 (0.3%) | 0.00 |
| Hallucinogen related disorders | 52 (0.0%) | 122 (0.1%) | 0.01 | 48 (0.0%) | 61 (0.1%) | 0.01 |
| Inhalant related disorders | 176 (0.1%) | 240 (0.1%) | 0.00 | 156 (0.1%) | 158 (0.1%) | 0.00 |
| Sedative, hypnotic, or anxiolytic related disorders | 60 (0.0%) | 102 (0.1%) | 0.01 | 49 (0.0%) | 59 (0.1%) | 0.00 |
| Unspecified psychosis not due to a substance or known physiological condition | 209 (0.1%) | 229 (0.1%) | 0.00 | 150 (0.1%) | 175 (0.2%) | 0.01 |
| Schizophrenia | 198 (0.1%) | 225 (0.1%) | 0.00 | 146 (0.1%) | 173 (0.1%) | 0.01 |
| Schizoaffective disorders | 77 (0.1%) | 84 (0.0%) | 0.00 | 59 (0.1%) | 66 (0.1%) | 0.00 |
| Delusional disorders | 59 (0.0%) | 72 (0.0%) | 0.00 | 48 (0.0%) | 52 (0.0%) | 0.00 |
| Brief psychotic disorder | 25 (0.0%) | 30 (0.0%) | 0.00 | 18 (0.0%) | 22 (0.0%) | 0.00 |
| Other psychotic disorder not due to a substance or known physiological condition | 10 (0.0%) | 10 (0.0%) | 0.00 | 10 (0.0%) | 10 (0.0%) | 0.00 |
| Schizotypal disorder | 10 (0.0%) | 0 (0.0%) | 0.01 | 10 (0.0%) | 0 (0.0%) | 0.01 |
| Shared psychotic disorder | 0 (0.0%) | 10 (0.0%) | 0.01 | 0 (0.0%) | 10 (0.0%) | 0.01 |
| Renal Transplantation Procedures | 180 (0.1%) | 122 (0.1%) | 0.30 | 127 (0.1%) | 114 (0.1%) | 0.00 |
| Liver Transplantation Procedures | 40 (0.0%) | 40 (0.0%) | 0.17 | 31 (0.0%) | 31 (0.0%) | 0.04 |
| Blood Pressure, Systolic | | | | | | |
| <140 mm[Hg] | 54160 (37.1%) | 80207 (45.4%) | 0.05 | 46432 (39.7%) | 48725 (41.7%) | 0.02 |
| 140 - 160 mm[Hg] | 27298 (18.7%) | 29586 (16.8%) | 0.28 | 21842 (18.7%) | 23315 (19.9%) | 0.03 |
| >160[Hg] | 13307 (9.1%) | 13850 (7.8%) | 0.16 | 10492 (9.0%) | 11033 (9.4%) | 0.04 |
| Blood Pressure, Diastolic | | | | | | |
| 0 - 90 mm[Hg] | 55597 (38.0%) | 80998 (45.9%) | 0.04 | 47332 (40.5%) | 49533 (42.3%) | 0.01 |
| 90 - 100 mm[Hg] | 20029 (13.7%) | 22335 (12.7%) | 0.43 | 16093 (13.8%) | 17230 (14.7%) | 0.05 |
| 100 - 0 mm[Hg] | 8855 (6.1%) | 9250 (5.2%) | 0.17 | 6970 (6.0%) | 7326 (6.3%) | 0.01 |
| BMI | | | | | | |
| 0 - 25 kg/m2 | 20726 (14.2%) | 36150 (20.5%) | 0.13 | 18331 (15.7%) | 18794 (16.1%) | 0.01 |
| 25 - 30 kg/m2 | 21452 (14.7%) | 20233 (11.5%) | 0.02 | 16213 (13.9%) | 17005 (14.5%) | 0.01 |
| 30 - 0 kg/m2 | 21658 (14.8%) | 18704 (10.6%) | 0.03 | 15823 (13.5%) | 16331 (14.0%) | 0.01 |
| New or Established Patient | 7414 (5.1%) | 9675 (5.5%) | 0.03 | 6113 (5.2%) | 6359 (5.4%) | 0.01 |
| Hospital Inpatient Services | 9374 (6.4%) | 12757 (7.2%) | 0.04 | 7787 (6.7%) | 8075 (6.9%) | 0.02 |
| Critical Care Services | 2292 (1.6%) | 3578 (2.0%) | 0.06 | 1976 (1.7%) | 2098 (1.8%) | 0.01 |
| Visit: Inpatient Encounter | 26174 (17.9%) | 34212 (19.4%) | 0.01 | 21449 (18.3%) | 22202 (19.0%) | 0.00 |
| Visit: Short Stay | 2832 (1.9%) | 2052 (1.2%) | 0.02 | 1847 (1.6%) | 1759 (1.5%) | 0.00 |
| Visit: Inpatient Non-acute | 195 (0.1%) | 318 (0.2%) | 0.03 | 153 (0.1%) | 165 (0.1%) | 0.01 |
| Severe acute respiratory syndrome coronavirus 2 (SARS-CoV-2) (coronavirus disease [COVID-19]) vaccine, mRNA-LNP, spike protein, preservative free, 30 mcg/0.3 mL dosage, diluent reconstituted, for intramuscular use | 103 (0.1%) | 26 (0.0%) | 0.03 | 45 (0.0%) | 26 (0.0%) | 0.01 |
| Immunization administration by intramuscular injection of severe acute respiratory syndrome coronavirus 2 (SARS-CoV-2) (coronavirus disease [COVID-19]) vaccine, mRNA-LNP, spike protein, preservative free, 30 mcg/0.3 mL dosage, diluent reconstituted; first dose | 101 (0.1%) | 26 (0.0%) | 0.00 | 44 (0.0%) | 26 (0.0%) | 0.00 |
| Immunization administration by intramuscular injection of severe acute respiratory syndrome coronavirus 2 (SARS-CoV-2) (coronavirus disease [COVID-19]) vaccine, mRNA-LNP, spike protein, preservative free, 30 mcg/0.3 mL dosage, diluent reconstituted; second dose | 0 (0.0%) | 0 (0.0%) | 0.00 | 0 (0.0%) | 0 (0.0%) | 0.00 |
| Severe acute respiratory syndrome coronavirus 2 (SARS-CoV-2) (coronavirus disease [COVID-19]) vaccine, mRNA-LNP, spike protein, preservative free, 100 mcg/0.5 mL dosage, for intramuscular use | 12 (0.0%) | 10 (0.0%) | 0.01 | 10 (0.0%) | 10 (0.0%) | 0.00 |
| Immunization administration by intramuscular injection of severe acute respiratory syndrome coronavirus 2 (SARS-CoV-2) (coronavirus disease [COVID-19]) vaccine, mRNA-LNP, spike protein, preservative free, 100 mcg/0.5 mL dosage; first dose | 24 (0.0%) | 10 (0.0%) |  | 10 (0.0%) | 10 (0.0%) |  |
| Immunization administration by intramuscular injection of severe acute respiratory syndrome coronavirus 2 (SARS-CoV-2) (coronavirus disease [COVID-19]) vaccine, mRNA-LNP, spike protein, preservative free, 100 mcg/0.5 mL dosage; second dose | 0 (0.0%) | 0 (0.0%) |  | 0 (0.0%) | 0 (0.0%) |  |
| Severe acute respiratory syndrome coronavirus 2 (SARS-CoV-2) (coronavirus disease [COVID-19]) vaccine, DNA, spike protein, chimpanzee adenovirus Oxford 1 (ChAdOx1) vector, preservative free, 5x1010 viral particles/0.5 mL dosage, for intramuscular use | 0 (0.0%) | 0 (0.0%) |  | 0 (0.0%) | 0 (0.0%) |  |
| Immunization administration by intramuscular injection of severe acute respiratory syndrome coronavirus 2 (SARS-CoV-2) (coronavirus disease [COVID-19]) vaccine, DNA, spike protein, chimpanzee adenovirus Oxford 1 (ChAdOx1) vector, preservative free, 5x1010 viral particles/0.5 mL dosage; first dose | 0 (0.0%) | 0 (0.0%) |  | 0 (0.0%) | 0 (0.0%) |  |
| Immunization administration by intramuscular injection of severe acute respiratory syndrome coronavirus 2 (SARS-CoV-2) (coronavirus disease [COVID-19]) vaccine, DNA, spike protein, chimpanzee adenovirus Oxford 1 (ChAdOx1) vector, preservative free, 5x1010 viral particles/0.5 mL dosage; second dose | 0 (0.0%) | 0 (0.0%) |  | 0 (0.0%) | 0 (0.0%) |  |
| Severe acute respiratory syndrome coronavirus 2 (SARS-CoV-2) (coronavirus disease [COVID-19]) vaccine, DNA, spike protein, adenovirus type 26 (Ad26) vector, preservative free, 5x1010 viral particles/0.5 mL dosage, for intramuscular use | 0 (0.0%) | 0 (0.0%) |  | 0 (0.0%) | 0 (0.0%) |  |
| Immunization administration by intramuscular injection of severe acute respiratory syndrome coronavirus 2 (SARS-CoV-2) (coronavirus disease [COVID-19]) vaccine, DNA, spike protein, adenovirus type 26 (Ad26) vector, preservative free, 5x1010 viral particles/0.5 mL dosage, single dose | 0 (0.0%) | 0 (0.0%) |  | 0 (0.0%) | 0 (0.0%) |  |
| Introduction of COVID-19 Vaccine into Subcutaneous Tissue, Percutaneous Approach, New Technology Group 6 | 0 (0.0%) | 0 (0.0%) |  | 0 (0.0%) | 0 (0.0%) |  |
| Introduction of COVID-19 Vaccine Dose 1 into Subcutaneous Tissue, Percutaneous Approach, New Technology Group 6 | 0 (0.0%) | 0 (0.0%) |  | 0 (0.0%) | 0 (0.0%) |  |
| Introduction of COVID-19 Vaccine Dose 2 into Subcutaneous Tissue, Percutaneous Approach, New Technology Group 6 | 0 (0.0%) | 0 (0.0%) |  | 0 (0.0%) | 0 (0.0%) |  |
| Introduction of COVID-19 Vaccine into Muscle, Percutaneous Approach, New Technology Group 6 | 0 (0.0%) | 0 (0.0%) |  | 0 (0.0%) | 0 (0.0%) |  |
| Introduction of COVID-19 Vaccine Dose 1 into Muscle, Percutaneous Approach, New Technology Group 6 | 0 (0.0%) | 0 (0.0%) |  | 0 (0.0%) | 0 (0.0%) |  |
| Introduction of COVID-19 Vaccine Dose 2 into Muscle, Percutaneous Approach, New Technology Group 6 | 0 (0.0%) | 0 (0.0%) |  | 0 (0.0%) | 0 (0.0%) |  |
| SARS-CoV-2 (COVID-19) Vaccine | 122 (0.1%) | 30 (0.0%) | 0.03 | 53 (0.0%) | 30 (0.0%) | 0.01 |

**Table 5. Baseline Characteristics for Block 5 Before and After Matching**

|  | Before matching | | | After matching | | |
| --- | --- | --- | --- | --- | --- | --- |
|  | Cohort, No. (%) | | | Cohort, No. (%) | | |
| Characteristics | COVID-19 | Other RTI | SMD | COVID-19 | Other RTI | SMD |
| Total number | 95681 | 153222 |  | 85196 | 85196 |  |
| Age at index, mean (SD), y | 44.8 (21.6) | 32.0 (25.6) | 0.54 | 42.5 (21.3) | 43.8 (23.2) | 0.06 |
| Current age, mean (SD), y | 46.8 (21.6) | 34.0 (25.6) | 0.54 | 44.5 (21.3) | 45.8 (23.2) | 0.06 |
| Gender | | | | | | |
| Female | 48252 (50.4%) | 82316 (53.7%) | 0.07 | 43927 (51.6%) | 45254 (53.1%) | 0.03 |
| Male | 47407 (49.5%) | 70884 (46.3%) | 0.07 | 41251 (48.4%) | 39921 (46.9%) | 0.03 |
| Unknown | 22 (0.0%) | 22 (0.0%) | 0.01 | 18 (0.0%) | 21 (0.0%) | 0.00 |
| Race | | | | | | |
| White | 57663 (60.3%) | 101646 (66.3%) | 0.13 | 52749 (61.9%) | 54599 (64.1%) | 0.04 |
| Black or African American | 19564 (20.4%) | 25680 (16.8%) | 0.09 | 16620 (19.5%) | 15688 (18.4%) | 0.03 |
| Asian | 2848 (3.0%) | 3227 (2.1%) | 0.06 | 2353 (2.8%) | 2183 (2.6%) | 0.01 |
| American Indian or Alaska  Native | 251 (0.3%) | 414 (0.3%) | 0.00 | 226 (0.3%) | 219 (0.3%) | 0.00 |
| Native Hawaiian or Other  Pacific Islander | 156 (0.2%) | 192 (0.1%) | 0.01 | 130 (0.2%) | 121 (0.1%) | 0.00 |
| Unknown race | 15199 (15.9%) | 22063 (14.4%) | 0.04 | 13118 (15.4%) | 12386 (14.5%) | 0.02 |
| Ethnicity | | | | | | |
| Hispanic or Latino | 10954 (11.4%) | 17511 (11.4%) | 0.00 | 9569 (11.2%) | 8266 (9.7%) | 0.05 |
| Not Hispanic or Latino | 62620 (65.4%) | 105158 (68.6%) | 0.07 | 56558 (66.4%) | 58393 (68.5%) | 0.05 |
| Unknown ethnicity | 22107 (23.1%) | 30553 (19.9%) | 0.08 | 19069 (22.4%) | 18537 (21.8%) | 0.02 |
| Overweight and obesity | 13393 (14.0%) | 16727 (10.9%) | 0.09 | 11627 (13.6%) | 11994 (14.1%) | 0.01 |
| Hypertensive diseases | 23093 (24.1%) | 28634 (18.7%) | 0.13 | 20114 (23.6%) | 21011 (24.7%) | 0.02 |
| Type 1 diabetes mellitus | 1315 (1.4%) | 1506 (1.0%) | 0.04 | 1119 (1.3%) | 1116 (1.3%) | 0.00 |
| Type 2 diabetes mellitus | 10975 (11.5%) | 12643 (8.3%) | 0.11 | 9152 (10.7%) | 9250 (10.9%) | 0.00 |
| Bronchitis, not specified as acute or chronic | 2968 (3.1%) | 7131 (4.7%) | 0.08 | 2878 (3.4%) | 3160 (3.7%) | 0.02 |
| Simple and mucopurulent chronic bronchitis | 217 (0.2%) | 703 (0.5%) | 0.04 | 213 (0.3%) | 242 (0.3%) | 0.01 |
| Unspecified chronic bronchitis | 247 (0.3%) | 773 (0.5%) | 0.04 | 243 (0.3%) | 277 (0.3%) | 0.01 |
| Emphysema | 1093 (1.1%) | 2713 (1.8%) | 0.05 | 1047 (1.2%) | 1166 (1.4%) | 0.01 |
| Other chronic obstructive pulmonary disease | 2582 (2.7%) | 6111 (4.0%) | 0.07 | 2522 (3.0%) | 2820 (3.3%) | 0.02 |
| Asthma | 7718 (8.1%) | 19674 (12.8%) | 0.16 | 7493 (8.8%) | 8247 (9.7%) | 0.03 |
| Bronchiectasis | 376 (0.4%) | 1446 (0.9%) | 0.07 | 375 (0.4%) | 431 (0.5%) | 0.01 |
| Other forms of heart disease | 12450 (13.0%) | 18698 (12.2%) | 0.02 | 11208 (13.2%) | 11888 (14.0%) | 0.02 |
| Hypertensive chronic kidney disease | 2168 (2.3%) | 2644 (1.7%) | 0.04 | 1828 (2.1%) | 1878 (2.2%) | 0.00 |
| Hepatic failure, not elsewhere classified | 273 (0.3%) | 406 (0.3%) | 0.00 | 248 (0.3%) | 263 (0.3%) | 0.00 |
| Chronic hepatitis, not elsewhere classified | 60 (0.1%) | 112 (0.1%) | 0.00 | 58 (0.1%) | 69 (0.1%) | 0.00 |
| Fibrosis and cirrhosis of liver | 715 (0.7%) | 888 (0.6%) | 0.02 | 597 (0.7%) | 611 (0.7%) | 0.00 |
| Fatty (change of) liver, not elsewhere classified | 2384 (2.5%) | 2777 (1.8%) | 0.05 | 2022 (2.4%) | 2102 (2.5%) | 0.01 |
| Chronic passive congestion of liver | 355 (0.4%) | 440 (0.3%) | 0.01 | 293 (0.3%) | 314 (0.4%) | 0.00 |
| Portal hypertension | 281 (0.3%) | 315 (0.2%) | 0.02 | 232 (0.3%) | 235 (0.3%) | 0.00 |
| Other specified diseases of liver | 1512 (1.6%) | 2021 (1.3%) | 0.02 | 1314 (1.5%) | 1399 (1.6%) | 0.01 |
| Cerebral infarction | 1734 (1.8%) | 3027 (2.0%) | 0.01 | 1570 (1.8%) | 1703 (2.0%) | 0.01 |
| Vascular dementia | 109 (0.1%) | 132 (0.1%) | 0.01 | 91 (0.1%) | 99 (0.1%) | 0.00 |
| Dementia in other diseases classified elsewhere | 233 (0.2%) | 251 (0.2%) | 0.02 | 199 (0.2%) | 202 (0.2%) | 0.00 |
| Unspecified dementia | 494 (0.5%) | 558 (0.4%) | 0.02 | 411 (0.5%) | 420 (0.5%) | 0.00 |
| Alzheimer's disease | 226 (0.2%) | 239 (0.2%) | 0.02 | 190 (0.2%) | 190 (0.2%) | 0.00 |
| Frontotemporal dementia | 10 (0.0%) | 15 (0.0%) | 0.00 | 10 (0.0%) | 10 (0.0%) | 0.00 |
| Neurocognitive disorder with Lewy bodies | 12 (0.0%) | 16 (0.0%) | 0.00 | 11 (0.0%) | 11 (0.0%) | 0.00 |
| Neoplasms | 15925 (16.6%) | 24895 (16.2%) | 0.01 | 14541 (17.1%) | 15809 (18.6%) | 0.04 |
| Malignant neoplasms of lymphoid, hematopoietic and related tissue | 922 (1.0%) | 1923 (1.3%) | 0.03 | 889 (1.0%) | 1029 (1.2%) | 0.02 |
| Rheumatoid arthritis with rheumatoid factor | 343 (0.4%) | 563 (0.4%) | 0.00 | 321 (0.4%) | 336 (0.4%) | 0.00 |
| Other rheumatoid arthritis | 1063 (1.1%) | 1920 (1.3%) | 0.01 | 979 (1.1%) | 1065 (1.3%) | 0.01 |
| Systemic lupus erythematosus (SLE) | 373 (0.4%) | 618 (0.4%) | 0.00 | 343 (0.4%) | 372 (0.4%) | 0.01 |
| Psoriasis | 900 (0.9%) | 1408 (0.9%) | 0.00 | 827 (1.0%) | 878 (1.0%) | 0.01 |
| Certain disorders involving the immune mechanism | 1644 (1.7%) | 3056 (2.0%) | 0.02 | 1518 (1.8%) | 1622 (1.9%) | 0.01 |
| Family history of mental and behavioral disorders | 321 (0.3%) | 1463 (1.0%) | 0.08 | 319 (0.4%) | 373 (0.4%) | 0.01 |
| Persons with potential health hazards related to socioeconomic and psychosocial circumstances | 1616 (1.7%) | 4169 (2.7%) | 0.07 | 1540 (1.8%) | 1554 (1.8%) | 0.00 |
| Personal history of other mental and behavioral disorders | 198 (0.2%) | 362 (0.2%) | 0.01 | 179 (0.2%) | 189 (0.2%) | 0.00 |
| Nicotine dependence | 4857 (5.1%) | 10861 (7.1%) | 0.08 | 4717 (5.5%) | 5245 (6.2%) | 0.03 |
| Alcohol related disorders | 1518 (1.6%) | 2358 (1.5%) | 0.00 | 1409 (1.7%) | 1501 (1.8%) | 0.01 |
| Other psychoactive substance related disorders | 841 (0.9%) | 1595 (1.0%) | 0.02 | 775 (0.9%) | 877 (1.0%) | 0.01 |
| Cannabis related disorders | 534 (0.6%) | 995 (0.6%) | 0.01 | 510 (0.6%) | 565 (0.7%) | 0.01 |
| Other stimulant related disorders | 215 (0.2%) | 523 (0.3%) | 0.02 | 209 (0.2%) | 256 (0.3%) | 0.01 |
| Opioid related disorders | 373 (0.4%) | 754 (0.5%) | 0.02 | 350 (0.4%) | 394 (0.5%) | 0.01 |
| Cocaine related disorders | 270 (0.3%) | 420 (0.3%) | 0.00 | 235 (0.3%) | 265 (0.3%) | 0.01 |
| Hallucinogen related disorders | 60 (0.1%) | 113 (0.1%) | 0.00 | 58 (0.1%) | 65 (0.1%) | 0.00 |
| Inhalant related disorders | 115 (0.1%) | 217 (0.1%) | 0.01 | 110 (0.1%) | 131 (0.2%) | 0.01 |
| Sedative, hypnotic, or anxiolytic related disorders | 40 (0.0%) | 106 (0.1%) | 0.01 | 40 (0.0%) | 44 (0.1%) | 0.00 |
| Unspecified psychosis not due to a substance or known physiological condition | 150 (0.2%) | 217 (0.1%) | 0.00 | 129 (0.2%) | 139 (0.2%) | 0.00 |
| Schizophrenia | 139 (0.1%) | 202 (0.1%) | 0.00 | 124 (0.1%) | 119 (0.1%) | 0.00 |
| Schizoaffective disorders | 52 (0.1%) | 75 (0.0%) | 0.00 | 48 (0.1%) | 46 (0.1%) | 0.00 |
| Delusional disorders | 64 (0.1%) | 81 (0.1%) | 0.01 | 51 (0.1%) | 56 (0.1%) | 0.00 |
| Brief psychotic disorder | 20 (0.0%) | 17 (0.0%) | 0.01 | 14 (0.0%) | 13 (0.0%) | 0.00 |
| Other psychotic disorder not due to a substance or known physiological condition | 10 (0.0%) | 10 (0.0%) | 0.00 | 10 (0.0%) | 10 (0.0%) | 0.00 |
| Schizotypal disorder | 0 (0.0%) | 0 (0.0%) |  | 0 (0.0%) | 0 (0.0%) |  |
| Shared psychotic disorder | 0 (0.0%) | 10 (0.0%) | 0.01 | 0 (0.0%) | 10 (0.0%) | 0.02 |
| Renal Transplantation Procedures | 161 (0.2%) | 120 (0.1%) | 0.03 | 118 (0.1%) | 104 (0.1%) | 0.00 |
| Liver Transplantation Procedures | 25 (0.0%) | 32 (0.0%) | 0.00 | 21 (0.0%) | 24 (0.0%) | 0.00 |
| Blood Pressure, Systolic | | | | | | |
| <140 mm[Hg] | 38865 (40.6%) | 69626 (45.4%) | 0.10 | 35501 (41.7%) | 36664 (43.0%) | 0.03 |
| 140 - 160 mm[Hg] | 20341 (21.3%) | 26127 (17.1%) | 0.11 | 17732 (20.8%) | 18539 (21.8%) | 0.02 |
| >160[Hg] | 10194 (10.7%) | 12724 (8.3%) | 0.08 | 8802 (10.3%) | 9134 (10.7%) | 0.01 |
| Blood Pressure, Diastolic | | | | | | |
| 0 - 90 mm[Hg] | 40161 (42.0%) | 70284 (45.9%) | 0.08 | 36386 (42.7%) | 37440 (43.9%) | 0.02 |
| 90 - 100 mm[Hg] | 14634 (15.3%) | 19450 (12.7%) | 0.08 | 12835 (15.1%) | 13442 (15.8%) | 0.02 |
| 100 - 0 mm[Hg] | 6487 (6.8%) | 8181 (5.3%) | 0.06 | 5600 (6.6%) | 5830 (6.8%) | 0.01 |
| BMI | | | | | | |
| 0 - 25 kg/m2 | 12866 (13.4%) | 33036 (21.6%) | 0.21 | 12265 (14.4%) | 12824 (15.1%) | 0.02 |
| 25 - 30 kg/m2 | 12709 (13.3%) | 16986 (11.1%) | 0.07 | 11137 (13.1%) | 11799 (13.8%) | 0.02 |
| 30 - 0 kg/m2 | 13768 (14.4%) | 15948 (10.4%) | 0.12 | 11798 (13.8%) | 12039 (14.1%) | 0.01 |
| New or Established Patient | 5944 (6.2%) | 9887 (6.5%) | 0.01 | 5345 (6.3%) | 5711 (6.7%) | 0.02 |
| Hospital Inpatient Services | 7540 (7.9%) | 12989 (8.5%) | 0.02 | 6807 (8.0%) | 7227 (8.5%) | 0.02 |
| Critical Care Services | 1891 (2.0%) | 3708 (2.4%) | 0.03 | 1761 (2.1%) | 1912 (2.2%) | 0.01 |
| Visit: Inpatient Encounter | 19409 (20.3%) | 30920 (20.2%) | 0.00 | 17351 (20.4%) | 18012 (21.1%) | 0.02 |
| Visit: Short Stay | 2298 (2.4%) | 1760 (1.1%) | 0.10 | 1628 (1.9%) | 1455 (1.7%) | 0.02 |
| Visit: Inpatient Non-acute | 113 (0.1%) | 276 (0.2%) | 0.02 | 108 (0.1%) | 105 (0.1%) | 0.00 |
| Severe acute respiratory syndrome coronavirus 2 (SARS-CoV-2) (coronavirus disease [COVID-19]) vaccine, mRNA-LNP, spike protein, preservative free, 30 mcg/0.3 mL dosage, diluent reconstituted, for intramuscular use | 714 (0.7%) | 1469 (1.0%) | 0.02 | 668 (0.8%) | 732 (0.9%) | 0.01 |
| Immunization administration by intramuscular injection of severe acute respiratory syndrome coronavirus 2 (SARS-CoV-2) (coronavirus disease [COVID-19]) vaccine, mRNA-LNP, spike protein, preservative free, 30 mcg/0.3 mL dosage, diluent reconstituted; first dose | 710 (0.7%) | 1459 (1.0%) | 0.02 | 664 (0.8%) | 729 (0.9%) | 0.01 |
| Immunization administration by intramuscular injection of severe acute respiratory syndrome coronavirus 2 (SARS-CoV-2) (coronavirus disease [COVID-19]) vaccine, mRNA-LNP, spike protein, preservative free, 30 mcg/0.3 mL dosage, diluent reconstituted; second dose | 204 (0.2%) | 768 (0.5%) | 0.05 | 204 (0.2%) | 242 (0.3%) | 0.01 |
| Severe acute respiratory syndrome coronavirus 2 (SARS-CoV-2) (coronavirus disease [COVID-19]) vaccine, mRNA-LNP, spike protein, preservative free, 100 mcg/0.5 mL dosage, for intramuscular use | 118 (0.1%) | 557 (0.4%) | 0.05 | 116 (0.1%) | 125 (0.1%) | 0.00 |
| Immunization administration by intramuscular injection of severe acute respiratory syndrome coronavirus 2 (SARS-CoV-2) (coronavirus disease [COVID-19]) vaccine, mRNA-LNP, spike protein, preservative free, 100 mcg/0.5 mL dosage; first dose | 182 (0.2%) | 649 (0.4%) | 0.04 | 177 (0.2%) | 188 (0.2%) | 0.00 |
| Immunization administration by intramuscular injection of severe acute respiratory syndrome coronavirus 2 (SARS-CoV-2) (coronavirus disease [COVID-19]) vaccine, mRNA-LNP, spike protein, preservative free, 100 mcg/0.5 mL dosage; second dose | 43 (0.0%) | 239 (0.2%) | 0.04 | 43 (0.1%) | 43 (0.1%) | 0.00 |
| Severe acute respiratory syndrome coronavirus 2 (SARS-CoV-2) (coronavirus disease [COVID-19]) vaccine, DNA, spike protein, chimpanzee adenovirus Oxford 1 (ChAdOx1) vector, preservative free, 5x1010 viral particles/0.5 mL dosage, for intramuscular use | 0 (0.0%) | 0 (0.0%) |  | 0 (0.0%) | 0 (0.0%) |  |
| Immunization administration by intramuscular injection of severe acute respiratory syndrome coronavirus 2 (SARS-CoV-2) (coronavirus disease [COVID-19]) vaccine, DNA, spike protein, chimpanzee adenovirus Oxford 1 (ChAdOx1) vector, preservative free, 5x1010 viral particles/0.5 mL dosage; first dose | 0 (0.0%) | 0 (0.0%) |  | 0 (0.0%) | 0 (0.0%) |  |
| Immunization administration by intramuscular injection of severe acute respiratory syndrome coronavirus 2 (SARS-CoV-2) (coronavirus disease [COVID-19]) vaccine, DNA, spike protein, chimpanzee adenovirus Oxford 1 (ChAdOx1) vector, preservative free, 5x1010 viral particles/0.5 mL dosage; second dose | 0 (0.0%) | 0 (0.0%) |  | 0 (0.0%) | 0 (0.0%) |  |
| Severe acute respiratory syndrome coronavirus 2 (SARS-CoV-2) (coronavirus disease [COVID-19]) vaccine, DNA, spike protein, adenovirus type 26 (Ad26) vector, preservative free, 5x1010 viral particles/0.5 mL dosage, for intramuscular use | 10 (0.0%) | 10 (0.0%) | 0.00 | 10 (0.0%) | 10 (0.0%) | 0.00 |
| Immunization administration by intramuscular injection of severe acute respiratory syndrome coronavirus 2 (SARS-CoV-2) (coronavirus disease [COVID-19]) vaccine, DNA, spike protein, adenovirus type 26 (Ad26) vector, preservative free, 5x1010 viral particles/0.5 mL dosage, single dose | 10 (0.0%) | 10 (0.0%) | 0.00 | 10 (0.0%) | 10 (0.0%) | 0.00 |
| Introduction of COVID-19 Vaccine into Subcutaneous Tissue, Percutaneous Approach, New Technology Group 6 | 0 (0.0%) | 0 (0.0%) |  | 0 (0.0%) | 0 (0.0%) |  |
| Introduction of COVID-19 Vaccine Dose 1 into Subcutaneous Tissue, Percutaneous Approach, New Technology Group 6 | 0 (0.0%) | 0 (0.0%) |  | 0 (0.0%) | 0 (0.0%) |  |
| Introduction of COVID-19 Vaccine Dose 2 into Subcutaneous Tissue, Percutaneous Approach, New Technology Group 6 | 0 (0.0%) | 0 (0.0%) |  | 0 (0.0%) | 0 (0.0%) |  |
| Introduction of COVID-19 Vaccine into Muscle, Percutaneous Approach, New Technology Group 6 | 0 (0.0%) | 10 (0.0%) | 0.01 | 0 (0.0%) | 0 (0.0%) |  |
| Introduction of COVID-19 Vaccine Dose 1 into Muscle, Percutaneous Approach, New Technology Group 6 | 0 (0.0%) | 0 (0.0%) |  | 0 (0.0%) | 0 (0.0%) |  |
| Introduction of COVID-19 Vaccine Dose 2 into Muscle, Percutaneous Approach, New Technology Group 6 | 0 (0.0%) | 10 (0.0%) | 0.01 | 0 (0.0%) | 0 (0.0%) |  |
| SARS-CoV-2 (COVID-19) Vaccine | 842 (0.9%) | 2313 (1.5%) | 0.06 | 814 (1.0%) | 890 (1.0%) | 0.01 |

**Table 6. Baseline Characteristics for Block 6 Before and After Matching**

|  | Before matching | | | After matching | | |
| --- | --- | --- | --- | --- | --- | --- |
|  | Cohort, No. (%) | | | Cohort, No. (%) | | |
| Characteristics | COVID-19 | Other RTI | SMD | COVID-19 | Other RTI | SMD |
| Total number | 45850 | 195170 |  | 45847 | 45847 |  |
| Age at index, mean (SD), y | 42.0 (21.1) | 27.8 (25.6) | 0.61 | 42.0 (21.1) | 43.6 (23.3) | 0.07 |
| Current age, mean (SD), y | 43.8 (21.0) | 29.5 (25.6) | 0.06 | 43.8 (21.0) | 45.3 (23.3) | 0.07 |
| Gender | | | | | | |
| Female | 23457 (51.2%) | 103362 (53.0%) | 0.04 | 23456 (51.2%) | 24186 (52.8%) | 0.03 |
| Male | 22380 (48.8%) | 91780 (47.0%) | 0.04 | 22378 (48.8%) | 21646 (47.2%) | 0.03 |
| Unknown | 13 (0.0%) | 28 (0.0%) | 0.01 | 13 (0.0%) | 15 (0.0%) | 0.00 |
| Race | | | | | | |
| White | 25794 (56.3%) | 125309 (64.2%) | 0.16 | 25794 (56.3%) | 27096 (59.1%) | 0.06 |
| Black or African American | 11234 (24.5%) | 34232 (17.5%) | 0.17 | 11232 (24.5%) | 10306 (22.5%) | 0.05 |
| Asian | 1082 (2.4%) | 4818 (2.5%) | 0.01 | 1082 (2.4%) | 1038 (2.3%) | 0.01 |
| American Indian or Alaska  Native | 139 (0.3%) | 629 (0.3%) | 0.00 | 139 (0.3%) | 131 (0.3%) | 0.00 |
| Native Hawaiian or Other  Pacific Islander | 87 (0.2%) | 279 (0.1%) | 0.01 | 87 (0.2%) | 87 (0.2%) | 0.00 |
| Unknown race | 7514 (16.4%) | 29903 (15.3%) | 0.03 | 7513 (16.4%) | 7189 (15.7%) | 0.02 |
| Ethnicity | | | | | | |
| Hispanic or Latino | 5363 (11.7%) | 26556 (13.6%) | 0.06 | 5363 (11.7%) | 4723 (10.3%) | 0.04 |
| Not Hispanic or Latino | 30559 (66.7%) | 131057 (67.2%) | 0.01 | 30557 (66.7%) | 31229 (68.1%) | 0.03 |
| Unknown ethnicity | 9928 (21.7%) | 37557 (19.2%) | 0.06 | 9927 (21.7%) | 9895 (21.6%) | 0.00 |
| Overweight and obesity | 6477 (14.1%) | 19731 (10.1%) | 0.12 | 6476 (14.1%) | 6342 (13.8%) | 0.01 |
| Hypertensive diseases | 9662 (21.1%) | 31570 (16.2%) | 0.13 | 9661 (21.1%) | 9840 (21.5%) | 0.01 |
| Type 1 diabetes mellitus | 538 (1.2%) | 1638 (0.8%) | 0.03 | 537 (1.2%) | 533 (1.2%) | 0.00 |
| Type 2 diabetes mellitus | 4558 (9.9%) | 13772 (7.1%) | 0.10 | 4557 (9.9%) | 4436 (9.7%) | 0.01 |
| Bronchitis, not specified as acute or chronic | 1264 (2.8%) | 8583 (4.4%) | 0.09 | 1264 (2.8%) | 1425 (3.1%) | 0.02 |
| Simple and mucopurulent chronic bronchitis | 85 (0.2%) | 766 (0.4%) | 0.04 | 85 (0.2%) | 92 (0.2%) | 0.00 |
| Unspecified chronic bronchitis | 106 (0.2%) | 867 (0.4%) | 0.04 | 106 (0.2%) | 112 (0.2%) | 0.00 |
| Emphysema | 444 (1.0%) | 2866 (1.5%) | 0.05 | 444 (1.0%) | 507 (1.1%) | 0.01 |
| Other chronic obstructive pulmonary disease | 1102 (2.4%) | 6461 (3.3%) | 0.05 | 1102 (2.4%) | 1228 (2.7%) | 0.02 |
| Asthma | 3949 (8.6%) | 24059 (12.3%) | 0.12 | 3949 (8.6%) | 4125 (9.0%) | 0.01 |
| Bronchiectasis | 158 (0.3%) | 1526 (0.8%) | 0.06 | 158 (0.3%) | 190 (0.4%) | 0.01 |
| Other forms of heart disease | 5510 (12.0%) | 20965 (10.7%) | 0.04 | 5509 (12.0%) | 5678 (12.4%) | 0.01 |
| Hypertensive chronic kidney disease | 966 (2.1%) | 2902 (1.5%) | 0.05 | 965 (2.1%) | 893 (1.9%) | 0.01 |
| Hepatic failure, not elsewhere classified | 125 (0.3%) | 414 (0.2%) | 0.01 | 125 (0.3%) | 117 (0.3%) | 0.00 |
| Chronic hepatitis, not elsewhere classified | 27 (0.1%) | 134 (0.1%) | 0.00 | 27 (0.1%) | 22 (0.0%) | 0.00 |
| Fibrosis and cirrhosis of liver | 282 (0.6%) | 1050 (0.5%) | 0.01 | 282 (0.6%) | 284 (0.6%) | 0.00 |
| Fatty (change of) liver, not elsewhere classified | 993 (2.2%) | 3201 (1.6%) | 0.04 | 993 (2.2%) | 1000 (2.2%) | 0.00 |
| Chronic passive congestion of liver | 104 (0.2%) | 521 (0.3%) | 0.01 | 104 (0.2%) | 126 (0.3%) | 0.01 |
| Portal hypertension | 130 (0.3%) | 405 (0.2%) | 0.02 | 130 (0.3%) | 119 (0.3%) | 0.00 |
| Other specified diseases of liver | 600 (1.3%) | 2260 (1.2%) | 0.01 | 600 (1.3%) | 647 (1.4%) | 0.01 |
| Cerebral infarction | 813 (1.8%) | 3489 (1.8%) | 0.00 | 813 (1.8%) | 795 (1.7%) | 0.00 |
| Vascular dementia | 39 (0.1%) | 126 (0.1%) | 0.01 | 39 (0.1%) | 36 (0.1%) | 0.00 |
| Dementia in other diseases classified elsewhere | 63 (0.1%) | 262 (0.1%) | 0.00 | 63 (0.1%) | 64 (0.1%) | 0.00 |
| Unspecified dementia | 152 (0.3%) | 658 (0.3%) | 0.00 | 152 (0.3%) | 176 (0.4%) | 0.01 |
| Alzheimer's disease | 59 (0.1%) | 280 (0.1%) | 0.00 | 59 (0.1%) | 60 (0.1%) | 0.00 |
| Frontotemporal dementia | 10 (0.0%) | 20 (0.0%) | 0.01 | 10 (0.0%) | 10 (0.0%) | 0.00 |
| Neurocognitive disorder with Lewy bodies | 10 (0.0%) | 18 (0.0%) | 0.01 | 10 (0.0%) | 10 (0.0%) | 0.00 |
| Neoplasms | 6815 (14.9%) | 27799 (14.2%) | 0.02 | 6814 (14.9%) | 7221 (15.8%) | 0.02 |
| Malignant neoplasms of lymphoid, hematopoietic and related tissue | 489 (1.1%) | 2041 (1.0%) | 0.00 | 489 (1.1%) | 507 (1.1%) | 0.00 |
| Rheumatoid arthritis with rheumatoid factor | 147 (0.3%) | 619 (0.3%) | 0.00 | 147 (0.3%) | 144 (0.3%) | 0.00 |
| Other rheumatoid arthritis | 447 (1.0%) | 2183 (1.1%) | 0.01 | 447 (1.0%) | 468 (1.0%) | 0.00 |
| Systemic lupus erythematosus (SLE) | 181 (0.4%) | 684 (0.4%) | 0.01 | 181 (0.4%) | 195 (0.4%) | 0.00 |
| Psoriasis | 304 (0.7%) | 1538 (0.8%) | 0.01 | 304 (0.7%) | 342 (0.7%) | 0.01 |
| Certain disorders involving the immune mechanism | 833 (1.8%) | 3563 (1.8%) | 0.00 | 833 (1.8%) | 815 (1.8%) | 0.00 |
| Family history of mental and behavioral disorders | 172 (0.4%) | 2048 (1.0%) | 0.08 | 172 (0.4%) | 183 (0.4%) | 0.00 |
| Persons with potential health hazards related to socioeconomic and psychosocial circumstances | 908 (2.0%) | 5854 (3.0%) | 0.07 | 908 (2.0%) | 907 (2.0%) | 0.00 |
| Personal history of other mental and behavioral disorders | 101 (0.2%) | 447 (0.2%) | 0.00 | 101 (0.2%) | 107 (0.2%) | 0.00 |
| Nicotine dependence | 2821 (6.2%) | 12198 (6.3%) | 0.00 | 2821 (6.2%) | 3064 (6.7%) | 0.02 |
| Alcohol related disorders | 702 (1.5%) | 2488 (1.3%) | 0.02 | 702 (1.5%) | 707 (1.5%) | 0.00 |
| Other psychoactive substance related disorders | 368 (0.8%) | 1866 (1.0%) | 0.02 | 368 (0.8%) | 397 (0.9%) | 0.01 |
| Cannabis related disorders | 388 (0.8%) | 1181 (0.6%) | 0.03 | 388 (0.8%) | 359 (0.8%) | 0.01 |
| Other stimulant related disorders | 108 (0.2%) | 628 (0.3%) | 0.02 | 108 (0.2%) | 110 (0.2%) | 0.00 |
| Opioid related disorders | 226 (0.5%) | 804 (0.4%) | 0.01 | 226 (0.5%) | 236 (0.5%) | 0.00 |
| Cocaine related disorders | 150 (0.3%) | 426 (0.2%) | 0.02 | 150 (0.3%) | 140 (0.3%) | 0.00 |
| Hallucinogen related disorders | 24 (0.1%) | 167 (0.1%) | 0.01 | 24 (0.1%) | 17 (0.0%) | 0.01 |
| Inhalant related disorders | 51 (0.1%) | 230 (0.1%) | 0.00 | 51 (0.1%) | 61 (0.1%) | 0.01 |
| Sedative, hypnotic, or anxiolytic related disorders | 14 (0.0%) | 134 (0.1%) | 0.02 | 14 (0.0%) | 17 (0.0%) | 0.00 |
| Unspecified psychosis not due to a substance or known physiological condition | 65 (0.1%) | 233 (0.1%) | 0.01 | 65 (0.1%) | 77 (0.2%) | 0.01 |
| Schizophrenia | 63 (0.1%) | 221 (0.1%) | 0.01 | 63 (0.1%) | 74 (0.2%) | 0.01 |
| Schizoaffective disorders | 10 (0.0%) | 86 (0.0%) | 0.01 | 10 (0.0%) | 13 (0.0%) | 0.00 |
| Delusional disorders | 19 (0.0%) | 75 (0.0%) | 0.00 | 19 (0.0%) | 14 (0.0%) | 0.01 |
| Brief psychotic disorder | 10 (0.0%) | 32 (0.0%) | 0.00 | 10 (0.0%) | 10 (0.0%) | 0.00 |
| Other psychotic disorder not due to a substance or known physiological condition | 10 (0.0%) | 10 (0.0%) | 0.01 | 10 (0.0%) | 0 (0.0%) | 0.02 |
| Schizotypal disorder | 0 (0.0%) | 10 (0.0%) | 0.01 | 0 (0.0%) | 10 (0.0%) | 0.02 |
| Shared psychotic disorder | 0 (0.0%) | 10 (0.0%) | 0.01 | 0 (0.0%) | 10 (0.0%) | 0.02 |
| Renal Transplantation Procedures | 84 (0.2%) | 119 (0.1%) | 0.04 | 83 (0.2%) | 53 (0.1%) | 0.02 |
| Liver Transplantation Procedures | 19 (0.0%) | 40 (0.0%) | 0.01 | 19 (0.0%) | 14 (0.0%) | 0.01 |
| Blood Pressure, Systolic | | | | | | |
| <140 mm[Hg] | 17114 (37.3%) | 87452 (44.8%) | 0.15 | 17114 (37.3%) | 17621 (38.4%) | 0.02 |
| 140 - 160 mm[Hg] | 8524 (18.6%) | 30253 (15.5%) | 0.08 | 8524 (18.6%) | 8789 (19.2%) | 0.01 |
| >160[Hg] | 4181 (9.1%) | 14675 (7.5%) | 0.06 | 4181 (9.1%) | 4246 (9.3%) | 0.00 |
| Blood Pressure, Diastolic | | | | | | |
| 0 - 90 mm[Hg] | 17541 (38.3%) | 88169 (45.2%) | 0.14 | 17541 (38.3%) | 18033 (39.3%) | 0.02 |
| 90 - 100 mm[Hg] | 6412 (14.0%) | 22742 (11.7%) | 0.07 | 6412 (14.0%) | 6498 (14.2%) | 0.01 |
| 100 - 0 mm[Hg] | 2931 (6.4%) | 9435 (4.8%) | 0.07 | 2931 (6.4%) | 2885 (6.3%) | 0.00 |
| BMI | | | | | | |
| 0 - 25 kg/m2 | 7241 (15.8%) | 44034 (22.6%) | 0.17 | 7241 (15.8%) | 7283 (15.9%) | 0.00 |
| 25 - 30 kg/m2 | 6536 (14.3%) | 19763 (10.1%) | 0.13 | 6536 (14.3%) | 6497 (14.2%) | 0.00 |
| 30 - 0 kg/m2 | 7291 (15.9%) | 18253 (9.4%) | 0.20 | 7291 (15.9%) | 6809 (14.9%) | 0.03 |
| New or Established Patient | 2508 (5.5%) | 11495 (5.9%) | 0.02 | 2507 (5.5%) | 2547 (5.6%) | 0.00 |
| Hospital Inpatient Services | 3227 (7.0%) | 15563 (8.0%) | 0.04 | 3226 (7.0%) | 3246 (7.1%) | 0.00 |
| Critical Care Services | 836 (1.8%) | 4212 (2.2%) | 0.02 | 835 (1.8%) | 827 (1.8%) | 0.00 |
| Visit: Inpatient Encounter | 9322 (20.3%) | 35659 (18.3%) | 0.05 | 9321 (20.3%) | 9190 (20.0%) | 0.01 |
| Visit: Short Stay | 844 (1.8%) | 2134 (1.1%) | 0.06 | 844 (1.8%) | 718 (1.6%) | 0.02 |
| Visit: Inpatient Non-acute | 36 (0.1%) | 405 (0.2%) | 0.03 | 36 (0.1%) | 35 (0.1%) | 0.00 |
| Severe acute respiratory syndrome coronavirus 2 (SARS-CoV-2) (coronavirus disease [COVID-19]) vaccine, mRNA-LNP, spike protein, preservative free, 30 mcg/0.3 mL dosage, diluent reconstituted, for intramuscular use | 1243 (2.7%) | 5927 (3.0%) | 0.02 | 1242 (2.7%) | 1305 (2.8%) | 0.01 |
| Immunization administration by intramuscular injection of severe acute respiratory syndrome coronavirus 2 (SARS-CoV-2) (coronavirus disease [COVID-19]) vaccine, mRNA-LNP, spike protein, preservative free, 30 mcg/0.3 mL dosage, diluent reconstituted; first dose | 1236 (2.7%) | 5897 (3.0%) | 0.02 | 1235 (2.7%) | 1301 (2.8%) | 0.01 |
| Immunization administration by intramuscular injection of severe acute respiratory syndrome coronavirus 2 (SARS-CoV-2) (coronavirus disease [COVID-19]) vaccine, mRNA-LNP, spike protein, preservative free, 30 mcg/0.3 mL dosage, diluent reconstituted; second dose | 832 (1.8%) | 5041 (2.6%) | 0.05 | 832 (1.8%) | 955 (2.1%) | 0.02 |
| Severe acute respiratory syndrome coronavirus 2 (SARS-CoV-2) (coronavirus disease [COVID-19]) vaccine, mRNA-LNP, spike protein, preservative free, 100 mcg/0.5 mL dosage, for intramuscular use | 184 (0.4%) | 1106 (0.6%) | 0.02 | 184 (0.4%) | 205 (0.4%) | 0.01 |
| Immunization administration by intramuscular injection of severe acute respiratory syndrome coronavirus 2 (SARS-CoV-2) (coronavirus disease [COVID-19]) vaccine, mRNA-LNP, spike protein, preservative free, 100 mcg/0.5 mL dosage; first dose | 212 (0.5%) | 1135 (0.6%) | 0.02 | 212 (0.5%) | 231 (0.5%) | 0.01 |
| Immunization administration by intramuscular injection of severe acute respiratory syndrome coronavirus 2 (SARS-CoV-2) (coronavirus disease [COVID-19]) vaccine, mRNA-LNP, spike protein, preservative free, 100 mcg/0.5 mL dosage; second dose | 166 (0.4%) | 1038 (0.5%) | 0.03 | 166 (0.4%) | 183 (0.4%) | 0.01 |
| Severe acute respiratory syndrome coronavirus 2 (SARS-CoV-2) (coronavirus disease [COVID-19]) vaccine, DNA, spike protein, chimpanzee adenovirus Oxford 1 (ChAdOx1) vector, preservative free, 5x1010 viral particles/0.5 mL dosage, for intramuscular use | 0 (0.0%) | 0 (0.0%) |  | 0 (0.0%) | 0 (0.0%) |  |
| Immunization administration by intramuscular injection of severe acute respiratory syndrome coronavirus 2 (SARS-CoV-2) (coronavirus disease [COVID-19]) vaccine, DNA, spike protein, chimpanzee adenovirus Oxford 1 (ChAdOx1) vector, preservative free, 5x1010 viral particles/0.5 mL dosage; first dose | 0 (0.0%) | 0 (0.0%) |  | 0 (0.0%) | 0 (0.0%) |  |
| Immunization administration by intramuscular injection of severe acute respiratory syndrome coronavirus 2 (SARS-CoV-2) (coronavirus disease [COVID-19]) vaccine, DNA, spike protein, chimpanzee adenovirus Oxford 1 (ChAdOx1) vector, preservative free, 5x1010 viral particles/0.5 mL dosage; second dose | 0 (0.0%) | 0 (0.0%) |  | 0 (0.0%) | 0 (0.0%) |  |
| Severe acute respiratory syndrome coronavirus 2 (SARS-CoV-2) (coronavirus disease [COVID-19]) vaccine, DNA, spike protein, adenovirus type 26 (Ad26) vector, preservative free, 5x1010 viral particles/0.5 mL dosage, for intramuscular use | 16 (0.0%) | 142 (0.1%) | 0.02 | 16 (0.0%) | 23 (0.1%) | 0.01 |
| Immunization administration by intramuscular injection of severe acute respiratory syndrome coronavirus 2 (SARS-CoV-2) (coronavirus disease [COVID-19]) vaccine, DNA, spike protein, adenovirus type 26 (Ad26) vector, preservative free, 5x1010 viral particles/0.5 mL dosage, single dose | 16 (0.0%) | 142 (0.1%) | 0.02 | 16 (0.0%) | 23 (0.1%) | 0.01 |
| Introduction of COVID-19 Vaccine into Subcutaneous Tissue, Percutaneous Approach, New Technology Group 6 | 0 (0.0%) | 0 (0.0%) |  | 0 (0.0%) | 0 (0.0%) |  |
| Introduction of COVID-19 Vaccine Dose 1 into Subcutaneous Tissue, Percutaneous Approach, New Technology Group 6 | 0 (0.0%) | 0 (0.0%) |  | 0 (0.0%) | 0 (0.0%) |  |
| Introduction of COVID-19 Vaccine Dose 2 into Subcutaneous Tissue, Percutaneous Approach, New Technology Group 6 | 0 (0.0%) | 0 (0.0%) |  | 0 (0.0%) | 0 (0.0%) |  |
| Introduction of COVID-19 Vaccine into Muscle, Percutaneous Approach, New Technology Group 6 | 10 (0.0%) | 10 (0.0%) | 0.01 | 10 (0.0%) | 10 (0.0%) | 0.00 |
| Introduction of COVID-19 Vaccine Dose 1 into Muscle, Percutaneous Approach, New Technology Group 6 | 10 (0.0%) | 10 (0.0%) | 0.01 | 10 (0.0%) | 0 (0.0%) | 0.02 |
| Introduction of COVID-19 Vaccine Dose 2 into Muscle, Percutaneous Approach, New Technology Group 6 | 10 (0.0%) | 10 (0.0%) | 0.01 | 10 (0.0%) | 10 (0.0%) | 0.00 |
| SARS-CoV-2 (COVID-19) Vaccine | 1359 (3.0%) | 8181 (4.2%) | 0.07 | 1359 (3.0%) | 1486 (3.2%) | 0.02 |

**Table 7. Baseline Characteristics for Block 7 Before and After Matching**

|  | Before matching | | | After matching | | |
| --- | --- | --- | --- | --- | --- | --- |
|  | Cohort, No. (%) | | | Cohort, No. (%) | | |
| Characteristics | COVID-19 | Other RTI | SMD | COVID-19 | Other RTI | SMD |
| Total number | 108006 | 269898 |  | 107609 | 107609 |  |
| Age at index, mean (SD), y | 41.0 (21.8) | 25.4 (24.6) | 0.67 | 40.8 (21.8) | 42.4 (23.9) | 0.07 |
| Current age, mean (SD), y | 42.4 (21.8) | 26.9 (24.6) | 0.67 | 42.3 (21.8) | 43.9 (23.8) | 0.07 |
| Gender | | | | | | |
| Female | 55519 (51.4%) | 143144 (53.0%) | 0.03 | 55360 (51.4%) | 58121 (54.0%) | 0.05 |
| Male | 52463 (48.6%) | 126719 (47.0%) | 0.03 | 52225 (48.5%) | 49466 (46.0%) | 0.05 |
| Unknown | 24 (0.0%) | 35 (0.0%) | 0.01 | 24 (0.0%) | 22 (0.0%) | 0.00 |
| Race | | | | | | |
| White | 66754 (61.8%) | 174249 (64.6%) | 0.06 | 66543 (61.8%) | 68595 (63.7%) | 0.04 |
| Black or African American | 21415 (19.8%) | 43173 (16.0%) | 0.10 | 21305 (19.8%) | 20138 (18.7%) | 0.03 |
| Asian | 2288 (2.1%) | 7249 (2.7%) | 0.04 | 2288 (2.1%) | 2455 (2.3%) | 0.01 |
| American Indian or Alaska  Native | 375 (0.3%) | 836 (0.3%) | 0.01 | 375 (0.3%) | 343 (0.3%) | 0.01 |
| Native Hawaiian or Other  Pacific Islander | 215 (0.2%) | 407 (0.2%) | 0.01 | 214 (0.2%) | 175 (0.2%) | 0.01 |
| Unknown race | 16959 (15.7%) | 43984 (16.3%) | 0.02 | 16884 (15.7%) | 15903 (14.8%) | 0.03 |
| Ethnicity | | | | | | |
| Hispanic or Latino | 12677 (11.7%) | 38501 (14.3%) | 0.08 | 12643 (11.7%) | 10817 (10.1%) | 0.05 |
| Not Hispanic or Latino | 74293 (68.8%) | 186837 (69.2%) | 0.01 | 74034 (68.8%) | 75487 (70.1%) | 0.03 |
| Unknown ethnicity | 21036 (19.5%) | 44560 (16.5%) | 0.08 | 20932 (19.5%) | 21305 (19.8%) | 0.01 |
| Overweight and obesity | 12700 (11.8%) | 25695 (9.5%) | 0.07 | 12677 (11.8%) | 14168 (13.2%) | 0.04 |
| Hypertensive diseases | 20657 (19.1%) | 36030 (13.3%) | 0.16 | 20616 (19.2%) | 23267 (21.6%) | 0.06 |
| Type 1 diabetes mellitus | 1101 (1.0%) | 1953 (0.7%) | 0.03 | 1099 (1.0%) | 1207 (1.1%) | 0.01 |
| Type 2 diabetes mellitus | 9096 (8.4%) | 15517 (5.7%) | 0.10 | 9077 (8.4%) | 9899 (9.2%) | 0.03 |
| Bronchitis, not specified as acute or chronic | 2960 (2.7%) | 10155 (3.8%) | 0.06 | 2960 (2.8%) | 3766 (3.5%) | 0.04 |
| Simple and mucopurulent chronic bronchitis | 234 (0.2%) | 796 (0.3%) | 0.02 | 234 (0.2%) | 304 (0.3%) | 0.01 |
| Unspecified chronic bronchitis | 242 (0.2%) | 870 (0.3%) | 0.02 | 241 (0.2%) | 309 (0.3%) | 0.01 |
| Emphysema | 970 (0.9%) | 3088 (1.1%) | 0.02 | 968 (0.9%) | 1231 (1.1%) | 0.02 |
| Other chronic obstructive pulmonary disease | 2294 (2.1%) | 6791 (2.5%) | 0.03 | 2294 (2.1%) | 2912 (2.7%) | 0.04 |
| Asthma | 8871 (8.2%) | 31162 (11.5%) | 0.11 | 8867 (8.2%) | 10443 (9.7%) | 0.05 |
| Bronchiectasis | 387 (0.4%) | 1510 (0.6%) | 0.03 | 387 (0.4%) | 478 (0.4%) | 0.01 |
| Other forms of heart disease | 12212 (11.3%) | 24312 (9.0%) | 0.08 | 12176 (11.3%) | 13700 (12.7%) | 0.04 |
| Hypertensive chronic kidney disease | 1896 (1.8%) | 3036 (1.1%) | 0.05 | 1888 (1.8%) | 1989 (1.8%) | 0.01 |
| Hepatic failure, not elsewhere classified | 244 (0.2%) | 432 (0.2%) | 0.02 | 241 (0.2%) | 269 (0.3%) | 0.01 |
| Chronic hepatitis, not elsewhere classified | 64 (0.1%) | 137 (0.1%) | 0.00 | 64 (0.1%) | 78 (0.1%) | 0.01 |
| Fibrosis and cirrhosis of liver | 559 (0.5%) | 1063 (0.4%) | 0.02 | 554 (0.5%) | 619 (0.6%) | 0.01 |
| Fatty (change of) liver, not elsewhere classified | 2200 (2.0%) | 3838 (1.4%) | 0.05 | 2197 (2.0%) | 2449 (2.3%) | 0.02 |
| Chronic passive congestion of liver | 258 (0.2%) | 606 (0.2%) | 0.00 | 257 (0.2%) | 338 (0.3%) | 0.01 |
| Portal hypertension | 219 (0.2%) | 360 (0.1%) | 0.02 | 218 (0.2%) | 228 (0.2%) | 0.00 |
| Other specified diseases of liver | 1275 (1.2%) | 2602 (1.0%) | 0.02 | 1272 (1.2%) | 1504 (1.4%) | 0.02 |
| Cerebral infarction | 1712 (1.6%) | 3887 (1.4%) | 0.01 | 1707 (1.6%) | 1960 (1.8%) | 0.02 |
| Vascular dementia | 73 (0.1%) | 144 (0.1%) | 0.01 | 73 (0.1%) | 90 (0.1%) | 0.01 |
| Dementia in other diseases classified elsewhere | 146 (0.1%) | 296 (0.1%) | 0.01 | 146 (0.1%) | 168 (0.2%) | 0.01 |
| Unspecified dementia | 304 (0.3%) | 659 (0.2%) | 0.01 | 303 (0.3%) | 349 (0.3%) | 0.01 |
| Alzheimer's disease | 143 (0.1%) | 275 (0.1%) | 0.01 | 143 (0.1%) | 160 (0.1%) | 0.00 |
| Frontotemporal dementia | 10 (0.0%) | 23 (0.0%) | 0.00 | 10 (0.0%) | 15 (0.0%) | 0.00 |
| Neurocognitive disorder with Lewy bodies | 13 (0.0%) | 22 (0.0%) | 0.00 | 12 (0.0%) | 14 (0.0%) | 0.00 |
| Neoplasms | 15385 (14.2%) | 33649 (12.5%) | 0.05 | 15365 (14.3%) | 17892 (16.6%) | 0.07 |
| Malignant neoplasms of lymphoid, hematopoietic and related tissue | 908 (0.8%) | 2173 (0.8%) | 0.00 | 907 (0.8%) | 1152 (1.1%) | 0.02 |
| Rheumatoid arthritis with rheumatoid factor | 370 (0.3%) | 678 (0.3%) | 0.02 | 370 (0.3%) | 418 (0.4%) | 0.01 |
| Other rheumatoid arthritis | 1122 (1.0%) | 2429 (0.9%) | 0.01 | 1121 (1.0%) | 1314 (1.2%) | 0.02 |
| Systemic lupus erythematosus (SLE) | 427 (0.4%) | 752 (0.3%) | 0.02 | 427 (0.4%) | 477 (0.4%) | 0.01 |
| Psoriasis | 838 (0.8%) | 1874 (0.7%) | 0.01 | 838 (0.8%) | 990 (0.9%) | 0.02 |
| Certain disorders involving the immune mechanism | 1808 (1.7%) | 3807 (1.4%) | 0.02 | 1800 (1.7%) | 1967 (1.8%) | 0.01 |
| Family history of mental and behavioral disorders | 433 (0.4%) | 2593 (1.0%) | 0.07 | 433 (0.4%) | 510 (0.5%) | 0.01 |
| Persons with potential health hazards related to socioeconomic and psychosocial circumstances | 1793 (1.7%) | 7443 (2.8%) | 0.07 | 1792 (1.7%) | 1951 (1.8%) | 0.01 |
| Personal history of other mental and behavioral disorders | 183 (0.2%) | 640 (0.2%) | 0.02 | 183 (0.2%) | 207 (0.2%) | 0.01 |
| Nicotine dependence | 5785 (5.4%) | 14004 (5.2%) | 0.01 | 5780 (5.4%) | 6909 (6.4%) | 0.04 |
| Alcohol related disorders | 1277 (1.2%) | 2866 (1.1%) | 0.01 | 1272 (1.2%) | 1493 (1.4%) | 0.02 |
| Other psychoactive substance related disorders | 783 (0.7%) | 2064 (0.8%) | 0.00 | 782 (0.7%) | 906 (0.8%) | 0.01 |
| Cannabis related disorders | 758 (0.7%) | 1334 (0.5%) | 0.03 | 755 (0.7%) | 840 (0.8%) | 0.01 |
| Other stimulant related disorders | 280 (0.3%) | 617 (0.2%) | 0.01 | 277 (0.3%) | 302 (0.3%) | 0.00 |
| Opioid related disorders | 422 (0.4%) | 880 (0.3%) | 0.01 | 420 (0.4%) | 478 (0.4%) | 0.01 |
| Cocaine related disorders | 272 (0.3%) | 478 (0.2%) | 0.02 | 270 (0.3%) | 304 (0.3%) | 0.01 |
| Hallucinogen related disorders | 44 (0.0%) | 136 (0.1%) | 0.00 | 44 (0.0%) | 51 (0.0%) | 0.00 |
| Inhalant related disorders | 131 (0.1%) | 263 (0.1%) | 0.01 | 130 (0.1%) | 151 (0.1%) | 0.01 |
| Sedative, hypnotic, or anxiolytic related disorders | 60 (0.1%) | 158 (0.1%) | 0.00 | 60 (0.1%) | 77 (0.1%) | 0.01 |
| Unspecified psychosis not due to a substance or known physiological condition | 126 (0.1%) | 257 (0.1%) | 0.01 | 126 (0.1%) | 148 (0.1%) | 0.01 |
| Schizophrenia | 98 (0.1%) | 222 (0.1%) | 0.00 | 98 (0.1%) | 110 (0.1%) | 0.00 |
| Schizoaffective disorders | 38 (0.0%) | 79 (0.0%) | 0.00 | 38 (0.0%) | 49 (0.0%) | 0.01 |
| Delusional disorders | 46 (0.0%) | 76 (0.0%) | 0.01 | 45 (0.0%) | 49 (0.0%) | 0.00 |
| Brief psychotic disorder | 19 (0.0%) | 31 (0.0%) | 0.01 | 19 (0.0%) | 17 (0.0%) | 0.00 |
| Other psychotic disorder not due to a substance or known physiological condition | 10 (0.0%) | 10 (0.0%) | 0.01 | 10 (0.0%) | 10 (0.0%) | 0.00 |
| Schizotypal disorder | 10 (0.0%) | 10 (0.0%) | 0.01 | 10 (0.0%) | 0 (0.0%) | 0.01 |
| Shared psychotic disorder | 0 (0.0%) | 10 (0.0%) | 0.01 | 0 (0.0%) | 10 (0.0%) | 0.01 |
| Renal Transplantation Procedures | 217 (0.2%) | 148 (0.1%) | 0.04 | 213 (0.2%) | 129 (0.1%) | 0.02 |
| Liver Transplantation Procedures | 33 (0.0%) | 53 (0.0%) | 0.01 | 32 (0.0%) | 32 (0.0%) | 0.00 |
| Blood Pressure, Systolic | | | | | | |
| <140 mm[Hg] | 42214 (39.1%) | 113563 (42.1%) | 0.06 | 42120 (39.1%) | 46207 (42.9%) | 0.08 |
| 140 - 160 mm[Hg] | 19962 (18.5%) | 35621 (13.2%) | 0.15 | 19902 (18.5%) | 22036 (20.5%) | 0.05 |
| >160[Hg] | 9576 (8.9%) | 16731 (6.2%) | 0.10 | 9549 (8.9%) | 10589 (9.8%) | 0.03 |
| Blood Pressure, Diastolic | | | | | | |
| 0 - 90 mm[Hg] | 43360 (40.1%) | 114789 (42.5%) | 0.05 | 43258 (40.2%) | 47260 (43.9%) | 0.08 |
| 90 - 100 mm[Hg] | 14524 (13.4%) | 27176 (10.1%) | 0.11 | 14480 (13.5%) | 16158 (15.0%) | 0.04 |
| 100 - 0 mm[Hg] | 6199 (5.7%) | 11175 (4.1%) | 0.07 | 6179 (5.7%) | 6797 (6.3%) | 0.02 |
| BMI | | | | | | |
| 0 - 25 kg/m2 | 15776 (14.6%) | 63065 (23.4%) | 0.22 | 15758 (14.6%) | 16723 (15.5%) | 0.03 |
| 25 - 30 kg/m2 | 13555 (12.6%) | 24765 (9.2%) | 0.11 | 13504 (12.5%) | 14900 (13.8%) | 0.04 |
| 30 - 0 kg/m2 | 13862 (12.8%) | 22890 (8.5%) | 0.14 | 13811 (12.8%) | 15020 (14.0%) | 0.03 |
| New or Established Patient | 6456 (6.0%) | 13228 (4.9%) | 0.05 | 6426 (6.0%) | 6711 (6.2%) | 0.01 |
| Hospital Inpatient Services | 8357 (7.7%) | 18582 (6.9%) | 0.03 | 8324 (7.7%) | 8631 (8.0%) | 0.01 |
| Critical Care Services | 2170 (2.0%) | 4713 (1.7%) | 0.02 | 2162 (2.0%) | 2317 (2.2%) | 0.01 |
| Visit: Inpatient Encounter | 21341 (19.8%) | 46285 (17.1%) | 0.07 | 21257 (19.8%) | 22012 (20.5%) | 0.02 |
| Visit: Short Stay | 1434 (1.3%) | 2695 (1.0%) | 0.03 | 1428 (1.3%) | 1507 (1.4%) | 0.01 |
| Visit: Inpatient Non-acute | 104 (0.1%) | 437 (0.2%) | 0.02 | 104 (0.1%) | 142 (0.1%) | 0.01 |
| Severe acute respiratory syndrome coronavirus 2 (SARS-CoV-2) (coronavirus disease [COVID-19]) vaccine, mRNA-LNP, spike protein, preservative free, 30 mcg/0.3 mL dosage, diluent reconstituted, for intramuscular use | 4461 (4.1%) | 8777 (3.3%) | 0.05 | 4455 (4.1%) | 4993 (4.6%) | 0.02 |
| Immunization administration by intramuscular injection of severe acute respiratory syndrome coronavirus 2 (SARS-CoV-2) (coronavirus disease [COVID-19]) vaccine, mRNA-LNP, spike protein, preservative free, 30 mcg/0.3 mL dosage, diluent reconstituted; first dose | 4379 (4.1%) | 8569 (3.2%) | 0.05 | 4373 (4.1%) | 4899 (4.6%) | 0.02 |
| Immunization administration by intramuscular injection of severe acute respiratory syndrome coronavirus 2 (SARS-CoV-2) (coronavirus disease [COVID-19]) vaccine, mRNA-LNP, spike protein, preservative free, 30 mcg/0.3 mL dosage, diluent reconstituted; second dose | 4118 (3.8%) | 7950 (2.9%) | 0.05 | 4111 (3.8%) | 4616 (4.3%) | 0.02 |
| Severe acute respiratory syndrome coronavirus 2 (SARS-CoV-2) (coronavirus disease [COVID-19]) vaccine, mRNA-LNP, spike protein, preservative free, 100 mcg/0.5 mL dosage, for intramuscular use | 590 (0.5%) | 1422 (0.5%) | 0.00 | 590 (0.5%) | 713 (0.7%) | 0.01 |
| Immunization administration by intramuscular injection of severe acute respiratory syndrome coronavirus 2 (SARS-CoV-2) (coronavirus disease [COVID-19]) vaccine, mRNA-LNP, spike protein, preservative free, 100 mcg/0.5 mL dosage; first dose | 595 (0.6%) | 1401 (0.5%) | 0.00 | 595 (0.6%) | 707 (0.7%) | 0.01 |
| Immunization administration by intramuscular injection of severe acute respiratory syndrome coronavirus 2 (SARS-CoV-2) (coronavirus disease [COVID-19]) vaccine, mRNA-LNP, spike protein, preservative free, 100 mcg/0.5 mL dosage; second dose | 530 (0.5%) | 1350 (0.5%) | 0.00 | 530 (0.5%) | 660 (0.6%) | 0.02 |
| Severe acute respiratory syndrome coronavirus 2 (SARS-CoV-2) (coronavirus disease [COVID-19]) vaccine, DNA, spike protein, chimpanzee adenovirus Oxford 1 (ChAdOx1) vector, preservative free, 5x1010 viral particles/0.5 mL dosage, for intramuscular use | 0 (0.0%) | 0 (0.0%) |  | 0 (0.0%) | 0 (0.0%) |  |
| Immunization administration by intramuscular injection of severe acute respiratory syndrome coronavirus 2 (SARS-CoV-2) (coronavirus disease [COVID-19]) vaccine, DNA, spike protein, chimpanzee adenovirus Oxford 1 (ChAdOx1) vector, preservative free, 5x1010 viral particles/0.5 mL dosage; first dose | 0 (0.0%) | 0 (0.0%) |  | 0 (0.0%) | 0 (0.0%) |  |
| Immunization administration by intramuscular injection of severe acute respiratory syndrome coronavirus 2 (SARS-CoV-2) (coronavirus disease [COVID-19]) vaccine, DNA, spike protein, chimpanzee adenovirus Oxford 1 (ChAdOx1) vector, preservative free, 5x1010 viral particles/0.5 mL dosage; second dose | 0 (0.0%) | 0 (0.0%) |  | 0 (0.0%) | 0 (0.0%) |  |
| Severe acute respiratory syndrome coronavirus 2 (SARS-CoV-2) (coronavirus disease [COVID-19]) vaccine, DNA, spike protein, adenovirus type 26 (Ad26) vector, preservative free, 5x1010 viral particles/0.5 mL dosage, for intramuscular use | 59 (0.1%) | 187 (0.1%) | 0.01 | 59 (0.1%) | 73 (0.1%) | 0.01 |
| Immunization administration by intramuscular injection of severe acute respiratory syndrome coronavirus 2 (SARS-CoV-2) (coronavirus disease [COVID-19]) vaccine, DNA, spike protein, adenovirus type 26 (Ad26) vector, preservative free, 5x1010 viral particles/0.5 mL dosage, single dose | 58 (0.1%) | 187 (0.1%) | 0.01 | 58 (0.1%) | 73 (0.1%) | 0.01 |
| Introduction of COVID-19 Vaccine into Subcutaneous Tissue, Percutaneous Approach, New Technology Group 6 | 0 (0.0%) | 0 (0.0%) |  | 0 (0.0%) | 0 (0.0%) |  |
| Introduction of COVID-19 Vaccine Dose 1 into Subcutaneous Tissue, Percutaneous Approach, New Technology Group 6 | 0 (0.0%) | 0 (0.0%) |  | 0 (0.0%) | 0 (0.0%) |  |
| Introduction of COVID-19 Vaccine Dose 2 into Subcutaneous Tissue, Percutaneous Approach, New Technology Group 6 | 0 (0.0%) | 0 (0.0%) |  | 0 (0.0%) | 0 (0.0%) |  |
| Introduction of COVID-19 Vaccine into Muscle, Percutaneous Approach, New Technology Group 6 | 10 (0.0%) | 10 (0.0%) | 0.01 | 10 (0.0%) | 10 (0.0%) | 0.00 |
| Introduction of COVID-19 Vaccine Dose 1 into Muscle, Percutaneous Approach, New Technology Group 6 | 10 (0.0%) | 15 (0.0%) | 0.00 | 10 (0.0%) | 10 (0.0%) | 0.00 |
| Introduction of COVID-19 Vaccine Dose 2 into Muscle, Percutaneous Approach, New Technology Group 6 | 0 (0.0%) | 10 (0.0%) | 0.01 | 0 (0.0%) | 10 (0.0%) | 0.01 |
| SARS-CoV-2 (COVID-19) Vaccine | 4781 (4.4%) | 11165 (4.1%) | 0.01 | 4776 (4.4%) | 5391 (5.0%) | 0.03 |

**Table 8. Baseline Characteristics for Block 8 Before and After Matching**

|  | Before matching | | | After matching | | |
| --- | --- | --- | --- | --- | --- | --- |
|  | Cohort, No. (%) | | | Cohort, No. (%) | | |
| Characteristics | COVID-19 | Other RTI | SMD | COVID-19 | Other RTI | SMD |
| Total number | 190195 | 312470 |  | 173555 | 173555 |  |
| Age at index, mean (SD), y | 40.0 (22.0) | 26.7 (24.7) | 0.57 | 38.3 (21.7) | 40.1 (23.9) | 0.08 |
| Current age, mean (SD), y | 41.2 (22.0) | 27.9 (24.7) | 0.57 | 39.5 (21.7) | 41.3 (23.9) | 0.08 |
| Gender | | | | | | |
| Female | 99290 (52.2%) | 165515 (53.0%) | 0.02 | 91412 (52.7%) | 94703 (54.6%) | 0.04 |
| Male | 90860 (47.8%) | 146906 (47.0%) | 0.02 | 82105 (47.3%) | 78809 (45.4%) | 0.04 |
| Unknown | 45 (0.0%) | 49 (0.0%) | 0.01 | 38 (0.0%) | 43 (0.0%) | 0.00 |
| Race | | | | | | |
| White | 116179 (61.1%) | 207946 (66.5%) | 0.11 | 107635 (62.0%) | 112592 (64.9%) | 0.06 |
| Black or African American | 39464 (20.7%) | 44900 (14.4%) | 0.17 | 34125 (19.7%) | 30518 (17.6%) | 0.05 |
| Asian | 4600 (2.4%) | 8687 (2.8%) | 0.02 | 4342 (2.5%) | 4565 (2.6%) | 0.01 |
| American Indian or Alaska Native | 564 (0.3%) | 1019 (0.3%) | 0.01 | 518 (0.3%) | 553 (0.3%) | 0.00 |
| Native Hawaiian or Other Pacific  Islander | 293 (0.2%) | 517 (0.2%) | 0.00 | 279 (0.2%) | 293 (0.2%) | 0.00 |
| Unknown race | 29095 (15.3%) | 49401 (15.8%) | 0.01 | 26656 (15.4%) | 25034 (14.4%) | 0.03 |
| Ethnicity | | | | | | |
| Hispanic or Latino | 19758 (10.4%) | 42064 (13.5%) | 0.09 | 18557 (10.7%) | 17178 (9.9%) | 0.03 |
| Not Hispanic or Latino | 135014 (71.0%) | 217750 (69.7%) | 0.03 | 122960 (70.8%) | 123830 (71.3%) | 0.01 |
| Unknown ethnicity | 35423 (18.6%) | 52656 (16.9%) | 0.05 | 32038 (18.5%) | 32547 (18.8%) | 0.01 |
| Overweight and obesity | 24949 (13.1%) | 30179 (9.7%) | 0.11 | 22153 (12.8%) | 24136 (13.9%) | 0.03 |
| Hypertensive diseases | 37739 (19.8%) | 43222 (13.8%) | 0.16 | 33529 (19.3%) | 37203 (21.4%) | 0.05 |
| Type 1 diabetes mellitus | 2077 (1.1%) | 2197 (0.7%) | 0.04 | 1792 (1.0%) | 1817 (1.0%) | 0.00 |
| Type 2 diabetes mellitus | 16357 (8.6%) | 18152 (5.8%) | 0.11 | 14346 (8.3%) | 15431 (8.9%) | 0.02 |
| Bronchitis, not specified as acute or chronic | 6158 (3.2%) | 11675 (3.7%) | 0.03 | 5828 (3.4%) | 7055 (4.1%) | 0.04 |
| Simple and mucopurulent chronic bronchitis | 378 (0.2%) | 902 (0.3%) | 0.02 | 363 (0.2%) | 483 (0.3%) | 0.01 |
| Unspecified chronic bronchitis | 444 (0.2%) | 1012 (0.3%) | 0.02 | 429 (0.2%) | 537 (0.3%) | 0.01 |
| Emphysema | 1923 (1.0%) | 3279 (1.0%) | 0.00 | 1820 (1.0%) | 2200 (1.3%) | 0.02 |
| Other chronic obstructive pulmonary disease | 4528 (2.4%) | 7464 (2.4%) | 0.00 | 4262 (2.5%) | 5233 (3.0%) | 0.03 |
| Asthma | 18112 (9.5%) | 35396 (11.3%) | 0.06 | 17090 (9.8%) | 19076 (11.0%) | 0.04 |
| Bronchiectasis | 621 (0.3%) | 1593 (0.5%) | 0.03 | 611 (0.4%) | 783 (0.5%) | 0.02 |
| Other forms of heart disease | 22074 (11.6%) | 27079 (8.7%) | 0.10 | 19621 (11.3%) | 21304 (12.3%) | 0.03 |
| Hypertensive chronic kidney disease | 3414 (1.8%) | 3121 (1.0%) | 0.07 | 2885 (1.7%) | 2816 (1.6%) | 0.00 |
| Hepatic failure, not elsewhere classified | 427 (0.2%) | 465 (0.1%) | 0.02 | 372 (0.2%) | 386 (0.2%) | 0.00 |
| Chronic hepatitis, not elsewhere classified | 111 (0.1%) | 118 (0.0%) | 0.01 | 100 (0.1%) | 97 (0.1%) | 0.00 |
| Fibrosis and cirrhosis of liver | 1047 (0.6%) | 1231 (0.4%) | 0.02 | 932 (0.5%) | 982 (0.6%) | 0.00 |
| Fatty (change of) liver, not elsewhere classified | 4298 (2.3%) | 4658 (1.5%) | 0.06 | 3809 (2.2%) | 4107 (2.4%) | 0.01 |
| Chronic passive congestion of liver | 505 (0.3%) | 647 (0.2%) | 0.01 | 457 (0.3%) | 523 (0.3%) | 0.01 |
| Portal hypertension | 405 (0.2%) | 424 (0.1%) | 0.02 | 365 (0.2%) | 369 (0.2%) | 0.00 |
| Other specified diseases of liver | 2522 (1.3%) | 3020 (1.0%) | 0.03 | 2267 (1.3%) | 2549 (1.5%) | 0.01 |
| Cerebral infarction | 3003 (1.6%) | 4206 (1.3%) | 0.02 | 2702 (1.6%) | 3019 (1.7%) | 0.01 |
| Vascular dementia | 159 (0.1%) | 145 (0.0%) | 0.01 | 135 (0.1%) | 125 (0.1%) | 0.00 |
| Dementia in other diseases classified elsewhere | 319 (0.2%) | 341 (0.1%) | 0.02 | 269 (0.2%) | 302 (0.2%) | 0.00 |
| Unspecified dementia | 716 (0.4%) | 671 (0.2%) | 0.03 | 601 (0.3%) | 591 (0.3%) | 0.00 |
| Alzheimer's disease | 296 (0.2%) | 306 (0.1%) | 0.02 | 247 (0.1%) | 274 (0.2%) | 0.00 |
| Frontotemporal dementia | 18 (0.0%) | 20 (0.0%) | 0.00 | 14 (0.0%) | 17 (0.0%) | 0.00 |
| Neurocognitive disorder with Lewy bodies | 17 (0.0%) | 21 (0.0%) | 0.00 | 12 (0.0%) | 19 (0.0%) | 0.00 |
| Neoplasms | 29794 (15.7%) | 40066 (12.8%) | 0.08 | 26945 (15.5%) | 30393 (17.5%) | 0.05 |
| Malignant neoplasms of lymphoid, hematopoietic and related tissue | 1662 (0.9%) | 2354 (0.8%) | 0.01 | 1536 (0.9%) | 1814 (1.0%) | 0.02 |
| Rheumatoid arthritis with rheumatoid factor | 675 (0.4%) | 813 (0.3%) | 0.02 | 623 (0.4%) | 676 (0.4%) | 0.01 |
| Other rheumatoid arthritis | 2068 (1.1%) | 2733 (0.9%) | 0.02 | 1883 (1.1%) | 2097 (1.2%) | 0.01 |
| Systemic lupus erythematosus (SLE) | 774 (0.4%) | 865 (0.3%) | 0.02 | 678 (0.4%) | 725 (0.4%) | 0.00 |
| Psoriasis | 1824 (1.0%) | 2334 (0.7%) | 0.02 | 1661 (1.0%) | 1858 (1.1%) | 0.01 |
| Certain disorders involving the immune mechanism | 3265 (1.7%) | 4188 (1.3%) | 0.03 | 2882 (1.7%) | 2980 (1.7%) | 0.00 |
| Family history of mental and behavioral disorders | 783 (0.4%) | 2855 (0.9%) | 0.06 | 764 (0.4%) | 897 (0.5%) | 0.01 |
| Persons with potential health hazards related to socioeconomic and psychosocial circumstances | 3989 (2.1%) | 8118 (2.6%) | 0.03 | 3734 (2.2%) | 3881 (2.2%) | 0.01 |
| Personal history of other mental and behavioral disorders | 400 (0.2%) | 641 (0.2%) | 0.00 | 363 (0.2%) | 404 (0.2%) | 0.01 |
| Nicotine dependence | 12734 (6.7%) | 15914 (5.1%) | 0.07 | 11401 (6.6%) | 12726 (7.3%) | 0.03 |
| Alcohol related disorders | 3069 (1.6%) | 3133 (1.0%) | 0.05 | 2620 (1.5%) | 2671 (1.5%) | 0.00 |
| Other psychoactive substance related disorders | 1769 (0.9%) | 2275 (0.7%) | 0.02 | 1561 (0.9%) | 1660 (1.0%) | 0.01 |
| Cannabis related disorders | 1753 (0.9%) | 1416 (0.5%) | 0.06 | 1424 (0.8%) | 1309 (0.8%) | 0.01 |
| Other stimulant related disorders | 482 (0.3%) | 697 (0.2%) | 0.01 | 439 (0.3%) | 502 (0.3%) | 0.01 |
| Opioid related disorders | 915 (0.5%) | 975 (0.3%) | 0.03 | 780 (0.4%) | 819 (0.5%) | 0.00 |
| Cocaine related disorders | 584 (0.3%) | 479 (0.2%) | 0.03 | 474 (0.3%) | 442 (0.3%) | 0.00 |
| Hallucinogen related disorders | 104 (0.1%) | 102 (0.0%) | 0.01 | 84 (0.0%) | 82 (0.0%) | 0.00 |
| Inhalant related disorders | 268 (0.1%) | 272 (0.1%) | 0.02 | 226 (0.1%) | 231 (0.1%) | 0.00 |
| Sedative, hypnotic, or anxiolytic related disorders | 113 (0.1%) | 156 (0.1%) | 0.00 | 94 (0.1%) | 101 (0.1%) | 0.00 |
| Unspecified psychosis not due to a substance or known physiological condition | 252 (0.1%) | 269 (0.1%) | 0.01 | 219 (0.1%) | 219 (0.1%) | 0.00 |
| Schizophrenia | 224 (0.1%) | 246 (0.1%) | 0.01 | 198 (0.1%) | 204 (0.1%) | 0.00 |
| Schizoaffective disorders | 109 (0.1%) | 84 (0.0%) | 0.01 | 89 (0.1%) | 82 (0.0%) | 0.00 |
| Delusional disorders | 89 (0.0%) | 88 (0.0%) | 0.01 | 74 (0.0%) | 76 (0.0%) | 0.00 |
| Brief psychotic disorder | 31 (0.0%) | 42 (0.0%) | 0.00 | 28 (0.0%) | 33 (0.0%) | 0.00 |
| Other psychotic disorder not due to a substance or known physiological condition | 10 (0.0%) | 12 (0.0%) | 0.00 | 10 (0.0%) | 10 (0.0%) | 0.00 |
| Schizotypal disorder | 10 (0.0%) | 10 (0.0%) | 0.00 | 10 (0.0%) | 10 (0.0%) | 0.00 |
| Shared psychotic disorder | 10 (0.0%) | 10 (0.0%) | 0.00 | 10 (0.0%) | 10 (0.0%) | 0.00 |
| Renal Transplantation Procedures | 339 (0.2%) | 131 (0.0%) | 0.04 | 221 (0.1%) | 129 (0.1%) | 0.02 |
| Liver Transplantation Procedures | 53 (0.0%) | 40 (0.0%) | 0.01 | 42 (0.0%) | 35 (0.0%) | 0.00 |
| Blood Pressure, Systolic | | | | | | |
| <140 mm[Hg] | 72867 (38.3%) | 134928 (43.2%) | 0.10 | 68092 (39.2%) | 75826 (43.7%) | 0.09 |
| 140 - 160 mm[Hg] | 35285 (18.6%) | 43192 (13.8%) | 0.13 | 31798 (18.3%) | 35783 (20.6%) | 0.06 |
| >160[Hg] | 17348 (9.1%) | 19828 (6.3%) | 0.10 | 15456 (8.9%) | 17005 (9.8%) | 0.03 |
| Blood Pressure, Diastolic | | | | | | |
| 0 - 90 mm[Hg] | 74599 (39.2%) | 136632 (43.7%) | 0.09 | 69583 (40.1%) | 77351 (44.6%) | 0.09 |
| 90 - 100 mm[Hg] | 26744 (14.1%) | 32601 (10.4%) | 0.11 | 24004 (13.8%) | 26682 (15.4%) | 0.04 |
| 100 - 0 mm[Hg] | 12034 (6.3%) | 13152 (4.2%) | 0.09 | 10528 (6.1%) | 11290 (6.5%) | 0.02 |
| BMI | | | | | | |
| 0 - 25 kg/m2 | 32300 (17.0%) | 73624 (23.6%) | 0.16 | 30636 (17.7%) | 31097 (17.9%) | 0.01 |
| 25 - 30 kg/m2 | 27122 (14.3%) | 30235 (9.7%) | 0.14 | 23904 (13.8%) | 25518 (14.7%) | 0.03 |
| 30 - 0 kg/m2 | 27051 (14.2%) | 27924 (8.9%) | 0.17 | 23594 (13.6%) | 24946 (14.4%) | 0.02 |
| New or Established Patient | 10374 (5.5%) | 14113 (4.5%) | 0.04 | 9269 (5.3%) | 9832 (5.7%) | 0.01 |
| Hospital Inpatient Services | 13678 (7.2%) | 19909 (6.4%) | 0.03 | 12285 (7.1%) | 13007 (7.5%) | 0.02 |
| Critical Care Services | 3350 (1.8%) | 4801 (1.5%) | 0.02 | 2975 (1.7%) | 3179 (1.8%) | 0.01 |
| Visit: Inpatient Encounter | 36331 (19.1%) | 53529 (17.1%) | 0.05 | 32870 (18.9%) | 34791 (20.0%) | 0.03 |
| Visit: Short Stay | 2919 (1.5%) | 2911 (0.9%) | 0.05 | 2479 (1.4%) | 2390 (1.4%) | 0.00 |
| Visit: Inpatient Non-acute | 172 (0.1%) | 506 (0.2%) | 0.02 | 163 (0.1%) | 216 (0.1%) | 0.01 |
| Severe acute respiratory syndrome coronavirus 2 (SARS-CoV-2) (coronavirus disease [COVID-19]) vaccine, mRNA-LNP, spike protein, preservative free, 30 mcg/0.3 mL dosage, diluent reconstituted, for intramuscular use | 11389 (6.0%) | 12013 (3.8%) | 0.10 | 9927 (5.7%) | 10385 (6.0%) | 0.01 |
| Immunization administration by intramuscular injection of severe acute respiratory syndrome coronavirus 2 (SARS-CoV-2) (coronavirus disease [COVID-19]) vaccine, mRNA-LNP, spike protein, preservative free, 30 mcg/0.3 mL dosage, diluent reconstituted; first dose | 10432 (5.5%) | 10808 (3.5%) | 0.10 | 9059 (5.2%) | 9393 (5.4%) | 0.01 |
| Immunization administration by intramuscular injection of severe acute respiratory syndrome coronavirus 2 (SARS-CoV-2) (coronavirus disease [COVID-19]) vaccine, mRNA-LNP, spike protein, preservative free, 30 mcg/0.3 mL dosage, diluent reconstituted; second dose | 9906 (5.2%) | 9972 (3.2%) | 0.10 | 8556 (4.9%) | 8821 (5.1%) | 0.01 |
| Severe acute respiratory syndrome coronavirus 2 (SARS-CoV-2) (coronavirus disease [COVID-19]) vaccine, mRNA-LNP, spike protein, preservative free, 100 mcg/0.5 mL dosage, for intramuscular use | 1902 (1.0%) | 1840 (0.6%) | 0.05 | 1627 (0.9%) | 1605 (0.9%) | 0.00 |
| Immunization administration by intramuscular injection of severe acute respiratory syndrome coronavirus 2 (SARS-CoV-2) (coronavirus disease [COVID-19]) vaccine, mRNA-LNP, spike protein, preservative free, 100 mcg/0.5 mL dosage; first dose | 1837 (1.0%) | 1689 (0.5%) | 0.05 | 1556 (0.9%) | 1521 (0.9%) | 0.00 |
| Immunization administration by intramuscular injection of severe acute respiratory syndrome coronavirus 2 (SARS-CoV-2) (coronavirus disease [COVID-19]) vaccine, mRNA-LNP, spike protein, preservative free, 100 mcg/0.5 mL dosage; second dose | 1721 (0.9%) | 1611 (0.5%) | 0.05 | 1465 (0.8%) | 1417 (0.8%) | 0.00 |
| Severe acute respiratory syndrome coronavirus 2 (SARS-CoV-2) (coronavirus disease [COVID-19]) vaccine, DNA, spike protein, chimpanzee adenovirus Oxford 1 (ChAdOx1) vector, preservative free, 5x1010 viral particles/0.5 mL dosage, for intramuscular use | 0 (0.0%) | 0 (0.0%) |  | 0 (0.0%) | 0 (0.0%) |  |
| Immunization administration by intramuscular injection of severe acute respiratory syndrome coronavirus 2 (SARS-CoV-2) (coronavirus disease [COVID-19]) vaccine, DNA, spike protein, chimpanzee adenovirus Oxford 1 (ChAdOx1) vector, preservative free, 5x1010 viral particles/0.5 mL dosage; first dose | 0 (0.0%) | 0 (0.0%) |  | 0 (0.0%) | 0 (0.0%) |  |
| Immunization administration by intramuscular injection of severe acute respiratory syndrome coronavirus 2 (SARS-CoV-2) (coronavirus disease [COVID-19]) vaccine, DNA, spike protein, chimpanzee adenovirus Oxford 1 (ChAdOx1) vector, preservative free, 5x1010 viral particles/0.5 mL dosage; second dose | 0 (0.0%) | 0 (0.0%) |  | 0 (0.0%) | 0 (0.0%) |  |
| Severe acute respiratory syndrome coronavirus 2 (SARS-CoV-2) (coronavirus disease [COVID-19]) vaccine, DNA, spike protein, adenovirus type 26 (Ad26) vector, preservative free, 5x1010 viral particles/0.5 mL dosage, for intramuscular use | 185 (0.1%) | 292 (0.1%) | 0.00 | 171 (0.1%) | 185 (0.1%) | 0.00 |
| Immunization administration by intramuscular injection of severe acute respiratory syndrome coronavirus 2 (SARS-CoV-2) (coronavirus disease [COVID-19]) vaccine, DNA, spike protein, adenovirus type 26 (Ad26) vector, preservative free, 5x1010 viral particles/0.5 mL dosage, single dose | 182 (0.1%) | 291 (0.1%) | 0.00 | 168 (0.1%) | 185 (0.1%) | 0.00 |
| Introduction of COVID-19 Vaccine into Subcutaneous Tissue, Percutaneous Approach, New Technology Group 6 | 0 (0.0%) | 0 (0.0%) |  | 0 (0.0%) | 0 (0.0%) |  |
| Introduction of COVID-19 Vaccine Dose 1 into Subcutaneous Tissue, Percutaneous Approach, New Technology Group 6 | 10 (0.0%) | 10 (0.0%) | 0.00 | 10 (0.0%) | 0 (0.0%) | 0.01 |
| Introduction of COVID-19 Vaccine Dose 2 into Subcutaneous Tissue, Percutaneous Approach, New Technology Group 6 | 0 (0.0%) | 0 (0.0%) |  | 0 (0.0%) | 0 (0.0%) |  |
| Introduction of COVID-19 Vaccine into Muscle, Percutaneous Approach, New Technology Group 6 | 10 (0.0%) | 11 (0.0%) | 0.00 | 10 (0.0%) | 10 (0.0%) | 0.00 |
| Introduction of COVID-19 Vaccine Dose 1 into Muscle, Percutaneous Approach, New Technology Group 6 | 11 (0.0%) | 12 (0.0%) | 0.00 | 10 (0.0%) | 11 (0.0%) | 0.00 |
| Introduction of COVID-19 Vaccine Dose 2 into Muscle, Percutaneous Approach, New Technology Group 6 | 10 (0.0%) | 10 (0.0%) | 0.00 | 10 (0.0%) | 10 (0.0%) | 0.00 |
| SARS-CoV-2 (COVID-19) Vaccine | 12517 (6.6%) | 14566 (4.7%) | 0.08 | 10968 (6.3%) | 11443 (6.6%) | 0.01 |

**Table 9. Baseline Characteristics for Block 9 Before and After Matching**

|  | Before matching | | | After matching | | |
| --- | --- | --- | --- | --- | --- | --- |
|  | Cohort, No. (%) | | | Cohort, No. (%) | | |
| Characteristics | COVID-19 | Other RTI | SMD | COVID-19 | Other RTI | SMD |
| Total number | 121332 | 241500 |  | 118530 | 118530 |  |
| Age at index, mean (SD), y | 40.3 (23.9) | 28.9 (25.7) | 0.46 | 39.7 (23.7) | 40.8 (25.0) | 0.04 |
| Current age, mean (SD), y | 41.3 (23.9) | 29.9 (25.7) | 0.46 | 40.7 (23.7) | 41.8 (25.0) | 0.04 |
| Gender | | | | | | |
| Female | 63704 (52.5%) | 128541 (53.2%) | 0.01 | 62466 (52.7%) | 64168 (54.1%) | 0.03 |
| Male | 57569 (47.4%) | 112928 (46.8%) | 0.01 | 56027 (47.3%) | 54332 (45.8%) | 0.03 |
| Unknown | 59 (0.0%) | 31 (0.0%) | 0.02 | 37 (0.0%) | 30 (0.0%) | 0.00 |
| Race | | | | | | |
| White | 78413 (64.6%) | 161240 (66.8%) | 0.05 | 76950 (64.9%) | 78712 (66.4%) | 0.03 |
| Black or African American | 19116 (15.8%) | 33716 (14.0%) | 0.05 | 18400 (15.5%) | 17497 (14.8%) | 0.02 |
| Asian | 3617 (3.0%) | 6295 (2.6%) | 0.02 | 3503 (3.0%) | 3375 (2.8%) | 0.01 |
| American Indian or Alaska Native | 391 (0.3%) | 886 (0.4%) | 0.01 | 381 (0.3%) | 391 (0.3%) | 0.00 |
| Native Hawaiian or Other Pacific  Islander | 200 (0.2%) | 442 (0.2%) | 0.00 | 196 (0.2%) | 195 (0.2%) | 0.00 |
| Unknown race | 19595 (16.2%) | 38921 (16.1%) | 0.00 | 19100 (16.1%) | 18360 (15.5%) | 0.02 |
| Ethnicity | | | | | | |
| Hispanic or Latino | 13486 (11.1%) | 32274 (13.4%) | 0.07 | 13239 (11.2%) | 12243 (10.3%) | 0.03 |
| Not Hispanic or Latino | 82775 (68.2%) | 167206 (69.2%) | 0.02 | 81044 (68.4%) | 82336 (69.5%) | 0.02 |
| Unknown ethnicity | 25071 (20.7%) | 42020 (17.4%) | 0.08 | 24247 (20.5%) | 23951 (20.2%) | 0.01 |
| Overweight and obesity | 16077 (13.3%) | 26317 (10.9%) | 0.07 | 15553 (13.1%) | 16256 (13.7%) | 0.02 |
| Hypertensive diseases | 27914 (23.0%) | 39415 (16.3%) | 0.17 | 26857 (22.7%) | 27371 (23.1%) | 0.01 |
| Type 1 diabetes mellitus | 1579 (1.3%) | 1986 (0.8%) | 0.05 | 1456 (1.2%) | 1426 (1.2%) | 0.00 |
| Type 2 diabetes mellitus | 12317 (10.2%) | 16608 (6.9%) | 0.12 | 11709 (9.9%) | 11647 (9.8%) | 0.00 |
| Bronchitis, not specified as acute or chronic | 4425 (3.6%) | 10715 (4.4%) | 0.04 | 4372 (3.7%) | 4797 (4.0%) | 0.02 |
| Simple and mucopurulent chronic bronchitis | 348 (0.3%) | 955 (0.4%) | 0.02 | 346 (0.3%) | 397 (0.3%) | 0.01 |
| Unspecified chronic bronchitis | 360 (0.3%) | 988 (0.4%) | 0.02 | 356 (0.3%) | 398 (0.3%) | 0.01 |
| Emphysema | 1627 (1.3%) | 3246 (1.3%) | 0.00 | 1603 (1.4%) | 1698 (1.4%) | 0.01 |
| Other chronic obstructive pulmonary disease | 3632 (3.0%) | 7539 (3.1%) | 0.01 | 3586 (3.0%) | 3870 (3.3%) | 0.01 |
| Asthma | 11857 (9.8%) | 30682 (12.7%) | 0.09 | 11759 (9.9%) | 12627 (10.7%) | 0.02 |
| Bronchiectasis | 629 (0.5%) | 1649 (0.7%) | 0.02 | 624 (0.5%) | 695 (0.6%) | 0.01 |
| Other forms of heart disease | 17123 (14.1%) | 25423 (10.5%) | 0.11 | 16399 (13.8%) | 16518 (13.9%) | 0.00 |
| Hypertensive chronic kidney disease | 3171 (2.6%) | 3088 (1.3%) | 0.10 | 2736 (2.3%) | 2469 (2.1%) | 0.02 |
| Hepatic failure, not elsewhere classified | 502 (0.4%) | 489 (0.2%) | 0.04 | 447 (0.4%) | 401 (0.3%) | 0.01 |
| Chronic hepatitis, not elsewhere classified | 82 (0.1%) | 129 (0.1%) | 0.01 | 80 (0.1%) | 78 (0.1%) | 0.00 |
| Fibrosis and cirrhosis of liver | 1061 (0.9%) | 1216 (0.5%) | 0.04 | 972 (0.8%) | 895 (0.8%) | 0.01 |
| Fatty (change of) liver, not elsewhere classified | 3074 (2.5%) | 4304 (1.8%) | 0.05 | 2965 (2.5%) | 3044 (2.6%) | 0.00 |
| Chronic passive congestion of liver | 406 (0.3%) | 713 (0.3%) | 0.01 | 394 (0.3%) | 424 (0.4%) | 0.00 |
| Portal hypertension | 458 (0.4%) | 410 (0.2%) | 0.04 | 392 (0.3%) | 351 (0.3%) | 0.01 |
| Other specified diseases of liver | 1949 (1.6%) | 2955 (1.2%) | 0.03 | 1848 (1.6%) | 1904 (1.6%) | 0.00 |
| Cerebral infarction | 2582 (2.1%) | 3791 (1.6%) | 0.04 | 2447 (2.1%) | 2432 (2.1%) | 0.00 |
| Vascular dementia | 152 (0.1%) | 134 (0.1%) | 0.02 | 128 (0.1%) | 111 (0.1%) | 0.00 |
| Dementia in other diseases classified elsewhere | 316 (0.3%) | 341 (0.1%) | 0.03 | 289 (0.2%) | 266 (0.2%) | 0.00 |
| Unspecified dementia | 645 (0.5%) | 695 (0.3%) | 0.04 | 574 (0.5%) | 535 (0.5%) | 0.00 |
| Alzheimer's disease | 280 (0.2%) | 307 (0.1%) | 0.02 | 254 (0.2%) | 241 (0.2%) | 0.00 |
| Frontotemporal dementia | 23 (0.0%) | 19 (0.0%) | 0.01 | 19 (0.0%) | 17 (0.0%) | 0.00 |
| Neurocognitive disorder with Lewy bodies | 23 (0.0%) | 24 (0.0%) | 0.01 | 22 (0.0%) | 18 (0.0%) | 0.00 |
| Neoplasms | 21499 (17.7%) | 34853 (14.4%) | 0.09 | 20856 (17.6%) | 21760 (18.4%) | 0.02 |
| Malignant neoplasms of lymphoid, hematopoietic and related tissue | 1734 (1.4%) | 2183 (0.9%) | 0.05 | 1639 (1.4%) | 1597 (1.3%) | 0.00 |
| Rheumatoid arthritis with rheumatoid factor | 596 (0.5%) | 758 (0.3%) | 0.03 | 577 (0.5%) | 566 (0.5%) | 0.00 |
| Other rheumatoid arthritis | 1675 (1.4%) | 2520 (1.0%) | 0.03 | 1618 (1.4%) | 1630 (1.4%) | 0.00 |
| Systemic lupus erythematosus (SLE) | 589 (0.5%) | 756 (0.3%) | 0.03 | 560 (0.5%) | 551 (0.5%) | 0.00 |
| Psoriasis | 1240 (1.0%) | 2049 (0.8%) | 0.02 | 1212 (1.0%) | 1281 (1.1%) | 0.01 |
| Certain disorders involving the immune mechanism | 3236 (2.7%) | 3839 (1.6%) | 0.07 | 2854 (2.4%) | 2675 (2.3%) | 0.01 |
| Family history of mental and behavioral disorders | 629 (0.5%) | 2242 (0.9%) | 0.05 | 616 (0.5%) | 676 (0.6%) | 0.01 |
| Persons with potential health hazards related to socioeconomic and psychosocial circumstances | 2455 (2.0%) | 6702 (2.8%) | 0.05 | 2409 (2.0%) | 2471 (2.1%) | 0.00 |
| Personal history of other mental and behavioral disorders | 314 (0.3%) | 557 (0.2%) | 0.01 | 296 (0.3%) | 301 (0.3%) | 0.00 |
| Nicotine dependence | 7867 (6.5%) | 13629 (5.6%) | 0.04 | 7695 (6.5%) | 8116 (6.8%) | 0.01 |
| Alcohol related disorders | 1903 (1.6%) | 2748 (1.1%) | 0.04 | 1825 (1.5%) | 1817 (1.5%) | 0.00 |
| Other psychoactive substance related disorders | 1148 (0.9%) | 1856 (0.8%) | 0.02 | 1097 (0.9%) | 1102 (0.9%) | 0.00 |
| Cannabis related disorders | 884 (0.7%) | 1169 (0.5%) | 0.03 | 843 (0.7%) | 829 (0.7%) | 0.00 |
| Other stimulant related disorders | 342 (0.3%) | 573 (0.2%) | 0.01 | 336 (0.3%) | 355 (0.3%) | 0.00 |
| Opioid related disorders | 598 (0.5%) | 859 (0.4%) | 0.02 | 578 (0.5%) | 581 (0.5%) | 0.00 |
| Cocaine related disorders | 305 (0.3%) | 460 (0.2%) | 0.01 | 295 (0.2%) | 293 (0.2%) | 0.00 |
| Hallucinogen related disorders | 70 (0.1%) | 101 (0.0%) | 0.01 | 68 (0.1%) | 58 (0.0%) | 0.00 |
| Inhalant related disorders | 178 (0.1%) | 247 (0.1%) | 0.01 | 174 (0.1%) | 171 (0.1%) | 0.00 |
| Sedative, hypnotic, or anxiolytic related disorders | 85 (0.1%) | 145 (0.1%) | 0.00 | 85 (0.1%) | 81 (0.1%) | 0.00 |
| Unspecified psychosis not due to a substance or known physiological condition | 200 (0.2%) | 276 (0.1%) | 0.01 | 188 (0.2%) | 189 (0.2%) | 0.00 |
| Schizophrenia | 152 (0.1%) | 239 (0.1%) | 0.01 | 144 (0.1%) | 149 (0.1%) | 0.00 |
| Schizoaffective disorders | 82 (0.1%) | 78 (0.0%) | 0.02 | 75 (0.1%) | 71 (0.1%) | 0.00 |
| Delusional disorders | 53 (0.0%) | 78 (0.0%) | 0.01 | 49 (0.0%) | 52 (0.0%) | 0.00 |
| Brief psychotic disorder | 20 (0.0%) | 24 (0.0%) | 0.01 | 19 (0.0%) | 14 (0.0%) | 0.00 |
| Other psychotic disorder not due to a substance or known physiological condition | 10 (0.0%) | 10 (0.0%) | 0.01 | 10 (0.0%) | 10 (0.0%) | 0.00 |
| Schizotypal disorder | 0 (0.0%) | 0 (0.0%) |  | 0 (0.0%) | 0 (0.0%) |  |
| Shared psychotic disorder | 0 (0.0%) | 0 (0.0%) |  | 0 (0.0%) | 0 (0.0%) |  |
| Renal Transplantation Procedures | 432 (0.4%) | 150 (0.1%) | 0.06 | 222 (0.2%) | 149 (0.1%) | 0.02 |
| Liver Transplantation Procedures | 80 (0.1%) | 50 (0.0%) | 0.02 | 67 (0.1%) | 45 (0.0%) | 0.01 |
| Blood Pressure, Systolic | | | | | | |
| <140 mm[Hg] | 50450 (41.6%) | 110768 (45.9%) | 0.09 | 49531 (41.8%) | 51430 (43.4%) | 0.03 |
| 140 - 160 mm[Hg] | 24803 (20.4%) | 37673 (15.6%) | 0.13 | 24023 (20.3%) | 25031 (21.1%) | 0.02 |
| >160[Hg] | 12832 (10.6%) | 17845 (7.4%) | 0.11 | 12280 (10.4%) | 12318 (10.4%) | 0.00 |
| Blood Pressure, Diastolic | | | | | | |
| 0 - 90 mm[Hg] | 51485 (42.4%) | 112277 (46.5%) | 0.08 | 50542 (42.6%) | 52515 (44.3%) | 0.03 |
| 90 - 100 mm[Hg] | 18520 (15.3%) | 28224 (11.7%) | 0.10 | 17879 (15.1%) | 18527 (15.6%) | 0.02 |
| 100 - 0 mm[Hg] | 8550 (7.0%) | 11507 (4.8%) | 0.10 | 8120 (6.9%) | 8144 (6.9%) | 0.00 |
| BMI | | | | | | |
| 0 - 25 kg/m2 | 22323 (18.4%) | 56835 (23.5%) | 0.13 | 21943 (18.5%) | 21214 (17.9%) | 0.02 |
| 25 - 30 kg/m2 | 18087 (14.9%) | 25441 (10.5%) | 0.13 | 17321 (14.6%) | 17549 (14.8%) | 0.01 |
| 30 - 0 kg/m2 | 17734 (14.6%) | 24197 (10.0%) | 0.14 | 16988 (14.3%) | 17192 (14.5%) | 0.00 |
| New or Established Patient | 8213 (6.8%) | 13117 (5.4%) | 0.06 | 7753 (6.5%) | 7648 (6.5%) | 0.00 |
| Hospital Inpatient Services | 10522 (8.7%) | 17954 (7.4%) | 0.05 | 9999 (8.4%) | 9984 (8.4%) | 0.00 |
| Critical Care Services | 2741 (2.3%) | 4685 (1.9%) | 0.02 | 2621 (2.2%) | 2620 (2.2%) | 0.00 |
| Visit: Inpatient Encounter | 23972 (19.8%) | 43370 (18.0%) | 0.05 | 23117 (19.5%) | 23227 (19.6%) | 0.00 |
| Visit: Short Stay | 1983 (1.6%) | 2146 (0.9%) | 0.07 | 1755 (1.5%) | 1568 (1.3%) | 0.01 |
| Visit: Inpatient Non-acute | 189 (0.2%) | 441 (0.2%) | 0.01 | 185 (0.2%) | 186 (0.2%) | 0.00 |
| Severe acute respiratory syndrome coronavirus 2 (SARS-CoV-2) (coronavirus disease [COVID-19]) vaccine, mRNA-LNP, spike protein, preservative free, 30 mcg/0.3 mL dosage, diluent reconstituted, for intramuscular use | 7858 (6.5%) | 11038 (4.6%) | 0.08 | 7476 (6.3%) | 7459 (6.3%) | 0.00 |
| Immunization administration by intramuscular injection of severe acute respiratory syndrome coronavirus 2 (SARS-CoV-2) (coronavirus disease [COVID-19]) vaccine, mRNA-LNP, spike protein, preservative free, 30 mcg/0.3 mL dosage, diluent reconstituted; first dose | 6748 (5.6%) | 9307 (3.9%) | 0.08 | 6393 (5.4%) | 6327 (5.3%) | 0.00 |
| Immunization administration by intramuscular injection of severe acute respiratory syndrome coronavirus 2 (SARS-CoV-2) (coronavirus disease [COVID-19]) vaccine, mRNA-LNP, spike protein, preservative free, 30 mcg/0.3 mL dosage, diluent reconstituted; second dose | 6500 (5.4%) | 8588 (3.6%) | 0.09 | 6136 (5.2%) | 6031 (5.1%) | 0.00 |
| Severe acute respiratory syndrome coronavirus 2 (SARS-CoV-2) (coronavirus disease [COVID-19]) vaccine, mRNA-LNP, spike protein, preservative free, 100 mcg/0.5 mL dosage, for intramuscular use | 1153 (1.0%) | 1641 (0.7%) | 0.03 | 1118 (0.9%) | 1107 (0.9%) | 0.00 |
| Immunization administration by intramuscular injection of severe acute respiratory syndrome coronavirus 2 (SARS-CoV-2) (coronavirus disease [COVID-19]) vaccine, mRNA-LNP, spike protein, preservative free, 100 mcg/0.5 mL dosage; first dose | 1072 (0.9%) | 1490 (0.6%) | 0.03 | 1037 (0.9%) | 1029 (0.9%) | 0.00 |
| Immunization administration by intramuscular injection of severe acute respiratory syndrome coronavirus 2 (SARS-CoV-2) (coronavirus disease [COVID-19]) vaccine, mRNA-LNP, spike protein, preservative free, 100 mcg/0.5 mL dosage; second dose | 1018 (0.8%) | 1414 (0.6%) | 0.03 | 985 (0.8%) | 976 (0.8%) | 0.00 |
| Severe acute respiratory syndrome coronavirus 2 (SARS-CoV-2) (coronavirus disease [COVID-19]) vaccine, DNA, spike protein, chimpanzee adenovirus Oxford 1 (ChAdOx1) vector, preservative free, 5x1010 viral particles/0.5 mL dosage, for intramuscular use | 0 (0.0%) | 0 (0.0%) |  | 0 (0.0%) | 0 (0.0%) |  |
| Immunization administration by intramuscular injection of severe acute respiratory syndrome coronavirus 2 (SARS-CoV-2) (coronavirus disease [COVID-19]) vaccine, DNA, spike protein, chimpanzee adenovirus Oxford 1 (ChAdOx1) vector, preservative free, 5x1010 viral particles/0.5 mL dosage; first dose | 0 (0.0%) | 0 (0.0%) |  | 0 (0.0%) | 0 (0.0%) |  |
| Immunization administration by intramuscular injection of severe acute respiratory syndrome coronavirus 2 (SARS-CoV-2) (coronavirus disease [COVID-19]) vaccine, DNA, spike protein, chimpanzee adenovirus Oxford 1 (ChAdOx1) vector, preservative free, 5x1010 viral particles/0.5 mL dosage; second dose | 0 (0.0%) | 0 (0.0%) |  | 0 (0.0%) | 0 (0.0%) |  |
| Severe acute respiratory syndrome coronavirus 2 (SARS-CoV-2) (coronavirus disease [COVID-19]) vaccine, DNA, spike protein, adenovirus type 26 (Ad26) vector, preservative free, 5x1010 viral particles/0.5 mL dosage, for intramuscular use | 104 (0.1%) | 211 (0.1%) | 0.00 | 103 (0.1%) | 114 (0.1%) | 0.00 |
| Immunization administration by intramuscular injection of severe acute respiratory syndrome coronavirus 2 (SARS-CoV-2) (coronavirus disease [COVID-19]) vaccine, DNA, spike protein, adenovirus type 26 (Ad26) vector, preservative free, 5x1010 viral particles/0.5 mL dosage, single dose | 103 (0.1%) | 206 (0.1%) | 0.00 | 102 (0.1%) | 111 (0.1%) | 0.00 |
| Introduction of COVID-19 Vaccine into Subcutaneous Tissue, Percutaneous Approach, New Technology Group 6 | 0 (0.0%) | 10 (0.0%) | 0.01 | 0 (0.0%) | 10 (0.0%) | 0.01 |
| Introduction of COVID-19 Vaccine Dose 1 into Subcutaneous Tissue, Percutaneous Approach, New Technology Group 6 | 0 (0.0%) | 10 (0.0%) | 0.01 | 0 (0.0%) | 10 (0.0%) | 0.01 |
| Introduction of COVID-19 Vaccine Dose 2 into Subcutaneous Tissue, Percutaneous Approach, New Technology Group 6 | 0 (0.0%) | 0 (0.0%) |  | 0 (0.0%) | 0 (0.0%) |  |
| Introduction of COVID-19 Vaccine into Muscle, Percutaneous Approach, New Technology Group 6 | 15 (0.0%) | 22 (0.0%) | 0.00 | 13 (0.0%) | 14 (0.0%) | 0.00 |
| Introduction of COVID-19 Vaccine Dose 1 into Muscle, Percutaneous Approach, New Technology Group 6 | 12 (0.0%) | 11 (0.0%) | 0.01 | 11 (0.0%) | 10 (0.0%) | 0.00 |
| Introduction of COVID-19 Vaccine Dose 2 into Muscle, Percutaneous Approach, New Technology Group 6 | 10 (0.0%) | 10 (0.0%) | 0.01 | 10 (0.0%) | 10 (0.0%) | 0.00 |
| SARS-CoV-2 (COVID-19) Vaccine | 9355 (7.7%) | 12381 (5.1%) | 0.11 | 8781 (7.4%) | 8534 (7.2%) | 0.01 |
